# Disentangling the potential effects of land‐use and climate change on stream conditions

**DOI:** 10.1111/gcb.14961

**Published:** 2020-01-19

**Authors:** Kelly O. Maloney, Kevin P. Krause, Claire Buchanan, Lauren E. Hay, Gregory J. McCabe, Zachary M. Smith, Terry L. Sohl, John A. Young

**Affiliations:** ^1^ U.S. Geological Survey Leetown Science Center Kearneysville WV USA; ^2^ Interstate Commission on the Potomac River Basin (ICPRB) Rockville MD USA; ^3^ U.S. Geological Survey Denver Federal Center Denver CO USA; ^4^ U.S. Geological Survey Earth Resources Observation and Science (EROS) Center Sioux Falls SD USA; ^5^ Present address: New England Interstate Water Pollution Control Commission (NEIWPCC) c/o New York State DEC 625 Broadway, 4th Floor Albany NY 12233 USA

**Keywords:** benthic macroinvertebrates, Chesapeake Bay watershed, Chessie BIBI, prediction, projection

## Abstract

Land‐use and climate change are significantly affecting stream ecosystems, yet understanding of their long‐term impacts is hindered by the few studies that have simultaneously investigated their interaction and high variability among future projections. We modeled possible effects of a suite of 2030, 2060, and 2090 land‐use and climate scenarios on the condition of 70,772 small streams in the Chesapeake Bay watershed, United States. The Chesapeake Basin‐wide Index of Biotic Integrity, a benthic macroinvertebrate multimetric index, was used to represent stream condition. Land‐use scenarios included four Special Report on Emissions Scenarios (A1B, A2, B1, and B2) representing a range of potential landscape futures. Future climate scenarios included quartiles of future climate changes from downscaled Coupled Model Intercomparison Project ‐ Phase 5 (CMIP5) and a watershed‐wide uniform scenario (Lynch2016). We employed random forests analysis to model individual and combined effects of land‐use and climate change on stream conditions. Individual scenarios suggest that by 2090, watershed‐wide conditions may exhibit anywhere from large degradations (e.g., scenarios A1B, A2, and the CMIP5 25th percentile) to small degradations (e.g., scenarios B1, B2, and Lynch2016). Combined land‐use and climate change scenarios highlighted their interaction and predicted, by 2090, watershed‐wide degradation in 16.2% (A2 CMIP5 25th percentile) to 1.0% (B2 Lynch2016) of stream kilometers. A goal for the Chesapeake Bay watershed is to restore 10% of stream kilometers over a 2008 baseline; our results suggest meeting and sustaining this goal until 2090 may require improvement in 11.0%–26.2% of stream kilometers, dependent on land‐use and climate scenario. These results highlight inherent variability among scenarios and the resultant uncertainty of predicted conditions, which reinforces the need to incorporate multiple scenarios of both land‐use (e.g., development, agriculture, etc.) and climate change in future studies to encapsulate the range of potential future conditions.

## Graphical abstract

Maps showing small NHDplusV2 catchments (<200 km^2^ in upstream drainage) across the Chesapeake Bay watershed predicted to be in Poor, Fair, or Good condition under baseline conditions (A), the A2 economic growth land cover projection combined with median climate 2090 projections from CMIP5 scenarios (B) and the B2 environmental sustainability land cover projection combined with median climate 2090 projections from CMIP5 scenarios (C). Upper focus area centers on region near the Hornell, NY, lower focus area centers on region near York Pennsylvania, and Bel Air Maryland.

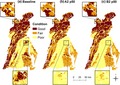

## 1 INTRODUCTION

Land‐use and climate change are two factors that have dramatically altered freshwater ecosystems across the globe (Carpenter, Stanley, & Zanden, 2011; Davidson, 2014; Meyer, Sale, Mulholland, & Poff, 1999; Vörösmarty et al., 2010; Woodward, Perkins, & Brown, 2010), resulting in a disproportionately high number of imperiled species occupying these systems (Collen et al., 2014; Strayer & Dudgeon, 2010; Young, McCauley, Galetti, & Dirzo, 2016). Managers seeking to protect and restore freshwater ecosystems into the future will need to consider both factors to implement effective conservation programs. However, land‐use and climate interactively affect ecosystems (Northrup, Rivers, Yang, & Betts, 2019; Oliver & Morecroft, 2014; Radinger et al., 2016), and thus, their individual and combined effects need to be simultaneously assessed (Carpenter et al., 2011; Meyer et al., 1999; Radinger et al., 2016).

Small streams are particularly susceptible to land‐use and climate change (Meyer et al., 1999; Woodward et al., 2010). Land‐use change has been shown to modify small stream chemistry, hydrology, geomorphology, and biology (Allan, 2004; Walsh et al., 2005), likely because of the strong coupling of streams to upstream landscapes (Hynes, 1975; Kärnä et al., 2019). Climate change also affects small streams by altering streamflow and temperature regimes (Dhungel, Tarboton, Jin, & Hawkins, 2016; Guse et al., 2015; Woodward et al., 2010). The effects of land‐use and climate change on small streams may have a proportionally large impact on global freshwater biodiversity given that small streams make up more than 88% of global freshwater stream length (≤3rd Strahler order streams, Downing et al., 2012) and provide habitat to many freshwater taxa (Meyer et al., 1999).

Benthic macroinvertebrates are a major component of small stream communities providing important functions, including nutrient and energy transfer through food webs (Covich, Palmer, & Crowl, 1999). A single stream can contain hundreds of macroinvertebrate taxa with diverse life histories and tolerances to external stressors (Meyer et al., 2007); as such, they are useful in assessing cumulative stress and are critical bioindicators in many stream monitoring programs (Bonada, Prat, Resh, & Statzner, 2006; Carter, Resh, & Hannaford, 2017). Macroinvertebrates also can be sensitive to the effects of climate change and land‐use (Durance & Ormerod, 2007; Kuemmerlen et al., 2015; Mustonen et al., 2018; Nelson et al., 2009; Pyne & Poff, 2017). Incorporating projected future land‐use and climate change into assessments of macroinvertebrates and stream condition could not only improve our understanding of their effects but it also may improve regulatory and policy decisions.

Given the complex and unpredictable interactions between land‐use and climate projections, they are not precise predictions of future conditions but rather provide a range of possible futures and future uncertainties (Oliver & Morecroft, 2014; Van Vuuren & Carter, 2014). Therefore, studies on future biological conditions need to incorporate a multi‐scenario approach that captures the range of possible future conditions. Here, our objectives were to (a) predict stream biological conditions as defined by use of the Chesapeake Basin‐wide Index of Biotic Integrity (Chessie BIBI; Smith, Buchanan, & Nagel, 2017) under a suite of land‐use and climate projections for the Chesapeake Bay watershed, and (b) use these predictions to determine the percent of stream length improvement needed under different scenarios to improve health and function of 10% of stream miles over a 2008 baseline developed using the Chessie BIBI (Chesapeake Bay Program, 2017b).

The Chesapeake Bay watershed lies in the northeast United States and by some predictions will experience a 2.0°C air temperature increase by 2035, the greatest warming in the contiguous United States and a level that is two decades ahead of global average values (Dupigny‐Giroux et al., 2018). Streams in this region may therefore experience the effects of climate change earlier than in other regions. Human population is also expected to increase by 2 million in the watershed, from 18 to 20 million, by 2030 (Chesapeake Bay Program, 2017a), which may result in drastic changes to land‐use patterns in coming decades. Together, the predicted early onset of climatic and population changes in this region makes it an excellent test case of how land‐use and climate change may affect global freshwater biological conditions.

## 2 MATERIALS AND METHODS

### 2.1 Study area

**Figure 1:**
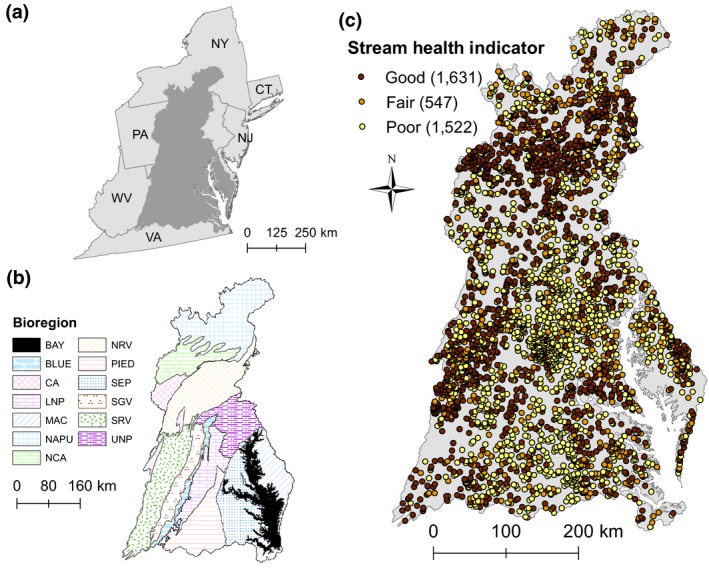
Maps showing study area in relation to the mid‐Atlantic United States (a) and bioregions (b) with Chessie BIBI scores categorized as Good, Fair, or Poor (c). Numbers within parentheses in the right panel indicate the number of samples in each category. Bioregion abbreviations: BAY = Chesapeake Bay, BLUE = Blue Ridge, CA = Central Appalachians, LNP = Lower‐Northern Piedmont, MAC = Middle Atlantic Coastal Plain, NAPU = Northern Appalachian Plateau and Uplands, NCA = North Central Appalachians, NRV = Northern Ridge and Valley, PIED = Piedmont, SEP = Southeastern Plains, SGV = Southern Great Valley, SRV = Southern Ridge and Valley, and UNP = Upper‐Northern Piedmont. State abbreviations: CT = Connecticut, NJ = New Jersey, NY = New York, PA = Pennsylvania, VA = Virginia, and WV = West Virginia

The Chesapeake Bay watershed drains approximately 168,000 km^2^ of Delaware, Maryland, New York, Pennsylvania, Virginia, West Virginia, and the District of Columbia (Figure 1). Major river basins in the watershed include the Susquehanna, Potomac, James, Rappahannock, and York, which drain into the Chesapeake Bay, the largest estuary in the United States (Chesapeake Bay Program, 2017a). The watershed currently has a population of over 18 million and includes the major cities Baltimore, Maryland; Washington, DC; Harrisburg, Pennsylvania; and Richmond, Virginia. Developed land cover comprised 11.0% and agriculture comprised 24.5% of the watershed in 2011 (2011 National Land Cover Database, NLCD, Homer et al., 2015).

### 2.2 Datasets

#### 2.2.1 NHDPlusV2

We summarized stream condition indicators and assessed land‐use and climate influences using geospatial data and geographic information system (GIS) procedures. We used the 1:100,000 National Hydrography Dataset Version 2 (NHDPlusV2, McKay et al., 2012) as a base GIS data layer to aggregate predictors. Key features of NHDPlusV2 include flowlines, local catchments, and value‐added attributes, such as flowline length, all of which are connected by a common unique identifier.

#### 2.2.2 The Chesapeake Basin‐wide Index of Biotic Integrity (Chessie BIBI)

The Chessie BIBI provides a standardized measure of stream condition for the watershed (Smith et al., 2017). It was developed using stream benthic macroinvertebrate raw counts from 21,343 sampling events collected by 28 state, county, regional, federal, and other monitoring programs from 1992 to 2015. The analysis focused on first‐ to fourth‐order streams and excluded data collected in December, January, and February because of limited surveying. Smith et al. (2017) standardized the dataset's macroinvertebrate taxonomy (e.g., excluded taxa enumerated by just one or two sampling programs) and rarefied sample counts to approximately 100 individuals per sample event prior to calculating richness and diversity metrics. Smith et al. (2017) evaluated over 200 commonly used metrics (Barbour, Gerritsen, Snyder, & Stribling, 1999), and identified those most sensitive to degradation in stream habitat and water quality conditions in the watershed's 12 geographically distinct bioregions. Each sample's metrics were scored on a continuous gradient (0–100) and averaged to produce the final index score. High scores indicate macroinvertebrate communities like those in high quality, relatively undisturbed streams (i.e., reference). Final index scores were then assigned a categorical rating of Very Poor, Poor, Fair, Good, or Excellent based on thresholds derived from the 10th, 25th, and 50th percentiles of BIBI scores in reference samples. See Smith et al., 2017 for a detailed description of the Chessie BIBI.

We used the family‐level, bioregion‐scale Chessie BIBI (Maloney, Smith, Buchanan, Nagel, & Young, 2018; Smith et al., 2017). We chose this version because it (a) optimized the trade‐off between taxonomic resolution and sample size, (b) adjusted for natural effects of geology, topography, and biogeography (i.e., bioregion), and (c) selected by stakeholders as their preferred stream health indicator (Buchanan, Maloney, Smith, Nagel, & Young, 2018). The Chessie BIBI was built 100 times using a bootstrap approach to account for slight differences in the taxa selected during the probabilistic rarefaction step, because it randomly selects which rare taxa are included in the BIBI calculation. The median result of those 100 runs was then used in modeling. In total, 21,266 stream sampling events were available with the family‐level, bioregion‐scale Chessie BIBI, of which 17,385 were from our baseline period of January 1, 2000 to December 31, 2011. This baseline was chosen to capture two complete surveys of the watershed because each survey program takes 6 years to cover the watershed (Buchanan et al., 2018). We spatially linked each sample to an NHDPlusV2 catchment. We removed 14 events due to incomplete data and 1,458 sites with upstream drainages ≥200 km^2^, the threshold for our definition of small streams, dropping the sample size to 15,913. For NHDPlusV2 catchments with more than one Chessie BIBI score, we randomly selected one sample (9,159 were duplicate events, leaving 6,754 spatially independent samples). Spatial clustering of the 6,754 samples was evident (Figure S1) because of high sampling frequency in the state of Maryland and Fairfax County, Virginia (*n* = 3,599). To reduce potential for spatial clustering to affect modeling, we subset data from the state of Maryland and Fairfax County, Virginia, to match the spatial density of unique samples throughout the rest of the watershed (0.0222 samples per km^2^). Thus, we randomly subsampled 545 samples from Maryland and Fairfax County and added these to the 3,155 samples from the rest of the Chesapeake Bay watershed for a total of 3,700 samples. These samples comprised 646 sites classified in Very Poor, 876 in Poor, 547 in Fair, 690 in Good, and 941 in Excellent condition. To align with Chesapeake Bay Program goals (Chesapeake Bay Program, 2017b), the Very Poor category was aggregated into the Poor category and the Excellent category was aggregated into the Good category; ultimately producing the three categories used here: Poor, Fair, and Good (Figure 1). Each sample in the final dataset was linked to the baseline predictor dataset by the unique NHDPlusV2 identifier.

#### 2.2.3 Physical environmental dataset

We used data from StreamCat (Hill, Weber, Leibowitz, Olsen, & Thornbrugh, 2016), which contains 517 metrics representing both natural and anthropogenic landscape information summarized using NHDPlusV2 at local, cumulative upstream and riparian scales. We selected 15 uncorrelated (*r* < 0.70) StreamCat metrics that were identified in literature as important surrogates of instream drivers of stream condition as they relate to habitat for benthic macroinvertebrates, including:

- Upstream cumulative watershed area because it is strongly related to many stream variables, such as discharge, energy process, and biological communities (Vannote, Minshall, Cummins, Sedell, & Cushing, 1980);
- Elevation since it was important in previous modeling efforts of stream condition in the study area and because it correlates with slope and instream temperature (Maloney, Smith, et al., 2018);
- Seven soil predictors (mean season water depth (cm), mean organic matter content (% by weight), mean permeability (cm/hr), mean depth (cm) to bedrock, mean percent clay content, mean percent sand content, and mean soil erodibility (Kf) factor) because of the importance of soils and resultant drivers (e.g., sediment) on stream macroinvertebrates (Waters, 1995);
- Three measures of geochemical content in surface or near surface geology—mean percent of lithological calcium oxide (CaO) because of its high correlation with many stream chemistry variables, mean lithological hydraulic conductivity (micrometers per second) because of its influence on rock/waters interaction, and mean lithological uniaxial compressive strength as a measure of susceptibility to weathering (megaPascals; Olson & Hawkins, 2012) all of which affect local habitat for macroinvertebrates;
- Summaries of mean runoff (mm) and baseflow index because of the importance of hydrology to streams (Poff et al., 1997); and
- Mean composite topographic index (topographic wetness index), which relates upslope area to local slope and is used to quantify topographic control on hydrological processes and estimate water accumulation—that is, valley bottoms have a high index whereas ridge and crests have a low index (Beven & Kirkby, 1979; Sörensen, Zinko, & Seibert, 2006). We hypothesized streams near or surrounded by ridges and crests would have less anthropogenic stress due to less accessibility or suitability, thus improving stream condition.

We used cumulative upstream watershed values for these predictors because local stream conditions are influenced by upstream catchment conditions (Scott, Helfman, McTammany, Benfield, & Bolstad, 2002) and the coarse resolution of many predictor layers can lead to inaccurate estimations when used at a local reach scale.

#### 2.2.4 Land‐use

We assessed baseline land‐use and land cover (LULC) conditions in 2005 and projected future LULC for 2030, 2060, and 2090, using an existing, consistent, annual LULC database from 1992 to 2100. Land‐use scenarios for the Chesapeake Bay watershed were extracted from existing landscape projections consistent with Intergovernmental Panel on Climate Change (IPCC) Special Report on Emissions Scenarios (SRES; Nakicenovic et al., 2000; Sohl et al., 2014). SRES are based on assumptions related to projected demographic change, energy use and sources, technological innovation, economic development, climate change, and other socioeconomic and biophysical factors (Table 1). Integrated assessment models (IAMs) are used by the IPCC to quantitatively model interactions among these factors, including impacts on land‐use (Moss et al., 2010); however, land‐use projections from IAMs are spatially coarse and unsuitable for this scale of application. Global‐level SRES projections produced by IAMs have been statistically and spatially downscaled to a regional level for the conterminous United States by Sleeter et al. (2012), while the Forecasting Scenarios of land‐use (FORE‐SCE) model was used to create spatially explicit projections consistent with SRES assumptions by Sohl et al. (2014). For this paper, we use the four modeled SRES from Sohl et al. (2014; A1B, A2, B1, and B2), representing a range of potential landscape futures. There are no comparable landscape projections (e.g., Representative Concentration Pathways [RCP]) available for the Chesapeake Bay watershed with a similar level of thematic detail (17 land‐use and land‐cover classes, 14 of which were present in the watershed, Figures S2–S5), spatial resolution (250 m pixels), and broad geographic coverage. Here, the three mechanical disturbed categories were combined into one class, MechDist, yielding 12 land‐cover categories (see Table S1 for definitions).

**Table 1:** Assumptions associated with each of the IPCC SRES land cover projections used in this study

	IPCC Special Report on Emissions Scenarios (SRES)			
	A1B	A2	B1	B2
Economic or environmental	Economic growth	Economic growth	Environmental sustainability	Environmental sustainability
Globalization/regionalization	Global convergence	Regional development	Global convergence	Regional development
US population	461 million (2100)	628 million (2100)	461 million (2100)	366 million (2100)
US per capita GDP	$146,807 (2100)	$67,536 (2100)	$92,086 (2100)	$87,616 (2100)
Energy use	Very high: balanced sources	High: regionally sourced including fossil fuels	Low: transition to post‐fossil fuel technology	Medium: regional, fossil fuel use declines over time
Technological change	Rapid pace, rapid diffusion	Slow pace, slow diffusion	Medium pace, rapid diffusion	Medium pace, uneven diffusion
Resources and land‐use	Active management rather than conservation	Uneven, but weak environmental concern, focus on consumption	Sustainable development, efficient resource use	Uneven, with local solutions to environmental protection

Note

See Nakicenovic et al. (2000) for additional details on SRES.

Sohl et al. (2014) mapped historical LULC from 1992 to 2005 using historical data sources where possible. For our baseline model, we used 2005 because it was midpoint of our baseline stream condition period. LULC projection data were available from 2006 to 2100, and these data were spatially and thematically consistent with the 1992–2005 historical data. We focused on 2030, 2060, and 2090 time periods to ensure consistent temporal intervals and to coincide with available climate data. In total, we had 13 LULC rasters—the 2005 baseline and three different future time period projections for each of the four scenarios. To optimize summation with NHDPlusV2, each LULC raster dataset (250 m pixels) was resampled to 30 m pixel resolution.

#### 2.2.5 Climate

Climatic conditions within the Chesapeake Bay watershed were incorporated by including measures of average air temperature (°C) and total precipitation (mm), both of which were calculated by season as defined by water year (January–March, April–June, July–September, October–December) given the importance of hydrology on stream conditions (Poff et al., 1997).

For baseline estimates, we used monthly climate data from the Parameter‐elevation Relationships on Independent Slopes Model (PRISM, 4 km grid pixel size, PRISM Climate Group, Oregon State University, http://www.prismclimate.org) from 2000 to 2011 to coincide with the baseline stream condition period. Monthly values were aggregated to seasonal mean temperature and seasonal total precipitation. These annual estimates were then averaged over 2000–2011 to produce 4 km rasters of baseline seasonal average temperature and average total precipitation. Each 4 km raster was then resampled to 30 m to optimize summation with NHDPlusV2.

Climate change scenarios were calculated as departures from historical average values using projected changes in seasonal average air temperature and total precipitation from Hay and McCabe (2019). This dataset summarized model projections of changes in seasonal temperature and precipitation from 122 statistically downscaled general circulation models (GCMs) climate simulations included in the World Climate Research Program's Coupled Model Intercomparison Project 5 (CMIP5) scenarios. Each CMIP5 projection is composed of a number of different scenarios (i.e., RCP 4.5, 6, and 8.5). Climate conditions represented by these scenarios range from stabilized populations after 2050, coupled with rapid development of more efficient technological systems across the globe (RCP4.5), to globally increasing populations and regionally orientated economic development (RCP8.5). For each downscaled GCM, the historical conditions (or current climatic conditions) are represented by climate model output for 1980–1999, whereas future climatic conditions are represented by model simulations for 2005–2099. Data for each of the 122 climate projections were summarized to hydrologic response unit (HRU) polygons (Viger & Bock, 2014; Figure S6). For each climate projection, future temperature and precipitation changes were summarized as departures from a 20 year historical period (1980–1999) for each HRU and aggregated as seasonal values as defined above. Departures of temperature and precipitation were expressed as mean seasonal departures for 19 year future periods centered on 2030, 2060, and 2090. For temperature and precipitation, the 25th, 50th, and 75th percentiles of projected deviations of temperature (°C) and precipitation (mm) were computed for each HRU separately for each season and period (e.g., Figures S7–S12) and then rasterized at 30 m resolution to coincide with the resampled PRISM and other covariate data. We added these deviations to historical PRISM climate estimates (1980–1999) to produce future projections of average seasonal temperature and total seasonal precipitation across the Chesapeake Bay watershed (*n* = 36 average temperature rasters and 36 total precipitation rasters). For a more detailed description of the CMIP5 processing, see Appendix S1. Lynch, Seth, and Thibeault (2016) used the CMIP5 scenarios to develop projected seasonal changes in total precipitation and average temperature in the Northeast United States. We used these uniform, generalized projections to assess whether responses among stream conditions were influenced by the localized effects of downscaling by adding the uniform projected seasonal changes in total precipitation and average temperature to all historical values (Lynch et al., 2016, see Table S2).

#### 2.2.6 Processing of predictor data

We first tabulated values for each NHDPlusV2 local catchment for each of the 13 LULC rasters, baseline 2000–2011 temperature and precipitation rasters, and 72 climate rasters from CMIP5 using GIS (ArcGIS 10.6, ESRI). For each LULC raster, local LULC was calculated as the percentage of total catchment area occupied by each LULC class. For each average temperature and total precipitation raster, local average temperature and total precipitation were summarized by within catchment averages. Subsequently, upstream accumulated values for each LULC and climate predictor were calculated using the NHDPlusV2 Catchment Attribute Allocation and Accumulation Tool (CA3TV2; http://www.horizon-systems.com/NHDPlus/NHDPlusV2_tools.php).

### 2.3 Model development, validation, and interpretation

We used random forests analysis (Breiman, 2001) to develop a baseline predictive model for the raw Chessie BIBI scores. Random forests are well suited for predicting stream condition scores because they can use continuous and categorical predictors, often perform exceptionally when compared with other methods (Fernández‐Delgado, Cernadas, Barro, & Amorim, 2014), are relatively insensitive to correlated covariates, and account for nonlinear relationships and complex interactions among predictors (Cutler et al., 2007). In total, 36 predictors were used (Table S1), including cumulative upstream watershed values for 15 natural predictors from StreamCat (Hill et al., 2016), 12 LULC categories representing baseline 2005 landscape conditions, seasonal average air temperature and total precipitation from PRISM, and dominant bioregion upstream of each stream reach given its importance in previous modeling efforts (Maloney, Smith, et al., 2018). The model was developed using the randomForest R package (Liaw & Wierner, 2002) with 1,000 trees and 19 variables randomly sampled at each split, which was optimal during tuning.

We divided the Chessie BIBI data into training (75%, *n* = 2,775) and test (25%, *n* = 925) datasets. Predictive performance was evaluated using model mean of squared residuals and percentage of explained variation in the training data. Since an important stakeholder goal is determining stream length in either Poor, Fair, or Good condition, we also tested model performance by how well it correctly classified these groups (i.e., percent correctly classified, PCC) and calculated the Kappa statistic (values <0.00 indicate poor agreement, 0.00–0.20 slight agreement, 0.21–0.40 fair agreement, 0.41–0.60 moderate agreement, 0.61–0.8 substantial agreement, and 0.81–1.00 almost perfect agreement; Landis & Koch, 1977). The test dataset was used to validate the model by calculating PCC and Kappa for the three‐category classification.

Several covariates were correlated in the training dataset (*r* > .70; Table S3); however, to avoid potentially missing an important climate covariate during prediction, all variables were retained regardless of correlation because random forest models are robust to multicollinearity. That said, correlated variables may affect variable importance plots and partial dependence plots (Molnar, 2019). Furthermore, land‐use and climate factors affect stream conditions through complex and multivariate interactions, which inhibits drawing inferences on functional relationships using partial dependence plots (Friedman, 2001). We used these plots only to interpret predictor strength and to evaluate relationship forms between predictor variables and raw stream condition scores and caution against overinterpreting these figures.

### 2.4 Model application and prediction

The Chesapeake Bay watershed has 83,637 catchments represented in the NHDplusV2; of these 10,666 were larger than 200 km^2^ and 2,199 had missing data and were not used here. We first used the model to predict scores and rating categories for the remaining 70,772 small stream segments to provide a 2000–2011 baseline estimate of watershed‐wide conditions. We then predicted watershed‐wide stream conditions under land‐use only projections, under climate‐only projections, and under combined land‐use and climate projections for each time period (2030, 2060, and 2090). For land‐use only stream condition predictions, we substituted the 2005 baseline LULC predictors with analogous data from each scenario (i.e., A1B, A2, B1, and B2) while keeping baseline climate (*n* = 12 scenarios). For climate only predictions, we substituted the 2005 baseline seasonal average temperature and total precipitation with analogous data from each projection (Lynch et al., 2016 and CMIP5 25th, 50th, and 75th percentiles) while keeping baseline LULC (*n* = 12 scenarios). For land‐use and climate combined predictions, we replaced both baseline LULC and climate predictors with analogous data from each climate projection and LULC projection for every possible combination (*n* = 48 scenarios). For the predicted 2005 baseline and each of the 72 future predicted stream conditions, we categorized predicted scores as either Poor, Fair, or Good using cutoffs of Smith et al. (2017).

To align with management goals, we linked predicted scores to the NHDPlusV2 Flowline dataset and calculated total stream kilometers and percentages predicted as Poor, Fair, and Good. We also calculated total kilometers and percentages that were predicted in improved or degraded condition categories for each scenario relative to baseline conditions. By using three categories to classify stream condition, there are three possibilities for a stream to be predicted to a degraded (Good to Fair, Good to Poor, and Fair to Poor) or improved (Poor to Fair, Poor to Good, and Fair to Good) condition. We calculated the total increase or decrease of stream kilometers and net change as the difference between these two values. An additive effect was calculated by summing net changes from associated land‐use and climate only scenarios and an interaction outcome was determined as the difference in magnitude between this effect and the net change for each combined scenario.

All analyses and figure generation were performed in the R Statistical Software Package, version 3.6.0 (R Development Core Team, 2019).

## 3 RESULTS

### 3.1 Model validation and interpretation

The baseline random forests model had a mean of squared residuals of 481.0 and explained 36.1% of variation in training data (*n* = 2,775). When classified into Poor, Fair, or Good conditions, the model correctly predicted 93.2% of Poor samples, 50.9% of Fair samples, and 94.4% of Good samples for an overall PCC of 84.9% and kappa statistic of 0.76 indicating substantial strength of agreement (Table 2). For test data (*n* = 925), 76.1% of Poor samples, 22.6% of Fair samples, and 74.8% of Good samples were correctly classified for an overall PCC of 60.0% and kappa of 0.39 (fair agreement).

**Table 2:** Confusion matrix for the training and test datasets when raw scores are categorized into Poor, Fair, or Good using cutoffs in Smith et al. (2017)

Predicted	Training data				Test data			
	Poor	Fair	Good	PCC	Poor	Fair	Good	PCC
Poor	972	58	13	93.2	242	22	54	76.1
Fair	152	295	132	50.9	117	61	92	22.6
Good	0	65	1,088	94.4	39	46	252	74.8
			Total	84.9			Total	60.0

Abbreviation: PCC, percent correctly classified.

The top two most important predictors in our baseline model were topographic wetness index and developed land cover (Figure S13) with predicted Chessie BIBI raw score showing a rapid decrease between a 700 and 800 topographic wetness index and between 0% and 20% developed land (Figure S14). Spring precipitation was within the top 10 most important variables; other climate variables were 13th or less most important (Figure S13). Predicted Chessie BIBI scores for all seasonal precipitation variables showed an initial increase and then the response either flattened (spring) or decreased (summer, winter, and fall, Figure S15). Predicted Chessie BIBI scores showed an initial flat response followed by a decrease for summer and spring temperatures, and initial increase response followed by no clear response to fall and winter temperatures (Figure S16).

### 3.2 Model predictions

Mean and median values of model covariates associated with the Chessie BIBI training dataset samples (*n* = 2,775) aligned closely to those in the NHDplusV2 dataset (*n* = 70,772); however, maximum values were larger for many covariates in the NHDPlusV2 dataset, especially for some land covers (e.g., mechanically disturbed land, croplands, and wetlands, Table S1). Thus, we have confidence the model was trained on data representing all but extreme high cases. For the 70,772 NHDPlus V2 reaches, 33.7% (23,825) were predicted as Good, 32.4% (22,934) as Fair, and 33.9% (24,013) as Poor condition. By stream length, of the 114,552 kilometers, the model predicted 37.5% (42,921 km) as Good, 29.0% (33,226 km) as Fair, and 33.5% (38,405 km) as Poor (Table S4).

### 3.3 Model predictions of stream kilometers in good condition

#### 3.3.1 Land‐use only scenario predictions

**Figure 2:**
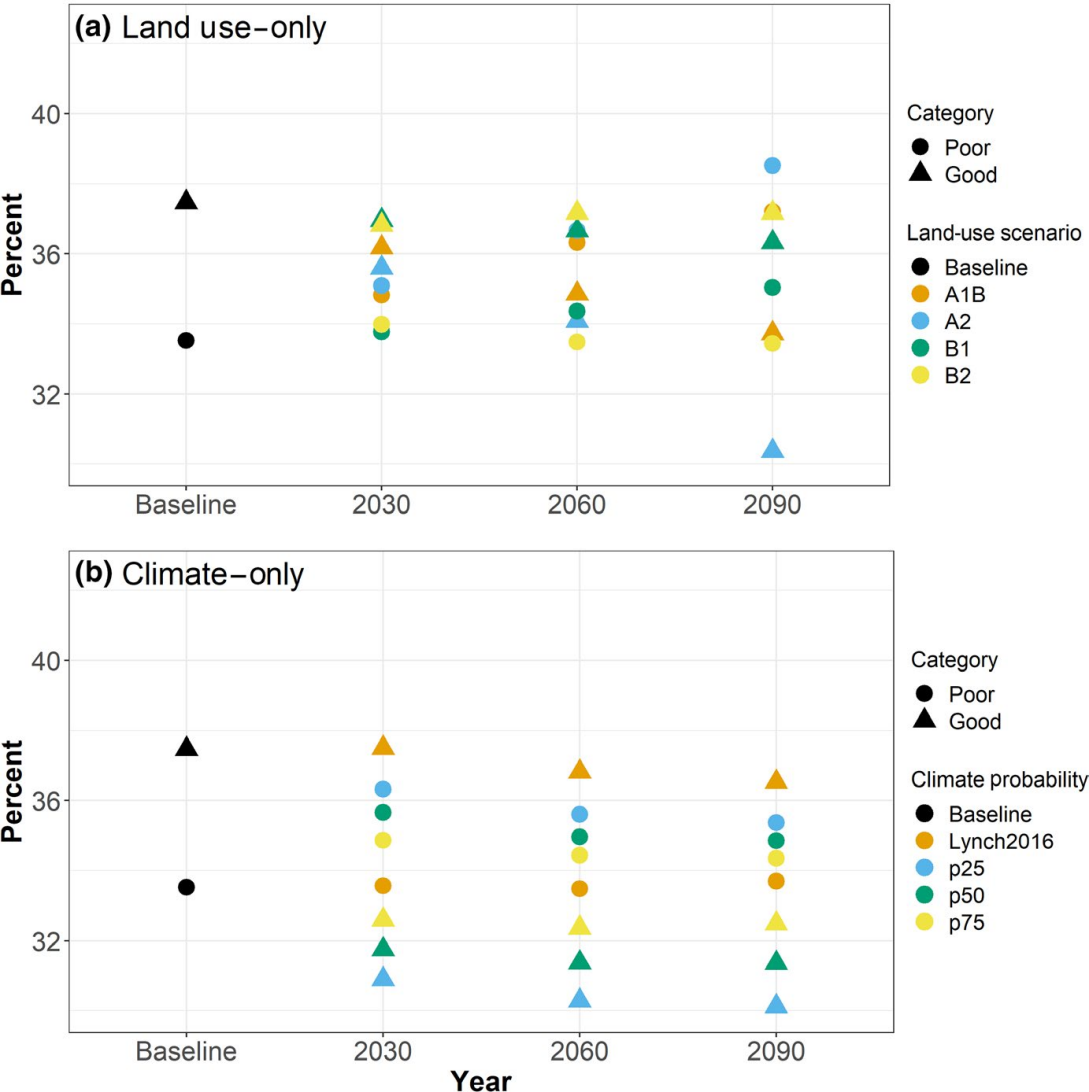
Predicted percentage of stream kilometers in Poor or Good condition under baseline and 2030, 2060, and 2090 land‐use projections (a) and climate projections (b). Fair condition not plotted for clarity, but results are in Table S4. Land‐use projections are described in Table 1. Climate projection abbreviations: Lynch2016 = Lynch et al. (2016) projections, p25 = 25th percentile of CMIP5 projections, p50 = 50th percentile from CMIP5 projections and p75 = 75th percentile form the CMIP5 projections. Stream kilometers and percentages by projected category for each land‐use and climate projection are in Table S4

Both economic growth (A1B and A2) scenarios showed a decrease in percentage of stream kilometers in Good condition for all three time periods with a maximum decrease by 2090 of 3.7% (4,286 km) under A1B and 7.1% (8,130 km) under A2 (Figure 2a; Table S4). The environmentally focused B1 scenario also predicted a decrease of streams in Good condition in each year, but by 2090 this decrease was only 1.1% (1,306 km). The most environmentally friendly scenario, B2, predicted a small decrease of streams in Good condition by 2030 (0.7%, 746 km) and by 2090 it predicted a 0.3% decrease (342 km).

#### 3.3.2 Climate only scenario predictions

When the 25th percentile of CMIP5 scenario (CMIP5 p25) was used, streams in Good condition decreased 6.6% (7,526 km) in 2030, 7.2% (8,220 km) in 2060, and 7.3% (8,406 km) by 2090 (Figure 2b; Table S4). The median CMIP5 scenario (CMIP5 p50) predicted a decrease of 5.7% (6,554 km) by 2030 and a decrease of 6.1% (6,991 km) by 2090. The 75th percentile CMIP5 (CMIP5 p75) scenario showed a decrease in stream kilometers in Good condition of 4.9% (5,573 km) by 2030 and a decrease of 5.0% (5,703 km) by 2090. The Lynch2016 climate scenarios showed an increase of stream kilometers in Good condition in 2030 (0.03%, 31 km) and decreases by 2060 (0.6%, 746 km) and 2090 (0.9%, 1,071 km).

#### 3.3.3 Combined land‐use and climate scenario predictions

**Figure 3:**
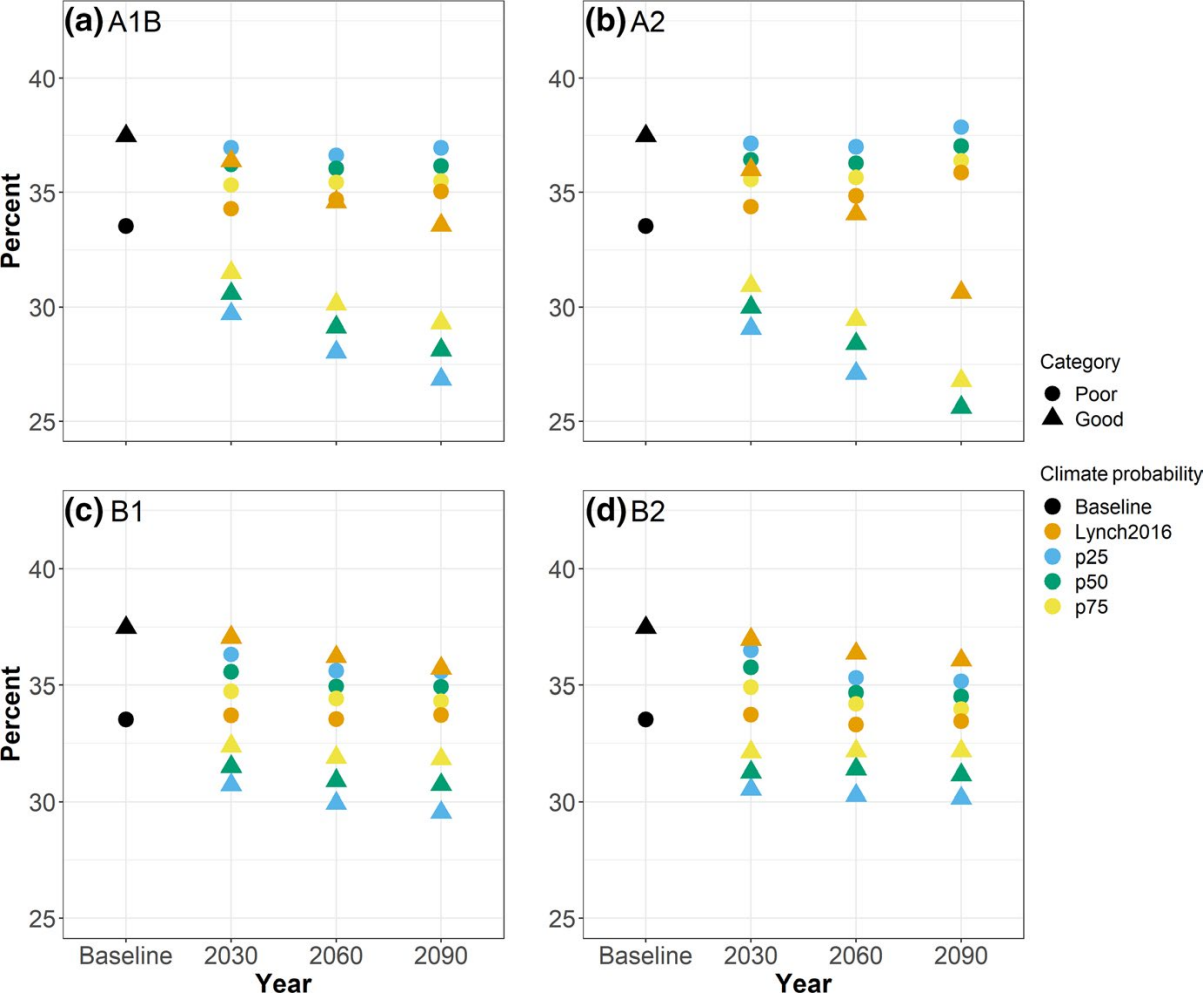
Predicted percentage of stream kilometers under Poor or Good condition for baseline and for each 2030, 2060, and 2090 land‐use and climate projection combination: A1B (a), A2 (b), B1 (c), B2 (d). Fair condition not plotted for clarity, but results are in Table S4. Climate projection abbreviations: Lynch2016 = Lynch et al., 2016 projections, p25 = 25th percentile of CMIP5 projections, p50 = 50th percentile from CMIP5 projections, and p75 = 75th percentile form the CMIP5 projections. Stream kilometers and percentages by projected category for each land‐use and climate projection are in Table S4

**Figure 4:**
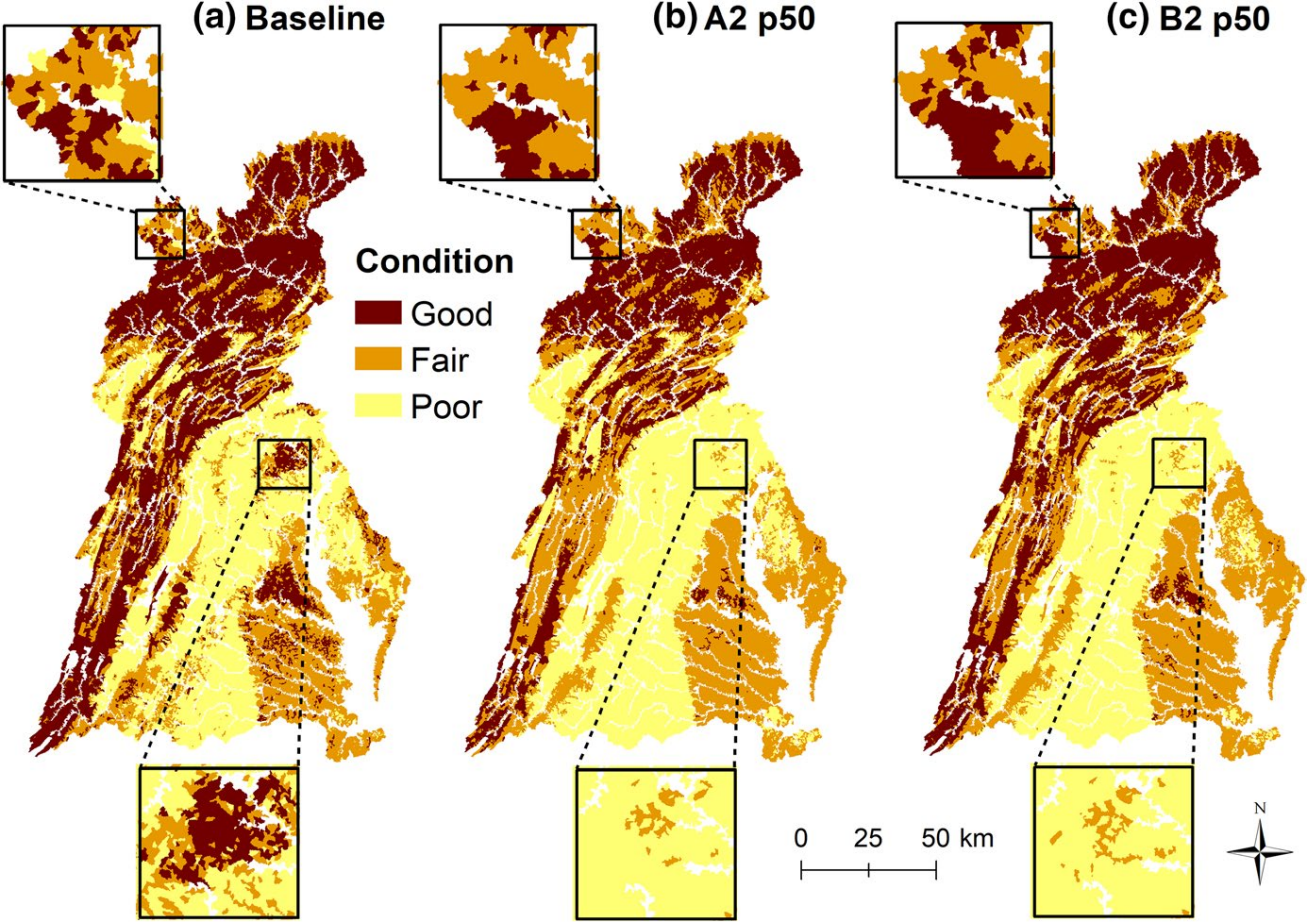
Maps showing NHDplusV2 catchments predicted to be in Poor, Fair, or Good condition under baseline conditions (a), A2 land‐use p50 CMIP5 climate 2090 projections (b), and B2 land‐use p50 CMIP5 climate 2090 projections (c). Upper focus area centers on region near Hornell, NY, lower focus area centers on region near York Pennsylvania, and Bel Air Maryland. Stream kilometers and percentages by projected category for each land‐use and climate projection are in Table S4

All combined land‐use and climate scenarios predicted a decrease in stream kilometers in Good condition; however, this decrease dampened with increasing percentile and the Lynch2016 scenarios (Figure 3; Table S4). The A2 climate scenarios generally predicted the largest decrease across all land‐use scenarios with the CMIP5 p25 climate scenario showing the largest decrease (13.4%, 15,344 km) in 2090; the A2 CMIP5 p50 and CMIP5 p75 combined scenarios predicted smaller decreases by 2090 (11.9% and 10.7%, respectively). Combined climate‐environmental sustainability land‐use scenarios predicted the smallest decrease in stream kilometers in good condition in 2030 (B1), 2060 (B2), and 2090 (B2). Predicted changes in stream conditions were spatially variable across the watershed with different directional changes occurring in different areas within the watershed within a single scenario (Figure 4).

### 3.4 Predicted stream kilometer change—2090

#### 3.4.1 Land‐use only predictions

By 2090, the A2 scenario predicted the largest change in percentage of stream kilometers to a degraded condition (12.6%), followed by A1B (7.9%), B1 (4.0%), and then B2 (3.2%, Table 3). The opposite pattern was observed for percentage of stream kilometers predicted in an improved condition (B2 > B1 > A1B > A2) resulting in a net decrease in stream condition being highest for A2 (11.5%), followed by A1B (7.0%), then B1 (2.5%) and B2 (0.2%).

**Table 3:** Percentage of stream kilometers from NHDPlusV2 separated by predicted change in stream conditions for each land‐use and climate scenario

Land use	Climate model	Year	Percent													
			Fair/Fair	Fair/Good	Fair/Poor	Good/Fair	Good/Good	Good/Poor	Poor/Fair	Poor/Good	Poor/Poor	Total Incr.	Total Decr.	Net Change	Add. effect	Interaction outcome
Land‐use only projections																
A1B	Baseline	2030	26.2	0.4	1.7	1.7	34.9	0.0	0.4	0.0	32.3	0.8	3.4	−2.5	na	na
A1B	Baseline	2060	24.9	0.4	3.1	2.9	33.6	0.1	0.4	0.0	32.3	0.9	6.0	−5.2	na	na
A1B	Baseline	2090	24.1	0.3	3.9	3.8	32.6	0.2	0.5	0.0	32.2	0.8	7.9	−7.0	na	na
A2	Baseline	2030	26.0	0.4	2.0	2.2	34.4	0.0	0.4	0.0	32.3	0.8	4.2	−3.4	na	na
A2	Baseline	2060	24.4	0.5	3.5	3.7	32.8	0.1	0.5	0.0	32.2	1.0	7.3	−6.3	na	na
A2	Baseline	2090	22.7	0.4	5.2	7.1	29.3	0.3	0.6	0.0	32.1	1.0	12.6	−11.5	na	na
B1	Baseline	2030	26.7	0.7	1.0	1.2	35.4	0.0	0.7	0.0	32.0	1.4	2.1	−0.7	na	na
B1	Baseline	2060	26.1	0.7	1.5	1.5	35.1	0.0	0.8	0.0	32.0	1.5	3.1	−1.6	na	na
B1	Baseline	2090	25.5	0.7	2.1	1.8	34.8	0.1	0.7	0.0	32.0	1.4	4.0	−2.5	na	na
B2	Baseline	2030	26.7	0.6	1.0	1.2	35.4	0.0	0.6	0.0	32.1	1.2	2.2	−1.1	na	na
B2	Baseline	2060	25.7	1.3	1.3	1.6	35.0	0.0	1.4	0.0	31.4	2.7	2.9	−0.2	na	na
B2	Baseline	2090	25.4	1.5	1.4	1.7	34.8	0.0	1.5	0.0	31.2	3.0	3.2	−0.2	na	na
Climate only projections																
Baseline	CMIP5 p25	2030	23.5	0.6	4.3	6.4	29.6	0.6	2.2	0.0	30.6	2.8	11.3	−8.5	na	na
Baseline	CMIP5 p25	2060	23.5	0.6	4.2	6.9	29.0	0.8	2.9	0.0	29.8	3.5	11.8	−8.3	na	na
Baseline	CMIP5 p25	2090	23.5	0.7	4.2	7.0	28.8	0.8	3.2	0.0	29.6	3.8	12.0	−8.2	na	na
Baseline	CMIP5 p50	2030	23.6	0.7	4.0	5.8	30.3	0.5	2.4	0.0	30.3	3.2	10.3	−7.1	na	na
Baseline	CMIP5 p50	2060	23.6	0.8	3.9	6.1	29.8	0.7	3.1	0.0	29.6	4.0	10.7	−6.7	na	na
Baseline	CMIP5 p50	2090	23.5	0.9	4.0	6.2	29.7	0.7	3.3	0.0	29.4	4.2	10.8	−6.6	na	na
Baseline	CMIP5 p75	2030	23.7	1.0	3.7	5.2	30.9	0.5	2.8	0.0	29.9	3.8	9.4	−5.6	na	na
Baseline	CMIP5 p75	2060	23.6	1.0	3.7	5.5	30.6	0.6	3.4	0.0	29.4	4.4	9.7	−5.3	na	na
Baseline	CMIP5 p75	2090	23.5	1.0	3.8	5.4	30.7	0.5	3.5	0.0	29.3	4.5	9.7	−5.2	na	na
Baseline	Lynch2016	2030	23.5	2.2	2.6	2.1	34.4	0.1	2.6	0.0	30.2	4.8	4.8	0.0	na	na
Baseline	Lynch2016	2060	22.9	2.6	2.8	3.0	33.3	0.2	3.1	0.0	29.7	5.7	6.1	−0.4	na	na
Baseline	Lynch2016	2090	22.5	2.8	3.1	3.4	32.9	0.3	3.2	0.1	29.5	6.0	6.8	−0.9	na	na
Combined land‐use and climate projections																
A1B	CMIP5 p25	2030	23.0	0.6	4.8	7.5	28.4	0.7	2.1	0.0	30.6	2.7	13.0	−10.3	−11.1	Less degraded
A1B	CMIP5 p25	2060	22.8	0.6	4.9	9.0	26.8	0.9	2.8	0.0	29.9	3.4	14.8	−11.4	−13.5	Less degraded
A1B	CMIP5 p25	2090	22.5	0.6	5.2	10.0	25.6	1.0	2.9	0.0	29.9	3.5	16.2	−12.7	−15.2	Less degraded
A1B	CMIP5 p50	2030	23.1	0.8	4.5	6.9	29.1	0.6	2.4	0.0	30.3	3.2	11.9	−8.8	−9.7	Less degraded
A1B	CMIP5 p50	2060	23.0	0.8	4.6	8.2	27.6	0.8	2.9	0.0	29.9	3.7	13.5	−9.8	−11.9	Less degraded
A1B	CMIP5 p50	2090	22.8	0.8	4.7	9.1	26.6	0.9	3.0	0.0	29.7	3.9	14.7	−10.8	−13.7	Less degraded
A1B	CMIP5 p75	2030	23.3	1.0	4.0	6.3	29.8	0.6	2.8	0.0	29.9	3.8	10.9	−7.0	−8.1	Less degraded
A1B	CMIP5 p75	2060	23.0	1.0	4.3	7.5	28.4	0.7	3.1	0.0	29.6	4.1	12.5	−8.3	−10.5	Less degraded
A1B	CMIP5 p75	2090	22.9	1.0	4.4	8.2	27.6	0.8	3.2	0.0	29.5	4.3	13.4	−9.2	−12.2	Less degraded
A1B	Lynch2016	2030	23.2	2.1	3.1	3.0	33.5	0.1	2.5	0.0	30.3	4.5	6.2	−1.7	−2.5	Less degraded
A1B	Lynch2016	2060	22.4	2.3	3.6	4.7	31.5	0.5	2.9	0.0	29.8	5.2	8.7	−3.5	−5.6	Less degraded
A1B	Lynch2016	2090	22.0	2.4	4.0	5.7	30.3	0.6	3.1	0.1	29.6	5.5	10.3	−4.8	−7.9	Less degraded
A2	CMIP5 p25	2030	22.9	0.5	4.9	8.0	27.9	0.7	2.1	0.0	30.6	2.7	13.7	−11.0	−11.9	Less degraded
A2	CMIP5 p25	2060	22.5	0.6	5.3	9.8	25.9	0.9	2.9	0.0	29.9	3.4	16.0	−12.6	−14.6	Less degraded
A2	CMIP5 p25	2090	21.8	0.5	6.0	12.5	23.0	1.1	2.9	0.0	29.9	3.4	19.6	−16.2	−19.7	Less degraded
A2	CMIP5 p50	2030	23.1	0.7	4.6	7.3	28.6	0.7	2.4	0.0	30.3	3.1	12.5	−9.5	−10.5	Less degraded
A2	CMIP5 p50	2060	22.7	0.8	4.9	8.8	27.0	0.8	3.0	0.0	29.7	3.8	14.5	−10.7	−13.0	Less degraded
A2	CMIP5 p50	2090	22.2	0.7	5.5	11.3	24.3	1.0	3.0	0.0	29.7	3.7	17.8	−14.0	−18.1	Less degraded
A2	CMIP5 p75	2030	23.3	0.9	4.2	6.7	29.3	0.6	2.8	0.0	30.0	3.7	11.5	−7.8	−8.9	Less degraded
A2	CMIP5 p75	2060	22.8	1.0	4.5	8.1	27.7	0.8	3.2	0.0	29.6	4.2	13.4	−9.2	−11.6	Less degraded
A2	CMIP5 p75	2090	22.5	0.8	5.1	10.4	25.3	0.9	3.1	0.0	29.6	4.0	16.3	−12.4	−16.7	Less degraded
A2	Lynch2016	2030	23.3	1.9	3.1	3.2	33.3	0.2	2.4	0.0	30.3	4.3	6.4	−2.1	−3.3	Less degraded
A2	Lynch2016	2060	22.4	2.2	3.7	5.1	31.1	0.5	2.9	0.0	29.8	5.1	9.3	−4.2	−6.7	Less degraded
A2	Lynch2016	2090	22.0	1.8	4.6	7.8	28.1	0.7	3.0	0.1	29.7	4.8	13.1	−8.3	−12.4	Less degraded
B1	CMIP5 p25	2030	23.1	0.7	4.5	6.7	29.3	0.6	2.4	0.0	30.3	3.2	11.8	−8.7	−9.3	Less degraded
B1	CMIP5 p25	2060	23.0	0.8	4.5	7.4	28.4	0.8	3.3	0.0	29.5	4.1	12.7	−8.6	−9.9	Less degraded
B1	CMIP5 p25	2090	23.0	0.8	4.5	7.7	28.0	0.9	3.4	0.0	29.3	4.2	13.1	−8.9	−10.7	Less degraded
B1	CMIP5 p50	2030	23.3	0.9	4.2	6.2	29.9	0.6	2.7	0.0	30.0	3.6	10.9	−7.3	−7.9	Less degraded
B1	CMIP5 p50	2060	23.2	1.0	4.1	6.7	29.2	0.7	3.4	0.0	29.3	4.4	11.5	−7.1	−8.3	Less degraded
B1	CMIP5 p50	2090	23.1	1.0	4.2	6.9	29.0	0.8	3.6	0.0	29.2	4.6	11.8	−7.2	−9.1	Less degraded
B1	CMIP5 p75	2030	23.4	1.1	3.8	5.6	30.5	0.5	3.1	0.0	29.6	4.3	10.0	−5.7	−6.3	Less degraded
B1	CMIP5 p75	2060	23.3	1.2	3.9	6.0	30.0	0.6	3.6	0.0	29.1	4.8	10.5	−5.7	−6.9	Less degraded
B1	CMIP5 p75	2090	23.2	1.2	4.0	6.1	29.9	0.6	3.8	0.0	28.9	5.0	10.7	−5.7	−7.7	Less degraded
B1	Lynch2016	2030	23.2	2.4	2.8	2.7	33.8	0.1	2.6	0.0	30.1	5.0	5.5	−0.5	−0.7	Less degraded
B1	Lynch2016	2060	22.5	2.7	3.1	3.7	32.6	0.3	3.3	0.1	29.4	6.1	7.1	−1.0	−1.9	Less degraded
B1	Lynch2016	2090	22.1	2.9	3.4	4.3	32.0	0.4	3.5	0.1	29.2	6.4	8.0	−1.6	−3.4	Less degraded
B2	CMIP5 p25	2030	23.2	0.7	4.5	6.8	29.2	0.7	2.2	0.0	30.5	2.9	11.9	−9.0	−9.6	Less degraded
B2	CMIP5 p25	2060	23.0	1.0	4.3	7.2	28.6	0.9	3.4	0.0	29.3	4.5	12.4	−7.9	−8.5	Less degraded
B2	CMIP5 p25	2090	22.9	1.1	4.3	7.3	28.4	0.9	3.6	0.0	29.1	4.7	12.6	−7.9	−8.4	Less degraded
B2	CMIP5 p50	2030	23.4	0.8	4.1	6.3	29.7	0.6	2.6	0.0	30.2	3.3	11.0	−7.7	−8.2	Less degraded
B2	CMIP5 p50	2060	23.0	1.3	4.0	6.5	29.3	0.8	3.6	0.0	29.1	5.0	11.3	−6.3	−6.9	Less degraded
B2	CMIP5 p50	2090	22.9	1.3	4.1	6.8	29.1	0.8	3.9	0.0	28.9	5.2	11.6	−6.4	−6.8	Less degraded
B2	CMIP5 p75	2030	23.5	1.0	3.8	5.7	30.3	0.5	3.0	0.0	29.8	4.0	10.0	−6.1	−6.7	Less degraded
B2	CMIP5 p75	2060	23.0	1.5	3.8	6.0	29.9	0.7	3.8	0.0	28.9	5.3	10.5	−5.2	−5.6	Less degraded
B2	CMIP5 p75	2090	22.9	1.5	3.9	6.1	29.9	0.6	4.1	0.0	28.6	5.7	10.6	−5.0	−5.4	Less degraded
B2	Lynch2016	2030	23.4	2.2	2.7	2.7	33.9	0.1	2.6	0.0	30.2	4.8	5.4	−0.6	−1.0	Less degraded
B2	Lynch2016	2060	22.4	3.0	3.0	3.8	32.5	0.3	3.4	0.1	29.2	6.5	7.1	−0.6	−0.6	Equal
B2	Lynch2016	2090	21.9	3.2	3.3	4.3	31.9	0.4	3.6	0.1	29.0	6.9	7.9	−1.0	−1.1	Less degraded

Note

Coding convention under Percent: first category indicates the baseline predictions and the second category indicates the time period of future prediction. For example, Good/Good indicates the percentage of stream kilometers predicted in good condition under both baseline and 2090, Good/Fair indicates the percentage of stream kilometers with predicted good conditions under baseline conditions but fair conditions in 2090. Total Incr. = percentage of total stream kilometers with predicted improved categorical condition, Total Decr. = percentage of total stream kilometers with degraded categorical condition, Net Change = Total Incr. − Total Decr., Add. Effect = Additive effects calculated as the sum of associated land‐use only and climate only Net Changes, and Interaction Outcome relates how overall watershed condition from the combined scenarios related to the additive effect of individual scenarios. Land‐use projection abbreviations listed in Table 1. Values for stream lengths are in Table S5.

#### 3.4.2 Climate only predictions

By 2090, CMIP5 p25 predicted the largest amount of stream kilometers in a degraded condition, followed by CMIP5 p50 and CMIP5 p75, and then Lynch2016 (Table 3). The opposite pattern was observed for stream kilometers predicted in improved conditions (Lynch2016 > CMIP5 p75 > CMIP5 p50 > CMIP5 p25), resulting in net change in decreased conditions being highest for CMIP5 p25 and lowest for the Lynch2016 scenario.

#### 3.4.3 Combined land‐use and climate predictions

For all climate scenarios, by 2090, A2 combined scenarios predicted the most streams in degraded conditions followed by A1B, B1, and then B2; an opposite pattern was predicted for streams in improved conditions (B2 > B1 > A1B > A2, Table 3). By 2090, the largest percentage of streams predicted to degraded conditions was under A2 CMIP5 p25 (19.6%), while the smallest percentage was under B2 Lynch2016 scenario (7.9%); conversely A2 CMIP5 p25 in 2090 predicted the smallest percentage of streams in improved conditions (3.4%) and B2 Lynch2016 predicted the largest percentage of streams to improved conditions (6.9%). By 2090, all combined land‐use and climate scenarios predicted a net increase in stream kilometers in degraded conditions (16.2% under A2 CMIP p25 to 1.0% under B2 Lynch2016).

By 2090, the additive effect of individual scenarios for all combined land‐use climate scenarios predicted a net increase in stream kilometers in degraded conditions (Table 3). These additive effects were all larger than analogous net changes, indicating combining climate and land‐use predicted a less degraded condition across the watershed (Table 3; Table S5).

### 3.5 Spatial patterns in stream condition changes—2090

Land‐use only scenarios showed a wide range in how predicted changes in stream condition spatially organized across the watershed. Scenario A2 predicted widespread degradation in stream conditions, whereas B2 predicted widespread improvements in stream conditions by 2090; A1B and B1 scenarios predicted spatial patterns in between A2 and B2 (Figure S17). All climate only scenarios predicted improved stream conditions for northern and mid‐eastern portions of the watershed; the number of improved streams increased with CMIP5 quartiles and was highest for the Lynch2016 scenario (Figure S18). All climate only scenarios predicted degraded stream conditions in south central portions of the watershed.

**Figure 5:**
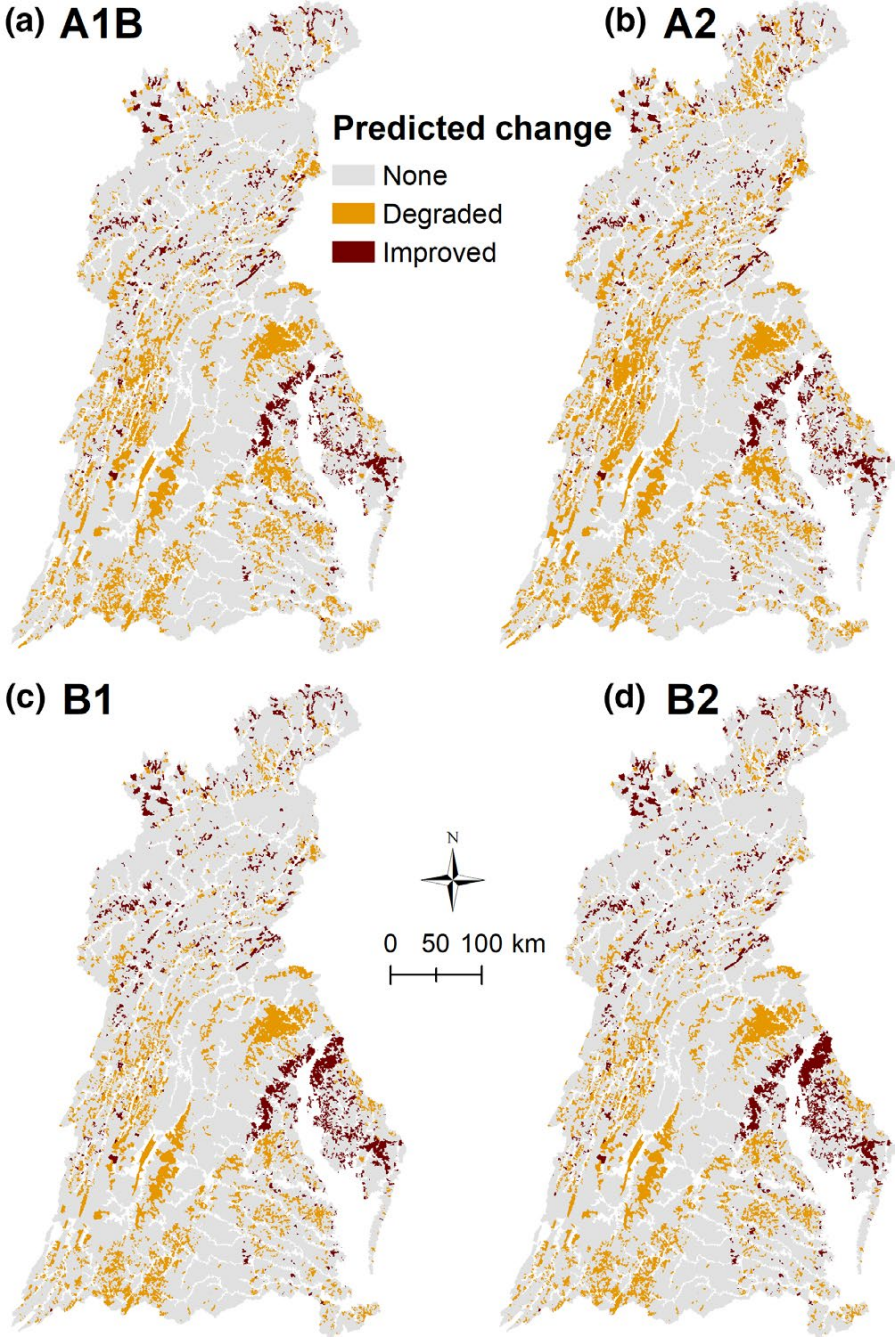
Maps showing NHDPlusV2 catchments with predicted change in stream conditions in 2090 under CMIP5 p50 climate projections and A1B (a), A2 (b), B1 (c), and B2 (d) land‐use projections. Coding convention: “None” = a stream reach was predicted in the same condition in baseline and 2090 conditions (e.g., Poor to Poor), “Degraded” = a stream reach was predicted at a lower category in 2090 than baseline period (e.g., Good to Fair), and “Improved” = a stream reach was predicted at a higher category in 2090 than baseline period (e.g., Fair to Good). Similar maps for 2030 under CMIP5 p50 climate projections and 2060 under CMIP5 p50 climate projections are in Figures S19 and S20. Stream kilometers and percentages by predicted stream condition change are in Table S5 and Table 3

For all combined scenarios, by 2090, improved stream conditions were predicted more often in northern and far eastern portions (Delmarva Peninsula) of the watershed and degraded stream conditions were predicted more often in central and southern portions (Figure 5; see Figures S19 and S20 for 2030 and 2060 maps). A1B and A2 predicted more widespread degradation in stream conditions than B1 and B2.

## 4 DISCUSSION

Our results suggest a highly variable response of stream conditions in the Chesapeake Bay watershed to future potential land‐use and climatic changes. Such variability was expected because land‐use and climate scenarios are not exact predictions of future conditions but rather tools that can be used to examine various possible futures and associated uncertainties (Van Vuuren & Carter, 2014). We incorporated multiple future land‐use and climate scenarios to capture the range of potential future stream conditions as well as highlight projection uncertainties. Although the A2 land‐use scenario that projects a global population of 13 billion by 2100 is slightly outdated (Van Vuuren & Carter, 2014), we incorporated it as a worst‐case scenario. Furthermore, although global population may not increase to such a degree, such regional population increases may occur. The two million projected increase in population for the Chesapeake by 2030 (~150,000/year, 0.9%, Chesapeake Bay Program, 2017a), if continued through 2100, would add ~12.5 million people to the watershed, a proportional increase near what is predicted in A2 (~3.6 million/year, 1.1%). Global climate models also are highly variable and to reduce the need to independently test many global climate models we used quartiles of projections from the CMIP5 program. Weighting all scenarios equally, by 2090 our results suggest that changes to watershed‐wide stream conditions could range from a 16.2% (A2 CMIP5 p25) to 1.0% (B2 Lynch2016) degradation of stream kilometers.

### 4.1 Model interpretation and prediction

Model accuracy is an important component of any prediction study. Our accuracy diagnostics are similar to prediction‐based ordinal stream condition studies performed previously within the study region (e.g., Maloney, Weller, Russell, & Hothorn, 2009) but slightly less than those from binomial stream condition studies both inside and outside the region (Hill et al., 2017; Maloney, Smith, et al., 2018). Overall, this suggests our model is suitable for use in both prediction of current conditions of unsampled areas and under future land‐use and climate conditions. However, another issue is temporal consistency of prediction‐based models. We are unaware of a study that has addressed how accurately models predict a response in different time periods in a single region, likely a result of limited long‐term, large‐scale data. Here, given the 6‐year sampling cycle, we were able to evaluate two periods: 2000–2005 and 2006–2011. Thus, to test temporal consistency of predictive landscape models, we built separate random forests models for (a) 2000–2005 Chessie BIBI data with 2000–2005 PRISM precipitation and temperature data and 2001 NLCD data (*n* = 1,649) and (b) 2006–2011 Chessie BIBI data with 2006–2011 PRISM precipitation and temperature data and 2006 NLCD data (*n* = 2,698). Models for both periods performed similarly (training data PCCs = 83.3 and 85.6, respectively; test data PCCs = 55.0 and 65.3, respectively; Table S6). Although only two periods, these results suggest robustness in temporal accuracy; however, longer term data are needed to more rigorously test temporal consistency.

Our prediction of 66.1% of small stream reaches in the Chesapeake Bay watershed currently in Fair or Good condition is slightly higher than the 64.0% predicted in Maloney, Smith, et al. (2018). Close agreement was surprising given the latter used a finer base layer (1:24,000), different covariates, and Chessie BIBI data from 2004 to 2008. Buchanan et al. (2018) more recently estimated streams in approximately 60.0% of the basin's area were in Fair or better condition using a combination of Maloney, Smith, et al. (2018) model results and monitoring data for 2006–2011. Together, all three provide support that the baseline number of stream reaches in Fair or better condition in the watershed was between 60.0% and 66.1% in the 2000–2011 period. Here, we expanded upon these previous studies by estimating 66.5% of stream kilometers in Fair/Good conditions in the 2000–2011 period, a measure more directly aligned with the stated management goal.

Land‐use and climate covariates were important drivers of stream condition. The importance of developed cover supports previous studies (Maloney, Schmid, & Weller, 2012; Roy, Rosemond, Leigh, Paul, & Wallace, 2003; Walsh et al., 2005) as does the importance of agriculture cover (Hay_Pasture and Cropland; Allan, Erickson, & Fay, 1997) and forest cover (Maloney, Smith, et al., 2018). The high importance of upstream developed land as a driver was expected given it alters streams through modified flow and thermal regimes as well as increases in sediments, toxic contaminants, and other stressors (Walsh et al., 2005)—all of which negatively affect benthic macroinvertebrates. Positive trends between precipitation and Chessie BIBI scores suggest that stream conditions are higher in more humid areas. However, trends between temperature and Chessie BIBI scores suggest there may be an upper limit to increased temperature on stream condition. Many streams in the Chesapeake are cold water, which could be more sensitive than warmer systems to increasing temperature (Heino, Virkkala, & Toivonen, 2009). Together, the strong effects of land‐use and climate underscore the potential sensitivity of streams to changes in these features.

The importance of topographic wetness index and the rapid decrease in predicted Chessie BIBI suggests valleys and areas with lower upstream catchment slopes had a lower Chessie BIBI score, and thus more degraded conditions. Larger topographic wetness index values, indicative of valley bottoms, were in the southeastern portion of the study area (Figure S21), largely the Southeastern Plains and Middle Atlantic Coastal Plains bioregions (Figure 1). In a contiguous US study, Hill et al. (2017) reported topographic wetness index was an important driver of stream conditions in the Coastal Plains region, which aligned with these two Chesapeake Bay watershed plains bioregions. This area also had disproportionate amounts of developed and agricultural lands (Figure S21); thus, there could be some interaction between topographic wetness index and anthropogenic stress occurring (i.e., these areas are more supportive of intensive agriculture and urban or suburban development).

### 4.2 Land‐use and climate only predictions

#### 4.2.1 Land‐use only

Results from land‐use only predictions suggest net changes in stream condition ranged from degradation in 11.5% to 0.2% of stream kilometers. A net increase in streams in degraded condition from 2030 to 2090 was predicted for both economic development scenarios (A1B and A2), and although at much smaller changes, also for the more environmentally focused B1 and B2 scenarios. Developed land more than doubled for A1B and A2 by 2090 (Table S7), which likely drove the degradation in stream conditions given its negative functional response with Chessie BIBI (Figure S14). B1 and B2 were projected to have a smaller increase in developed land than A1B or A2, which is a likely reason behind the relatively small increases in streams in degraded condition. An important concern for predicting future conditions is that distributions of data in the predictive model cover the range in projected years. Our data for developed land in the training (Table S1) and baseline scenarios (Figure S22) overlapped all future scenario distributions, so the model was suitable to predict across the range of developed land in all scenarios.

#### 4.2.2 Climate only

Except for the Lynch2016 2030 scenario, all climate only scenarios projected a net degradation in stream conditions for the Chesapeake Bay watershed; however, the magnitude of degradation decreased with increasing percentiles of the downscaled climate change models (CMIP5 scenarios) and was smallest for the watershed‐wide uniform scenarios (Lynch2016). Thus, the scenario analyses suggest increasing precipitation may mollify the effects of increasing temperature in the future. The distribution of total precipitation in the training dataset overlapped those for baseline 2005 data, but baseline data were slightly higher than historical and all projections (Table S1, Figure S23). The distribution of future temperature projections was shifted above the upper limits of the baseline data for all projections, especially by 2090 (Table S1, Figure S24). Thus, due to potentially novel climate conditions in the future, our model may not effectively predict the effects of lower precipitation projections and higher temperature projections especially for the later year (2090) and higher quartile (p75) scenarios.

We observed an effect of downscaling climate models on stream condition predictions with the regionally uniform climate scenario (Lynch et al., 2016) showing a smaller net proportion of degraded stream kilometers than downscaled CMIP5 scenarios. Lynch2016 projected slightly higher precipitation levels than the CMIP5 scenarios but projected changes in temperature within the range of the CMIP5s (Figures S7–S12). Therefore, it is likely that local variation afforded by downscaled CMIP5 scenarios was behind these differences and highlights an importance of including downscaled data when appropriate.

#### 4.2.3 Land‐use only versus climate only scenario predictions

By 2090, both land‐use only and climate only predictions of watershed‐wide stream conditions ranged from a small to large net increase in degraded condition. Furthermore, possible future land‐use under economic development scenarios had an amplifying pattern with increasing degraded condition in out years, whereas the effects of climate futures were relatively consistent across out years. This pattern was most evident with the A2 scenario as it projected half or less the net change than CMIP5 scenarios in 2030 but a higher net change by 2090. The lack of a climatic future trend may be a result of the distribution of baseline data, especially temperature, not encompassing futures. Also, developed land and cropland under A2 and precipitation and air temperature were projected to change dramatically by 2090. However, developed land was a more important covariate in the model than any climate variable (Figure S13) and exhibited a strong negative functional relationship with the Chessie BIBI, while precipitation and temperature did not (Figures S14–S16). Environmentally sustainable scenarios predicted absolute changes below the range of changes under CMIP5 climate only scenarios, but similar to projections under Lynch2016. The magnitude of developed land cover changes under scenarios B1 and B2 was small and forest cover increased under B2 in 2060 and 2090, which resulted in fewer streams predicted to a degraded condition compared to those predicted to an improved condition in out years for these scenarios.

### 4.3 Combined land‐use and climate scenario predictions

The high variability observed with the individual scenarios carried over to the combined scenario analyses which by 2090 also predicted watershed‐wide stream conditions ranging from large increases in degraded conditions (combined economic development scenarios) to small increases in degraded conditions (combined Lynch2016 and B1 and B2 scenarios). Previous studies have documented variable effects of climate and land‐use change on individual taxa (Krause et al., 2019; Mantyka‐Pringle, Martin, Moffatt, Linke, & Rhodes, 2014). However, these studies all focused on individual taxa or individual metrics and we are unaware of a previous study that has examined an index of stream condition to global change. Previous studies are also largely limited in the number of land‐use and climate scenarios tested. Here, we showed how including a suite of land‐use and climate scenarios that bracket likely future conditions predicted different future stream conditions. Documenting this uncertainty is important not only to improve our basic understanding of how global change may affect stream conditions but also to aid in restoration and conservation management efforts.

While patterns from individual scenarios carried over to combined scenarios, all combined scenarios showed an interactive effect of land‐use and climate change on watershed‐wide stream conditions. Nearly all combined scenarios revealed a response in stream condition that was different than the additive effects of land‐use and climate. By 2090, all combined climate and land‐use scenarios predicted a mediating effect with interaction outcomes of less degraded watershed‐wide conditions. Other research has suggested an interactive effect of land‐use and climate change on stream benthic macroinvertebrates (Kuemmerlen et al., 2015; Li, Zhang, Guo, Gao, & Wang, 2018). These results emphasize the need to acknowledge the interacting effects of land‐use and climate when forecasting future environmental conditions and reflect a need to further investigate how these interactions may differ among areas with distinct abiotic conditions.

#### 4.3.1 Spatial patterns

Displayed graphically, predictions from combined land‐use and climate scenarios identify geographic areas where climate change may mediate effects of land‐use in the Chesapeake Bay watershed (e.g., north and middle eastern part of basin) and other areas where such effects of climate may magnify land‐use effects (e.g., high development scenario A2 in 2090, Figure 5). A spatial understanding of such interactive effects could be used by researchers, managers, and policy makers to minimize risk or take advantage of opportunities afforded by these interactions (Oliver & Moorecraft, 2014). For example, areas where climate has a larger mediating effect could be prioritized for restoration because climate may help amplify restoration effects, whereas, areas predicted to experience a magnified effect of climate change may warrant strong intervention to stymie possible future loss and to realize restoration goals.

### 4.4 Limitations of models

We acknowledge we were limited on available land‐use and climate covariates, which may have affected the predictive baseline model and thus future predictions. Both were at coarse resolutions (land‐use = 250 m, climate = 800 m), which may not encapsulate changes in land cover and climate variables that could be seen at finer scales. To facilitate summation of these factors with the NHDPlusV2 dataset, we also resampled each to 30 m. Doing so enabled better summation for smaller catchments (e.g., enabled raster centroids to be placed within catchments); however, we acknowledge such a procedure generated finer scaled rasters but not finer scaled representations of land cover.

We also acknowledge limitations in defining seasons by months defined by water years and there are other aggregation options (e.g., meteorological seasons). Any such aggregation is an oversimplification of seasons in this area, which are likely not uniform but rather transitional based on spatial (e.g., latitude) and landscape (e.g., elevation) factors. Defining seasons that incorporate these factors would likely improve seasonal inference but is beyond the scope of the current study.

We also limited our study to using four SRES land‐use projections (A1B, A2, B1, and B2) because these were available for our study area. Representative Concentration Pathways (RCP) 2.6, 4.5, 6.0, and 8.5 and shared socioeconomic pathways (SSP) projections exist but are currently not available for the Chesapeake Bay watershed at similar spatial and thematic resolution. The development of spatially explicit scenarios that represent both climate and land‐use is a highly time‐consuming process (Van Vuuren & Carter, 2014); therefore, these data may not be available for this watershed for several years. To facilitate climate and land‐use change studies, Van Vuuren and Carter (2014) reconciled the SRES with the newer RCP and SSP scenarios, suggesting A2 ≈ RCP 8.5 and SSP3, B2, or A1B ≈ RCP 6.0 and SSP2, and B1 ≈ RCP 4.5 and SSP1. Thus, our results should be robust to these more recent scenarios. We also note that land‐use projections are based on existing information at the time of model development, and thus do not contain technological advances that lead to unexpected land‐use changes, such as the development of shale oil and gas development that began in the northern portion of the watershed in 2010 (Maloney, Young, et al., 2018).

Optimally long‐term biological, land‐use, and climate datasets would be available to more definitively tease out effects of land‐use and climate changes on biological process (Northrup et al., 2019) and distinguish them from other factors that change biodiversity and abundance (e.g., diseases, introductions, extirpations, and genetic drift). In the absence of such data, models are developed under a baseline scenario where biological data are sufficient, and then, this model is used to predict biological conditions under projected future conditions. Doing so we assume contemporary relationships will hold in the future; however, we acknowledge this may not be the case, particularly when novel climate and land‐use combinations may exist in the future without current correlates.

For our climate change predictions of stream condition, we used surrogates for instream biologically relevant measures of water temperature and streamflow, which are an oversimplification of the ways in which changing climatic conditions will affect stream thermal and flow regimes (Knouft & Ficklin, 2017; Morrill, Bales, & Conklin, 2005). Using more biologically relevant metrics would likely improve model strength and our mechanistic understanding of climate effects on stream condition (Kuemmerlen et al., 2015; Merriam, Petty, & Clingerman, 2019; Pyne & Poff, 2017). Unfortunately, such models for headwaters streams are not available for the entire watershed.

### 4.5 Management implications

Managers are faced with protecting, conserving, and restoring biological populations and associated ecosystems under continually changing land‐use, water demands, and climate conditions. Regionally, our results are important considerations in attaining and maintaining the 10% stream improvement goal for the watershed (Chesapeake Bay Program, 2017b). Of the 114,552 km of streams in the NHDPlusV2 dataset, using the current three‐category Chessie BIBI ratings, 71,631 km (62.5%) of these streams can be improved to a higher condition category. Thus, it would take improvement in 7,163 km (4,451 miles) of streams to reach the 10% goal. However, our results suggest that without any restoration activity, a wide spectrum of future conditions is possible, ranging, by 2090, from a watershed‐wide degradation in 16.2% of stream km (A2 CMIP5 p25) to 1.0% of stream km (B2 Lynch2016 scenario). Thus, it may take improvement in 11.0% (7,879 km, 4,896 miles) to 26.2% (18,767 km, 11,661 miles) stream kilometers to assuage effects of possible future land‐use and climate changes and sustain the 10% goal; a range that has dramatically different management implications. For example, at a median cost to restore a stream kilometer in the Chesapeake Bay watershed of $10,500 (Hassett et al., 2005), based on our study, the total cost to reach the 10% goal would range between 82.7 and 197.0 million US dollars. Where to focus restoration efforts also changes among scenarios (Figure 5; Figures S17–S20). Under some scenarios (e.g., B2 CMIP5 p50), a more spatially targeted (central and southern portion) approach may be fruitful, whereas other scenarios (e.g., A2 CMIP5 p50) highlight a watershed‐wide need for intervention to assure more streams are in improved versus degraded conditions.

Globally, our results can be used as an early case study for other regions given the expected relatively early projected temperature changes and population growth in the watershed. As mentioned above, the Chesapeake Bay watershed is in a geographic region expected to experience increased temperatures decades before global averages and that may experience population increases similar to a worst‐case global scenario. Furthermore, the Chesapeake Bay watershed has a restoration goal set by the Chesapeake Bay Program and our results highlight how various possible future scenarios may affect not only attaining such restoration goals but also sustaining them. Other regions will be faced with similar uncertainties in future land‐use and climate scenarios, and therefore should acknowledge these uncertainties when attempting to predict future conditions.

## AUTHOR CONTRIBUTIONS

KOM conceived the study, KOM, KPK, LEH, GJM, and TLS summarized covariates, KOM performed analyses and created figures, CB and ZMS contributed Chessie BIBI data, and all authors contributed substantially to interpreting results and writing. The summarized land‐use and climate projection data that support the findings of this study are openly available in ScienceBase at https://doi.org/10.5066/P9IX5GRJ.

## References

[gcb14961-bib-0001] Allan, J. D. (2004). Landscapes and riverscapes: The influence of land use on stream ecosystems. Annual Review of Ecology, Evolution, and Systematics, 35, 257–284. 10.1146/annurev.ecolsys.35.120202.110122

[gcb14961-bib-0002] Allan, J. D. , Erickson, D. L. , & Fay, J. (1997). The influence of catchment land use on stream integrity across multiple spatial scales. Freshwater Biology, 37, 149–161. 10.1046/j.1365-2427.1997.d01-546.x

[gcb14961-bib-0003] Barbour, M. T. , Gerritsen, J. , Snyder, B. D. , & Stribling, J. B. (1999). Rapid bioassessment protocols for use in streams and wadeable rivers: Periphyton, benthic macroinvertebrates and fish, second edition (EPA 841‐B‐99‐002). Washington, DC. Retrieved from https://gis.lic.wisc.edu/wwwlicgf/glifwc/PolyMet/SDEIS/references/USEPA%202012b.pdf

[gcb14961-bib-0004] Beven, K. J. , & Kirkby, M. J. (1979). A physically based, variable contributing area model of basin hydrology / Un modèle à base physique de zone d'appel variable de l'hydrologie du bassin versant. Hydrological Sciences Bulletin, 24(1), 43–69. 10.1080/02626667909491834

[gcb14961-bib-0005] Bonada, N. , Prat, N. , Resh, V. H. , & Statzner, B. (2006). Developments in aquatic insect biomonitoring: A comparative analysis of recent approaches. Annual Review of Entomology, 51, 495–523. 10.1146/annurev.ento.51.110104.151124 16332221

[gcb14961-bib-0006] Breiman, L. (2001). Random forests. Machine Learning, 45(1), 5–32. 10.1023/A:1010933404324

[gcb14961-bib-0007] Buchanan, C. , Maloney, K. O. , Smith, Z. M. , Nagel, A. , & Young, J. A. (2018). Creating a stream health baseline for the Chesapeake basin from monitoring and model data. Interim report presented to the Stream Health Working Group of the Chesapeake Bay Program. Retrieved from https://www.potomacriver.org/wp-content/uploads/2018/12/Stream-Health-2008-Baseline-Report-12-5-18.pdf

[gcb14961-bib-0008] Carpenter, S. R. , Stanley, E. H. , & Zanden, M. J. V. (2011). State of the world's freshwater ecosystems: Physical, chemical, and biological changes. Annual Review of Environment and Resources, 36(1), 75–99. 10.1146/annurev-environ-021810-094524

[gcb14961-bib-0009] Carter, J. L. , Resh, V. H. , & Hannaford, M. J. (2017). Macroinvertebrates as biotic indicators of environmental quality In Hauer F. R & Lamberti G. A (Eds.), Methods in stream ecology (3rd ed., pp. 293–318). Cambridge, MA: Elsevier 10.1016/b978-0-12-813047-6.00016-4

[gcb14961-bib-0010] Chesapeake Bay Program . (2017a). Facts and figures. Retrieved from https://www.chesapeakebay.net/discover/facts

[gcb14961-bib-0011] Chesapeake Bay Program . (2017b). Stream health management strategy. Retrieved from https://www.chesapeakebay.net/documents/22039/v3_2015-2025_updated_stream_health_management_strategy_public_input_11.8.2019.pdf

[gcb14961-bib-0012] Collen, B. , Whitton, F. , Dyer, E. E. , Baillie, J. E. M. , Cumberlidge, N. , Darwall, W. R. T. , … Böhm, M. (2014). Global patterns of freshwater species diversity, threat and endemism. Global Ecology and Biogeography, 23(1), 40–51. 10.1111/geb.12096 26430385 PMC4579866

[gcb14961-bib-0013] Covich, A. P. , Palmer, M. A. , & Crowl, T. A. (1999). The role of benthic invertebrate species in freshwater ecosystems: Zoobenthic species influence energy flows and nutrient cycling. BioScience, 49(2), 119–127. 10.2307/1313537

[gcb14961-bib-0014] Cutler, D. R. , Edwards, T. C. , Beard, K. H. , Cutler, A. , Hess, K. T. , Gibson, J. , & Lawler, J. J. (2007). Random forests for classification in ecology. Ecology, 88(11), 2783–2792. 10.1890/07-0539.1 18051647

[gcb14961-bib-0015] Davidson, N. C. (2014). How much wetland has the world lost? Long‐term and recent trends in global wetland area. Marine and Freshwater Research, 65(10), 934–941. 10.1071/MF14173

[gcb14961-bib-0016] Dhungel, S. , Tarboton, D. G. , Jin, J. , & Hawkins, C. P. (2016). Potential effects of climate change on ecologically relevant streamflow regimes. River Research and Applications, 32(9), 1827–1840. 10.1002/rra.3029

[gcb14961-bib-0017] Downing, J. A. , Cole, J. J. , Duarte, C. , Middelburg, J. J. , Melack, J. M. , Prairie, Y. T. , … Tranvik, L. J. (2012). Global abundance and size distribution of streams and rivers. Inland Waters, 2, 229–236. 10.5268/IW-2.4.502

[gcb14961-bib-0018] Dupigny‐Giroux, L. A. , Mecray, E. L. , Lemcke‐Stampone, M. D. , Hodgkins, G. A. , Lentz, E. E. , Mills, K. E. , … Caldwell, C. (2018). Northeast In Reidmiller D. R., Avery C. W., Easterling D. R., Kunkel K. E., Lewis K. L. M., Maycock T. K., & Stewart B. C. (Eds.), Impacts, risks, and adaptation in the United States: Fourth national climate assessment (Vol. II, pp. 669–742). Washington, DC: U.S. Global Change Research Program.

[gcb14961-bib-0019] Durance, I. , & Ormerod, S. J. (2007). Climate change effects on upland stream macroinvertebrates over a 25‐year period. Global Change Biology, 13(5), 942–957. 10.1111/j.1365-2486.2007.01340.x

[gcb14961-bib-0020] Fernández‐Delgado, M. , Cernadas, E. , Barro, S. , & Amorim, D. (2014). Do we need hundreds of classifiers to solve real world classification problems. Journal of Machine Learning Research, 15(1), 3133–3181.

[gcb14961-bib-0021] Friedman, J. H. (2001). Greedy function approximation: A gradient boosting machine. The Annals of Statistics, 1189–1232. 10.1214/aos/1013203451

[gcb14961-bib-0022] Guse, B. , Kail, J. , Radinger, J. , Schröder, M. , Kiesel, J. , Hering, D. , … Fohrer, N. (2015). Eco‐hydrologic model cascades: Simulating land use and climate change impacts on hydrology, hydraulics and habitats for fish and macroinvertebrates. Science of the Total Environment, 533, 542–556. 10.1016/j.scitotenv.2015.05.078 26188405

[gcb14961-bib-0023] Hassett, B. , Palmer, M. , Bernhardt, E. , Smith, S. , Carr, J. , & Hart, D. (2005). Restoring watersheds project by project: Trends in Chesapeake Bay tributary restoration. Frontiers in Ecology and the Environment, 3(5), 259–267. 10.1890/1540-9295(2005)003[0259:rwpbpt]2.0.co;2

[gcb14961-bib-0024] Hay, L. E. , & McCabe, G. J. (2019). A summary of CMIP3 and CMIP5 climate change projections for the conterminous U.S. 10.5066/P9V18TM9

[gcb14961-bib-0025] Heino, J. , Virkkala, R. , & Toivonen, H. (2009). Climate change and freshwater biodiversity: Detected patterns, future trends and adaptations in northern regions. Biological Reviews, 84(1), 39–54. 10.1111/j.1469-185x.2008.00060.x 19032595

[gcb14961-bib-0026] Hill, R. A. , Fox, E. W. , Leibowitz, S. G. , Olsen, A. R. , Thornbrugh, D. J. , & Weber, M. H. (2017). Predictive mapping of the biotic condition of conterminous‐USA rivers and streams. Ecological Applications, 27(8), 2397–2415. 10.1002/eap.1617 28871655 PMC5796808

[gcb14961-bib-0027] Hill, R. A. , Weber, M. H. , Leibowitz, S. G. , Olsen, A. R. , & Thornbrugh, D. J. (2016). The Stream‐Catchment (StreamCat) dataset: A database of watershed metrics for the conterminous United States. Journal of the American Water Resources Association, 52(1), 120–128. 10.1111/1752-1688.12372. Retrieved from ftp://newftp.epa.gov/EPADataCommons/ORD/NHDPlusLandscapeAttributes/StreamCat/WelcomePage.html

[gcb14961-bib-0028] Homer, C. , Dewitz, J. , Yang, L. , Jin, S. , Danielson, P. , Xian, G. , … Megown, K. (2015). Completion of the 2011 National Land Cover Database for the conterminous United States–representing a decade of land cover change information. Photogrammetric Engineering & Remote Sensing, 81(5), 345–354.

[gcb14961-bib-0029] Hynes, H. B. N. (1975). Edgardo baldi memorial lecture. The stream and its valley. Verhandlungen der Internationalen Vereinigung für Theoretische und Angewandte Limnologie, 19, 1–15. 10.1080/03680770.1974.11896033

[gcb14961-bib-0030] Kärnä, O.‐M. , Heino, J. , Laamanen, T. , Jyrkänkallio‐Mikkola, J. , Pajunen, V. , Soininen, J. , … Hjort, J. (2019). Does catchment geodiversity foster stream biodiversity? Landscape Ecology, 34, 2469–2485. 10.1007/s10980-019-00901-z

[gcb14961-bib-0031] Knouft, J. H. , & Ficklin, D. L. (2017). The potential impacts of climate change on biodiversity in flowing freshwater systems. Annual Review of Ecology, Evolution, and Systematics, 48, 111–133. 10.1146/annurev-ecolsys-110316-022803

[gcb14961-bib-0032] Krause, K. P. , Chien, H. , Ficklin, D. L. , Hall, D. M. , Schuster, G. A. , Swannack, T. M. , … Knouft, J. H. (2019). Streamflow regimes and geologic conditions are more important than water temperature when projecting future crayfish distribution. Climatic Change, 154, 107–123. 10.1007/s10584-019-02435-4

[gcb14961-bib-0033] Kuemmerlen, M. , Schmalz, B. , Cai, Q. , Haase, P. , Fohrer, N. , & Jähnig, S. C. (2015). An attack on two fronts: Predicting how changes in land use and climate affect the distribution of stream macroinvertebrates. Freshwater Biology, 60(7), 1443–1458. 10.1111/fwb.12580

[gcb14961-bib-0034] Landis, J. R. , & Koch, G. G. (1977). The measurement of observer agreement for categorical data. Biometrics, 33(1), 159–174. 10.2307/2529310 843571

[gcb14961-bib-0035] Li, X. , Zhang, Y. , Guo, F. , Gao, X. , & Wang, Y. (2018). Predicting the effect of land use and climate change on stream macroinvertebrates based on the linkage between structural equation modeling and Bayesian network. Ecological Indicators, 85, 820–831. 10.1016/j.ecolind.2017.11.044

[gcb14961-bib-0036] Liaw, A. , & Wierner, W. (2002). Classification and regression by randomForest. R News, 2(3), 18–22.

[gcb14961-bib-0037] Lynch, C. , Seth, A. , & Thibeault, J. (2016). Recent and projected annual cycles of temperature and precipitation in the northeast United States from CMIP5. Journal of Climate, 29(1), 347–365. 10.1175/jcli-d-14-00781.1

[gcb14961-bib-0038] Maloney, K. O. , Schmid, M. , & Weller, D. E. (2012). Applying additive modelling and gradient boosting to assess the effects of watershed and reach characteristics on riverine assemblages. Methods in Ecology and Evolution, 3(1), 116–128. 10.1111/j.2041-210X.2011.00124.x

[gcb14961-bib-0039] Maloney, K. O. , Smith, Z. M. , Buchanan, C. , Nagel, A. , & Young, J. A. (2018). Predicting biological conditions for small headwater streams in the Chesapeake Bay watershed. Freshwater Science, 37(4), 795–809. 10.1086/700701

[gcb14961-bib-0040] Maloney, K. O. , Weller, D. E. , Russell, M. J. , & Hothorn, T. (2009). Classifying the biological condition of small streams: An example using benthic macroinvertebrates. Journal of the North American Benthological Society, 28(4), 869–884. 10.1899/08-142.1

[gcb14961-bib-0041] Maloney, K. O. , Young, J. A. , Faulkner, S. P. , Hailegiorgis, A. , Slonecker, E. T. , & Milheim, L. E. (2018). A detailed risk assessment of shale gas development on headwater streams in the Pennsylvania portion of the Upper Susquehanna River Basin, USA. Science of the Total Environment, 610, 154–166. 10.1016/j.scitotenv.2017.07.247 28803193

[gcb14961-bib-0042] Mantyka‐Pringle, C. S. , Martin, T. G. , Moffatt, D. B. , Linke, S. , & Rhodes, J. R. (2014). Understanding and predicting the combined effects of climate change and land‐use change on freshwater macroinvertebrates and fish. Journal of Applied Ecology, 51(3), 572–581. 10.1111/1365-2664.12236

[gcb14961-bib-0043] McKay, L. , Bondelid, T. , Dewald, T. , Johnston, J. , Moore, R. , & Rea, A. (2012). NHDPlus version 2: User guide. Retrieved from ftp://ftp.horizonsystems.com/NHDPlus/NHDPlusV21/Documentation/NHDPlus-V2_User_Guide.pdf

[gcb14961-bib-0044] Merriam, E. R. , Petty, J. T. , & Clingerman, J. (2019). Conservation planning at the intersection of landscape and climate change: Brook trout in the Chesapeake Bay watershed. Ecosphere, 10(2), e02585 10.1002/ecs2.2585

[gcb14961-bib-0045] Meyer, J. L. , Sale, M. J. , Mulholland, P. J. , & Poff, N. L. (1999). Impacts of climate change on aquatic ecosystem functioning and health. Journal of the American Water Resources Association, 35(6), 1373–1386. 10.1111/j.1752-1688.1999.tb04222.x

[gcb14961-bib-0046] Meyer, J. L. , Strayer, D. L. , Wallace, J. B. , Eggert, S. L. , Helfman, G. S. , & Leonard, N. E. (2007). The contribution of headwater streams to biodiversity in river networks1. Journal of the American Water Resources Association, 43(1), 86–103. 10.1111/j.1752-1688.2007.00008.x

[gcb14961-bib-0047] Molnar, C. (2019). Interpretable machine learning: A guide for making black box models explainable. 16 March 2019. Christoph Molnar, Leanpub. Retrieved from https://christophm.github.io/interpretable-ml-book/

[gcb14961-bib-0048] Morrill, J. C. , Bales, R. C. , & Conklin, M. H. (2005). Estimating stream temperature from air temperature: Implications for future water quality. Journal of Environmental Engineering, 131(1), 139–146. 10.1061/(asce)0733-9372(2005)131:1(139)

[gcb14961-bib-0049] Moss, R. H. , Edmonds, J. A. , Hibbard, K. A. , Manning, M. R. , Rose, S. K. , van Vuuren, D. P. , … Wilbanks, T. J. (2010). The next generation of scenarios for climate change research and assessment. Nature, 463(7282), 747–756. 10.1038/nature08823 20148028

[gcb14961-bib-0050] Mustonen, K.‐R. , Mykrä, H. , Marttila, H. , Sarremejane, R. , Veijalainen, N. , Sippel, K. , … Hawkins, C. P. (2018). Thermal and hydrologic responses to climate change predict marked alterations in boreal stream invertebrate assemblages. Global Change Biology, 24(6), 2434–2446. 10.1111/gcb.14053 29341358

[gcb14961-bib-0051] Nakicenovic, N. , Alcamo, J. , Grubler, A. , Riahi, K. , Roehrl, R. , Rogner, H.‐H. , & Victor, N. (2000). Special report on emissions scenarios (SRES), a special report of Working Group III of the intergovernmental panel on climate change. New York, NY: Cambridge University Press.

[gcb14961-bib-0052] Nelson, K. C. , Palmer, M. A. , Pizzuto, J. E. , Moglen, G. E. , Angermeier, P. L. , Hilderbrand, R. H. , … Hayhoe, K. (2009). Forecasting the combined effects of urbanization and climate change on stream ecosystems: From impacts to management options. Journal of Applied Ecology, 46(1), 154–163. 10.1111/j.1365-2664.2008.01599.x 19536343 PMC2695864

[gcb14961-bib-0053] Northrup, J. M. , Rivers, J. W. , Yang, Z. , & Betts, M. G. (2019). Synergistic effects of climate and land‐use change influence broad‐scale avian population declines. Global Change Biology, 25(5), 1561–1575. 10.1111/gcb.14571 30810257

[gcb14961-bib-0054] Oliver, T. H. , & Morecroft, M. D. (2014). Interactions between climate change and land use change on biodiversity: Attribution problems, risks, and opportunities. Wiley Interdisciplinary Reviews: Climate Change, 5(3), 317–335. 10.1002/wcc.271

[gcb14961-bib-0055] Olson, J. R. , & Hawkins, C. P. (2012). Predicting natural base‐flow stream water chemistry in the western United States. Water Resources Research, 48(2), 10.1029/2011wr011088

[gcb14961-bib-0056] Poff, N. L. R. , Allan, J. D. , Bain, M. B. , Karr, J. R. , Prestegaard, K. L. , Richter, B. D. , … Stromberg, J. C. (1997). The natural flow regime: A paradigm for river conservation and restoration. BioScience, 47(11), 769–784. 10.2307/1313099

[gcb14961-bib-0057] Pyne, M. I. , & Poff, N. L. (2017). Vulnerability of stream community composition and function to projected thermal warming and hydrologic change across ecoregions in the western United States. Global Change Biology, 23(1), 77–93. 10.1111/gcb.13437 27429092

[gcb14961-bib-0058] R Development Core Team . (2019). R: A language and environment for statistical computing. Version 3.6.0. Vienna, Austria: R Foundation for Statistical Computing Retrieved from https://www.r-project.org

[gcb14961-bib-0059] Radinger, J. , Hölker, F. , Horký, P. , Slavík, O. , Dendoncker, N. , & Wolter, C. (2016). Synergistic and antagonistic interactions of future land use and climate change on river fish assemblages. Global Change Biology, 22(4), 1505–1522. 10.1111/gcb.13183 26649996

[gcb14961-bib-0060] Roy, A. H. , Rosemond, A. D. , Leigh, D. S. , Paul, M. J. , & Wallace, J. B. (2003). Habitat‐specific responses of stream insects to land cover disturbance: Biological consequences and monitoring implications. Journal of the North American Benthological Society, 22(2), 292–307. 10.2307/1467999

[gcb14961-bib-0061] Scott, M. C. , Helfman, G. S. , McTammany, M. E. , Benfield, E. F. , & Bolstad, P. V. (2002). Multiscale influences on physical and chemical stream conditions across Blue Ridge landscapes. Journal of the American Water Resources Association, 38(5), 1379–1392. 10.1111/j.1752-1688.2002.tb04353.x

[gcb14961-bib-0062] Sleeter, B. M. , Sohl, T. L. , Bouchard, M. A. , Reker, R. R. , Soulard, C. E. , Acevedo, W. , … Zhu, Z. (2012). Scenarios of land use and land cover change in the conterminous United States: Utilizing the special report on emission scenarios at ecoregional scales. Global Environmental Change, 22(4), 896–914. 10.1016/j.gloenvcha.2012.03.008

[gcb14961-bib-0063] Smith, Z. M. , Buchanan, C. , & Nagel, A. (2017). Refinement of the basin‐wide Index of Biotic Integrity for non‐tidal streams and wadeable rivers in the Chesapeake Bay watershed. ICPRB Report 17‐2. Retrieved from https://www.potomacriver.org/wp-ontent/uploads/2017/05/ChessieBIBI_Report_Final_5-25-2017.pdf

[gcb14961-bib-0064] Sohl, T. L. , Sayler, K. L. , Bouchard, M. A. , Reker, R. R. , Friesz, A. M. , Bennett, S. L. , … Van Hofwegen, T. (2014). Spatially explicit modeling of 1992–2100 land cover and forest stand age for the conterminous United States. Ecological Applications, 24(5), 1015–1036. 10.1890/13-1245.1 25154094

[gcb14961-bib-0065] Sörensen, R. , Zinko, U. , & Seibert, J. (2006). On the calculation of the topographic wetness index: Evaluation of different methods based on field observations. Hydrology and Earth System Sciences Discussions, 10(1), 101–112. 10.5194/hessd-2-1807-2005

[gcb14961-bib-0066] Strayer, D. L. , & Dudgeon, D. (2010). Freshwater biodiversity conservation: Recent progress and future challenges. Journal of the North American Benthological Society, 29(1), 344–358. 10.1899/08-171.1

[gcb14961-bib-0067] Van Vuuren, D. P. , & Carter, T. R. (2014). Climate and socio‐economic scenarios for climate change research and assessment: Reconciling the new with the old. Climatic Change, 122(3), 415–429. 10.1007/s10584-013-0974-2

[gcb14961-bib-0068] Vannote, R. L. , Minshall, G. W. , Cummins, K. W. , Sedell, J. R. , & Cushing, C. E. (1980). The river continuum concept. Canadian Journal of Fisheries and Aquatic Sciences, 37, 130–137. 10.1139/f80-017

[gcb14961-bib-0069] Viger, R. J. , & Bock, A. (2014). GIS features of the geospatial fabric for national hydrologic modeling. 10.5066/F7542KMD

[gcb14961-bib-0070] Vörösmarty, C. J. , McIntyre, P. B. , Gessner, M. O. , Dudgeon, D. , Prusevich, A. , Green, P. , … Davies, P. M. (2010). Global threats to human water security and river biodiversity. Nature, 467(7315), 555–561. 10.1038/nature09440 20882010

[gcb14961-bib-0071] Walsh, C. J. , Roy, A. H. , Feminella, J. W. , Cottingham, P. D. , Groffman, P. M. , & Morgan, R. P. (2005). The urban stream syndrome: Current knowledge and the search for a cure. Journal of the North American Benthological Society, 24(3), 706–723. 10.1899/0887-3593(2005)0240706:tussck2.0.co;2

[gcb14961-bib-0072] Waters, T. F. (1995). Sediment in streams: Sources, biological effects, and control. Bethesda, MD: American Fisheries Society.

[gcb14961-bib-0073] Woodward, G. , Perkins, D. M. , & Brown, L. E. (2010). Climate change and freshwater ecosystems: Impacts across multiple levels of organization. Philosophical Transactions of the Royal Society B: Biological Sciences, 365(1549), 2093–2106. 10.1098/rstb.2010.0055 PMC2880135 20513717

[gcb14961-bib-0074] Young, H. S. , McCauley, D. J. , Galetti, M. , & Dirzo, R. (2016). Patterns, causes, and consequences of anthropocene defaunation. Annual Review of Ecology, Evolution, and Systematics, 47, 333–358. 10.1146/annurev-ecolsys-112414-054142

