# Supplementary material for: Disentangling the potential effects of land‐use and climate change on stream conditions
Source: Glob Chang Biol. 2020 Jan 19;26(4):2251–69. doi: 10.1111/gcb.14961 (PMC7155133; doi:10.1111/gcb.14961)
Supplement: Supplementary file 1 [file GCB-26-2251-s001.docx]

Appendix 1. Detailed description of the processing behind the model projected changes in seasonal temperature and precipitation in Hay & McCabe (2019)

Projections of future temperature (T) and precipitation (P) were obtained from 122 general circulation models (GCMs) that include a range of greenhouse gas emission scenarios. The outputs from the GCMs were statistically downscaled. Statistical downscaling is a technique for deriving finer-scale interpolations from coarse-scale GCMs by deriving statistical relations between observed local-scale climate data, such as meteorological observations or gridded station data derived from historical climate observations, and the coarser-scale GCM variables (Wood and others, 2004). There are a number of different downscaling procedures and datasets available. The statistically downscaled GCM simulations used here are from the bias-corrected spatially disaggregated (BCSD) Coupled Model Intercomparison Project (CMIP) 5 projections (Bureau of Reclamation 2011, 2013). These data are available on a monthly time-step with a spatial resolution of approximately 1/8 degree.

CMIP 5 model projections are composed of a number of different scenarios, the primary ones being the Representative Concentration Pathways (RCP) 2.6, 4.5, 6, and 8.5(Bureau of Reclamation, 2011, 2013). RCP2.6 from CMIP5 was excluded in Hay & MacCabe (2019) to balance the number of emission scenarios with other analyses. These scenarios represent assumptions about future greenhouse gas emissions by taking into account short- and long-term climate cycles, and anthropogenic drivers such as changes in demographics, and economic and technological development (IPCC, 2000; Taylor et al., 2012). Climate conditions represented by these scenarios range from stabilized populations after 2050, coupled with rapid development of more efficient technological systems across the globe (RCP4.5), to globally increasing populations and regionally-orientated economic development (RCP8.5). For a full description of the differences between the RCPs from CMIP5, consult Taylor et al. (2012).

For each downscaled GCM, the historical conditions (or current climatic conditions) are represented by climate model output for 1980 through 1999, whereas future climatic conditions are represented by model simulations for 2005 through 2099. The data for the GCM simulations used in this study were obtained from the U.S. Geological Survey Geo Data Portal (GDP) (Blodgett et al., 2013).

The 122 selected climate model projections were summarized using the GDP for hydrologic response units (HRUs) from the Geospatial Fabric for National Hydrologic Modeling (Viger and Bock, 2014). The Geospatial Fabric is a set of hydrographic features aggregated from the National Hydrography Dataset Plus (US Geological Survey and Environmental Protection Agency, 2010) at a scale appropriate for regional and national hydrologic modeling and analysis. An HRU is a land surface unit that contributes runoff (R) to the stream network, and is a derivative of the traditional contributing area, or watershed, and represents an area of homogenous conditions (such as areas of similar features, such as slope, soils type, or topography) where a given amount of P is expected to yield a similar hydrologic response. For the analyses in this study the climate model projections were summarized for 109,951 HRUs across the conterminous United States (CONUS).

For each climate model, projections of future T and P were summarized as changes (i.e. departures) from a 20-year historical baseline (i.e. 1980 through 1999). Thus, all projected values of T and P were expressed as a departure from mean T and P for 1980 through 1999 simulated by each respective model for each of the 109,951 HRUs. Additionally, projections of monthly T and P were aggregated to values of seasonal mean T and seasonal total P. The seasons were defined as: winter (January through March), spring (April through June), summer (July through September), and Fall (October through December). Departures of T and P were expressed as mean seasonal departures for three 19-year future periods centered on 2030 (2021 through 2039), 2060 (2051 through 2069), and 2090 (2081 through 2099). The historical baseline period (1980 through 1999) is referred to as the baseline, while the three future periods are referred to as 2030, 2060, and 2090 periods.

For each variable (i.e. T and P), each season, and each period (i.e. 2030, 2060, and 2090) the 5th, 25th, 50th, 75th, and 95th percentiles of T and P projections from the 122 climates models were computed for each of the 109,951 HRUs. The percentiles were subsequently mapped to illustrate the distributions of changes in T and P for each season and each future period.

References

Blodgett, D. L., 2013, The U.S. Geological Survey Climate Geo Data Portal: An integrated broker for climate and geospatial data. U.S. Geological Survey Fact Sheet 2013-3019, 2 pp.

Bureau of Reclamation, 2011. West-Wide Climate Risk Assessments: Bias-corrected and spatially downscaled surface water projections, Technical Memorandum No. 86-68210-2011-01, prepared by the U.S. Department of the Interior, Bureau of Reclamation, Technical Services Center, Denver, Colorado. 138pp.

Bureau of Reclamation, 2013. Downscaled CMIP3 and CMIP5 climate and hydrology projections: release of hydrology projections, comparison with preceding Information, and summary of user needs, prepared by the U.S. Department of the Interior, Bureau of Reclamation, Technical Services Center, Denver, Colorado. 110 pp.

Taylor, K.E., R.J. Stouffer, and G.A. Meehl. 2012. An overview of CMIP5 and the experimental design. Bulletin of the American Meteorological Society, 93: 485-498, doi:10.1175/BAMS-D-11-00094.1.

Wood, A.W., Leung, L.R., Sridhar, V. et al. Climatic Change (2004) 62: 189. https://doi.org/10.1023/B:CLIM.0000013685.99609.9e

Viger, Roland J. and Bock, Andrew, 2014, GIS Features of the Geospatial Fabric for National Hydrologic Modeling, US Geological Survey. http://dx.doi.org/doi:10.5066/F7542KMD

Table S1. Summary statistics for 35 covariates for the Chessie BIBI training and NHDplusV2 data sets. Bioregion was not summarized because it was a categorical covariate.

|  |  |  | Training Data set (n = 2,775) | | | |  | NHDplusV2 Data set (n = 70,772) | | | |
| --- | --- | --- | --- | --- | --- | --- | --- | --- | --- | --- | --- |
| Covariate name | Covariate description | Data source | Mean | Median | Min | Max |  | Mean | Median | Min | Max |
| WsAreaSqKm | Upstream watershed area (km^2^) at NHDplus stream segment outlet | USEPA - StreamCat | 34.1 | 16.1 | 0.3 | 199.7 |  | 17.7 | 4.4 | <0.1 | 200.0 |
| ElevWs | Upstream mean elevation (m) | USEPA - StreamCat | 327.5 | 324.8 | 1.3 | 1224.8 |  | 272.3 | 215.1 | -0.1 | 1336.0 |
| ClayWs | Mean % clay content of soils (STATSGO) within upstream watershed | USEPA - StreamCat | 21.7 | 19.5 | 8.1 | 57.0 |  | 22.2 | 19.5 | 3.0 | 59.2 |
| SandWs | Mean % sand content of soils (STATSGO) within upstream watershed | USEPA - StreamCat | 32.0 | 29.4 | 9.5 | 77.0 |  | 33.5 | 31.2 | 9.5 | 94.9 |
| OmWs | Mean organic matter content (% by weight) of soils (STATSGO) within upstream watershed | USEPA - StreamCat | 0.8 | 0.6 | 0.1 | 22.7 |  | 0.8 | 0.6 | 0.1 | 31.7 |
| PermWs | Mean permeability (cm/hour) of soils (STATSGO) within upstream watershed | USEPA - StreamCat | 8.7 | 7.6 | 2.0 | 27.2 |  | 8.9 | 7.7 | 1.3 | 41.9 |
| RckDepWs | Mean depth (cm) to bedrock of soils (STATSGO) within upstream watershed | USEPA - StreamCat | 125.8 | 130.2 | 73.2 | 183.2 |  | 129.2 | 133.2 | 57.3 | 191.5 |
| WtDepWs | Mean seasonal water table depth (cm) of soils (STATSGO) within upstream watershed | USEPA - StreamCat | 139.3 | 151.2 | 15.5 | 182.9 |  | 134.1 | 148.9 | 1.4 | 182.9 |
| KffactWs | Mean soil erodibility (Kf) factor (unitless) of soils within upstream watershed. | USEPA - StreamCat | 0.3 | 0.3 | <0.1 | 0.4 |  | 0.3 | 0.3 | <0.1 | 0.4 |
| RunoffWs | Mean runoff (mm) within upstream watershed | USEPA - StreamCat | 467.1 | 438.7 | 316.0 | 680.0 |  | 461.2 | 438.0 | 316.0 | 731.0 |
| CaOWs | Mean % of lithological calcium oxide (CaO) content in surface or near surface geology within upstream watershed | USEPA - StreamCat | 5.9 | 2.5 | 0.4 | 39.8 |  | 5.3 | 2.2 | 0.3 | 45.6 |
| HydrlCondWs | Mean lithological hydraulic conductivity (micrometers per second) content in surface or near surface geology within upstream watershed | USEPA - StreamCat | 5.8 | 0.2 | <0.1 | 215.1 |  | 6.6 | 0.2 | <0.1 | 215.1 |
| CompStrgthWs | Mean lithological uniaxial compressive strength (megaPascals) content in surface or near surface geology within upstream watershed | USEPA - StreamCat | 80.2 | 78.1 | 0.5 | 223.8 |  | 69.7 | 73.8 | 0.4 | 242.4 |
| WetIndexWs | Mean Composite Topographic Index (CTI)[Wetness Index] within upstream watershed | USEPA - StreamCat | 756.3 | 743.8 | 560.4 | 1423.7 |  | 774.8 | 750.5 | 501.8 | 2050.5 |
| BFIWs | Ratio of base flow to total flow, expressed as a percentage, within upstream watershed | USEPA - StreamCat | 45.3 | 44.5 | 24.9 | 66.0 |  | 46.1 | 46.0 | 23.7 | 66.8 |
| PrecipWs_amj | Mean upstream watershed total precipitation for spring (April, May, June) over 1980-1999 (mm) | PRISM | 315.8 | 314.8 | 256.2 | 446.0 |  | 314.4 | 313.7 | 244.0 | 446.8 |
| PrecipWs_jas | Mean upstream watershed total precipitation for summer (July, August, September) over 1980-1999 (mm) | PRISM | 331.0 | 328.4 | 249.2 | 448.6 |  | 338.6 | 334.2 | 240.6 | 471.9 |
| PrecipWs_jfm | Mean upstream watershed total precipitation for winter (January, February, March) over 1980-1999 (mm) | PRISM | 221.9 | 223.1 | 155.6 | 345.5 |  | 222.7 | 223.1 | 149.2 | 355.0 |
| PrecipWs_ond | Mean upstream watershed total precipitation for fall (October, November, December) over 1980-1999 (mm) | PRISM | 274.5 | 278.7 | 182.0 | 390.7 |  | 277.6 | 280.4 | 179.4 | 402.9 |
| TempWs_amj | Mean upstream watershed average temperature for spring (April, May, June) over 1980-1999 (°C) | PRISM | 15.7 | 16.0 | 11.4 | 20.4 |  | 16.3 | 16.8 | 11.1 | 20.5 |
| TempWs_jas | Mean upstream watershed average temperature for summer (July, August, September) over 1980-1999 (°C) | PRISM | 21.0 | 21.1 | 16.3 | 25.4 |  | 21.6 | 22.0 | 16.1 | 25.6 |
| TempWs_jfm | Mean upstream watershed average temperature for winter (January, February, March) over 1980-1999 (°C) | PRISM | 0.9 | 1.3 | -5.0 | 7.2 |  | 1.8 | 2.4 | -5.2 | 7.3 |
| TempWs_ond | Mean upstream watershed average temperature for fall (October, November, December) over 1980-1999 (°C) | PRISM | 6.4 | 6.5 | 2.3 | 12.0 |  | 7.1 | 7.3 | 2.1 | 12.4 |
| Water | % of upstream watershed classified as water land cover | USGS - EROS | 0.4 | 0.0 | 0.0 | 13.4 |  | 1.7 | 0.0 | 0.0 | 100.0 |
| Developed | % of upstream watershed classified as developed land cover | USGS - EROS | 5.5 | 0.3 | 0.0 | 100.0 |  | 6.1 | 0.0 | 0.0 | 100.0 |
| MechDist | % of upstream watershed classified as mechanically disturbed land cover | USGS - EROS | 0.9 | 0.0 | 0.0 | 23.1 |  | 1.2 | 0.0 | 0.0 | 81.4 |
| Mining | % of upstream watershed classified as mining land cover | USGS - EROS | 0.4 | 0.0 | 0.0 | 35.8 |  | 0.4 | 0.0 | 0.0 | 100.0 |
| Barren | % of upstream watershed classified as barren land cover | USGS - EROS | <0.1 | 0.0 | 0.0 | 0.5 |  | <0.1 | 0.0 | 0.0 | 33.0 |
| Forest_Deciduous | % of upstream watershed classified as deciduous forest land cover | USGS - EROS | 46.7 | 46.3 | 0.0 | 100.0 |  | 42.7 | 40.9 | 0.0 | 100.0 |
| Forest_Evergreen | % of upstream watershed classified as evergreen forest land cover | USGS - EROS | 5.4 | 3.3 | 0.0 | 52.2 |  | 6.2 | 3.6 | 0.0 | 100.0 |
| Forest_Mixed | % of upstream watershed classified as mixed forest land cover | USGS - EROS | 12.0 | 9.7 | 0.0 | 64.4 |  | 12.5 | 9.4 | 0.0 | 100.0 |
| Cropland | % of upstream watershed classified as cropland land cover | USGS - EROS | 6.3 | 2.2 | 0.0 | 69.6 |  | 7.1 | 2.3 | 0.0 | 100.0 |
| Hay_Pasture | % of upstream watershed classified as hay/pasture land cover | USGS - EROS | 20.8 | 15.0 | 0.0 | 92.5 |  | 19.1 | 12.8 | 0.0 | 100.0 |
| Wetland_Herbaceous | % of upstream watershed classified as herbaceous wetland land cover | USGS - EROS | 0.2 | 0.0 | 0.0 | 11.6 |  | 1.2 | 0.0 | 0.0 | 100.0 |
| Wetland_Woody | % of upstream watershed classified as woody wetland land cover | USGS - EROS | 1.3 | 0.0 | 0.0 | 75.0 |  | 1.8 | 0.0 | 0.0 | 100.0 |

Table S2. Values added to the PRISM baseline data for the Lynch2016 model scenario (Lynch et al. 2016). For 2030, values were estimated as half the reported differences for 2060.

|  | Temperature (°C) | | |  | Precipitation (mm) | | |
| --- | --- | --- | --- | --- | --- | --- | --- |
|  | 2030 | 2060 | 2090 |  | 2030 | 2060 | 2090 |
| Winter | 1.9 | 3.9 | 6.3 |  | 21 | 42 | 63 |
| Spring | 1.6 | 3.2 | 5.2 |  | 21 | 39 | 63 |
| Summer | 1.7 | 3.4 | 5.6 |  | 6 | 12 | 12 |
| Fall | 1.7 | 3.3 | 5.4 |  | 6 | 12 | 18 |

Table S3. Correlation matrix for covariates used in the training data set. Cells highlighted in gray indicate the absolute value of the Pearson correlation coefficients ≥ 0.70.

| Variable | WsAreaSqKm | ElevWs | ClayWs | SandWs | OmWs | PermWs | RckDepWs | WtDepWs | KffactWs | RunoffWs | CaOWs | HydrlCondWs | CompStrgthWs | WetIndexWs | BFIWs | PrecipWs_amj | PrecipWs_jas | PrecipWs_jfm |
| --- | --- | --- | --- | --- | --- | --- | --- | --- | --- | --- | --- | --- | --- | --- | --- | --- | --- | --- |
| WsAreaSqKm | 1.00 | 0.17 | -0.03 | -0.15 | 0.11 | -0.08 | -0.05 | -0.07 | 0.06 | 0.11 | 0.09 | -0.10 | 0.07 | -0.06 | -0.03 | 0.03 | 0.00 | 0.03 |
| ElevWs | 0.17 | 1.00 | -0.18 | -0.41 | 0.16 | 0.06 | -0.55 | 0.02 | 0.00 | 0.24 | 0.19 | -0.29 | 0.24 | -0.60 | -0.40 | 0.16 | -0.22 | 0.00 |
| ClayWs | -0.03 | -0.18 | 1.00 | -0.43 | -0.22 | -0.40 | 0.21 | 0.33 | 0.21 | -0.19 | 0.38 | -0.02 | 0.43 | -0.05 | 0.00 | -0.15 | -0.17 | -0.28 |
| SandWs | -0.15 | -0.41 | -0.43 | 1.00 | -0.15 | 0.63 | 0.26 | -0.08 | -0.64 | -0.12 | -0.38 | 0.19 | -0.52 | 0.50 | 0.31 | -0.04 | 0.32 | 0.18 |
| OmWs | 0.11 | 0.16 | -0.22 | -0.15 | 1.00 | -0.16 | -0.06 | -0.44 | 0.04 | 0.02 | 0.00 | -0.05 | -0.13 | 0.07 | -0.18 | -0.07 | -0.02 | -0.05 |
| PermWs | -0.08 | 0.06 | -0.40 | 0.63 | -0.16 | 1.00 | -0.24 | 0.21 | -0.69 | -0.03 | -0.02 | 0.05 | -0.27 | 0.08 | 0.13 | 0.01 | -0.03 | -0.03 |
| RckDepWs | -0.05 | -0.55 | 0.21 | 0.26 | -0.06 | -0.24 | 1.00 | -0.32 | -0.05 | -0.02 | -0.08 | 0.22 | -0.19 | 0.56 | 0.35 | -0.15 | 0.34 | 0.24 |
| WtDepWs | -0.07 | 0.02 | 0.33 | -0.08 | -0.44 | 0.21 | -0.32 | 1.00 | -0.11 | -0.28 | 0.26 | -0.10 | 0.48 | -0.48 | 0.08 | 0.13 | -0.29 | -0.29 |
| KffactWs | 0.06 | 0.00 | 0.21 | -0.64 | 0.04 | -0.69 | -0.05 | -0.11 | 1.00 | -0.04 | 0.06 | 0.15 | 0.11 | -0.15 | -0.14 | 0.00 | -0.14 | -0.05 |
| RunoffWs | 0.11 | 0.24 | -0.19 | -0.12 | 0.02 | -0.03 | -0.02 | -0.28 | -0.04 | 1.00 | -0.07 | -0.10 | -0.08 | -0.06 | -0.03 | 0.10 | 0.34 | 0.47 |
| CaOWs | 0.09 | 0.19 | 0.38 | -0.38 | 0.00 | -0.02 | -0.08 | 0.26 | 0.06 | -0.07 | 1.00 | -0.15 | 0.24 | -0.13 | 0.08 | -0.03 | -0.30 | -0.24 |
| HydrlCondWs | -0.10 | -0.29 | -0.02 | 0.19 | -0.05 | 0.05 | 0.22 | -0.10 | 0.15 | -0.10 | -0.15 | 1.00 | -0.34 | 0.16 | 0.00 | -0.05 | 0.15 | 0.04 |
| CompStrgthWs | 0.07 | 0.24 | 0.43 | -0.52 | -0.13 | -0.27 | -0.19 | 0.48 | 0.11 | -0.08 | 0.24 | -0.34 | 1.00 | -0.47 | -0.03 | 0.20 | -0.30 | -0.12 |
| WetIndexWs | -0.06 | -0.60 | -0.05 | 0.50 | 0.07 | 0.08 | 0.56 | -0.48 | -0.15 | -0.06 | -0.13 | 0.16 | -0.47 | 1.00 | 0.38 | -0.11 | 0.30 | 0.23 |
| BFIWs | -0.03 | -0.40 | 0.00 | 0.31 | -0.18 | 0.13 | 0.35 | 0.08 | -0.14 | -0.03 | 0.08 | 0.00 | -0.03 | 0.38 | 1.00 | 0.02 | 0.27 | 0.29 |
| PrecipWs_amj | 0.03 | 0.16 | -0.15 | -0.04 | -0.07 | 0.01 | -0.15 | 0.13 | 0.00 | 0.10 | -0.03 | -0.05 | 0.20 | -0.11 | 0.02 | 1.00 | 0.28 | 0.51 |
| PrecipWs_jas | 0.00 | -0.22 | -0.17 | 0.32 | -0.02 | -0.03 | 0.34 | -0.29 | -0.14 | 0.34 | -0.30 | 0.15 | -0.30 | 0.30 | 0.27 | 0.28 | 1.00 | 0.61 |
| PrecipWs_jfm | 0.03 | 0.00 | -0.28 | 0.18 | -0.05 | -0.03 | 0.24 | -0.29 | -0.05 | 0.47 | -0.24 | 0.04 | -0.12 | 0.23 | 0.29 | 0.51 | 0.61 | 1.00 |
| PrecipWs_ond | 0.03 | -0.25 | -0.14 | 0.19 | -0.08 | -0.13 | 0.39 | -0.27 | 0.02 | 0.40 | -0.28 | 0.09 | -0.06 | 0.32 | 0.39 | 0.45 | 0.72 | 0.84 |
| TempWs_amj | -0.24 | -0.82 | 0.34 | 0.46 | -0.33 | 0.10 | 0.39 | 0.31 | -0.14 | -0.45 | -0.09 | 0.29 | -0.08 | 0.42 | 0.39 | -0.08 | 0.13 | -0.11 |
| TempWs_jas | -0.24 | -0.88 | 0.29 | 0.47 | -0.30 | 0.08 | 0.44 | 0.24 | -0.12 | -0.42 | -0.11 | 0.29 | -0.12 | 0.49 | 0.42 | -0.07 | 0.17 | -0.07 |
| TempWs_jfm | -0.24 | -0.74 | 0.36 | 0.46 | -0.34 | 0.14 | 0.33 | 0.35 | -0.17 | -0.50 | -0.07 | 0.28 | -0.07 | 0.37 | 0.36 | -0.08 | 0.09 | -0.17 |
| TempWs_ond | -0.24 | -0.81 | 0.29 | 0.51 | -0.30 | 0.13 | 0.41 | 0.25 | -0.16 | -0.47 | -0.11 | 0.30 | -0.14 | 0.47 | 0.41 | -0.08 | 0.16 | -0.10 |
| Water | 0.05 | -0.09 | -0.05 | 0.06 | 0.02 | -0.08 | 0.12 | -0.14 | 0.05 | 0.03 | -0.11 | 0.06 | -0.11 | 0.12 | 0.02 | 0.06 | 0.17 | 0.14 |
| Developed | -0.07 | -0.29 | 0.09 | 0.12 | -0.06 | -0.04 | 0.21 | 0.01 | -0.01 | -0.02 | -0.06 | 0.20 | -0.10 | 0.19 | 0.00 | -0.01 | 0.13 | 0.03 |
| MechDist | -0.10 | -0.25 | 0.03 | 0.29 | -0.06 | 0.10 | 0.26 | -0.15 | -0.19 | 0.03 | -0.18 | 0.17 | -0.30 | 0.24 | 0.02 | -0.14 | 0.29 | 0.05 |
| Mining | -0.01 | 0.11 | -0.02 | -0.02 | -0.04 | 0.13 | -0.06 | -0.02 | -0.05 | 0.14 | 0.01 | -0.03 | -0.02 | 0.00 | -0.03 | 0.02 | -0.01 | 0.08 |
| Barren | 0.02 | -0.05 | -0.04 | 0.11 | 0.00 | 0.06 | 0.05 | -0.01 | -0.09 | -0.04 | -0.03 | 0.00 | -0.08 | 0.06 | 0.03 | -0.01 | 0.01 | 0.01 |
| Forest_Deciduous | 0.07 | 0.64 | -0.19 | -0.20 | -0.03 | 0.20 | -0.50 | 0.11 | -0.05 | 0.19 | -0.04 | -0.14 | 0.20 | -0.59 | -0.36 | 0.12 | -0.13 | 0.00 |
| Forest_Evergreen | -0.05 | -0.09 | -0.01 | 0.21 | -0.04 | 0.09 | 0.10 | -0.17 | -0.08 | 0.01 | -0.19 | 0.13 | -0.22 | 0.17 | 0.02 | -0.07 | 0.23 | 0.10 |
| Forest_Mixed | -0.03 | 0.13 | 0.00 | 0.00 | 0.18 | 0.00 | 0.00 | -0.09 | -0.15 | 0.01 | -0.09 | 0.04 | -0.05 | -0.13 | -0.16 | -0.05 | 0.10 | -0.07 |
| Cropland | -0.05 | -0.47 | -0.17 | 0.46 | -0.03 | 0.13 | 0.40 | -0.25 | -0.06 | -0.06 | -0.04 | 0.07 | -0.43 | 0.65 | 0.48 | -0.10 | 0.20 | 0.19 |
| Hay_Pasture | 0.05 | -0.26 | 0.28 | -0.28 | -0.01 | -0.37 | 0.12 | 0.17 | 0.26 | -0.20 | 0.28 | -0.14 | 0.27 | 0.09 | 0.21 | -0.03 | -0.25 | -0.17 |
| Wetland_Herbaceous | -0.05 | -0.21 | -0.02 | 0.20 | 0.04 | 0.05 | 0.18 | -0.11 | -0.06 | -0.07 | -0.06 | 0.05 | -0.17 | 0.26 | 0.11 | -0.02 | 0.13 | 0.10 |
| Wetland_Woody | -0.06 | -0.34 | -0.13 | 0.48 | 0.26 | 0.18 | 0.29 | -0.30 | -0.20 | -0.09 | -0.15 | 0.13 | -0.40 | 0.59 | 0.27 | -0.01 | 0.18 | 0.16 |

Table S3. Continued

| Variable | PrecipWs_ond | TempWs_amj | TempWs_jas | TempWs_jfm | TempWs_ond | Water | Developed | MechDist | Mining | Barren | Forest_Deciduous | Forest_Evergreen | Forest_Mixed | Cropland | Hay_Pasture | Wetland_Herbaceous | Wetland_Woody |
| --- | --- | --- | --- | --- | --- | --- | --- | --- | --- | --- | --- | --- | --- | --- | --- | --- | --- |
| WsAreaSqKm | 0.03 | -0.24 | -0.24 | -0.24 | -0.24 | 0.05 | -0.07 | -0.10 | -0.01 | 0.02 | 0.07 | -0.05 | -0.03 | -0.05 | 0.05 | -0.05 | -0.06 |
| ElevWs | -0.25 | -0.82 | -0.88 | -0.74 | -0.81 | -0.09 | -0.29 | -0.25 | 0.11 | -0.05 | 0.64 | -0.09 | 0.13 | -0.47 | -0.26 | -0.21 | -0.34 |
| ClayWs | -0.14 | 0.34 | 0.29 | 0.36 | 0.29 | -0.05 | 0.09 | 0.03 | -0.02 | -0.04 | -0.19 | -0.01 | 0.00 | -0.17 | 0.28 | -0.02 | -0.13 |
| SandWs | 0.19 | 0.46 | 0.47 | 0.46 | 0.51 | 0.06 | 0.12 | 0.29 | -0.02 | 0.11 | -0.20 | 0.21 | 0.00 | 0.46 | -0.28 | 0.20 | 0.48 |
| OmWs | -0.08 | -0.33 | -0.30 | -0.34 | -0.30 | 0.02 | -0.06 | -0.06 | -0.04 | 0.00 | -0.03 | -0.04 | 0.18 | -0.03 | -0.01 | 0.04 | 0.26 |
| PermWs | -0.13 | 0.10 | 0.08 | 0.14 | 0.13 | -0.08 | -0.04 | 0.10 | 0.13 | 0.06 | 0.20 | 0.09 | 0.00 | 0.13 | -0.37 | 0.05 | 0.18 |
| RckDepWs | 0.39 | 0.39 | 0.44 | 0.33 | 0.41 | 0.12 | 0.21 | 0.26 | -0.06 | 0.05 | -0.50 | 0.10 | 0.00 | 0.40 | 0.12 | 0.18 | 0.29 |
| WtDepWs | -0.27 | 0.31 | 0.24 | 0.35 | 0.25 | -0.14 | 0.01 | -0.15 | -0.02 | -0.01 | 0.11 | -0.17 | -0.09 | -0.25 | 0.17 | -0.11 | -0.30 |
| KffactWs | 0.02 | -0.14 | -0.12 | -0.17 | -0.16 | 0.05 | -0.01 | -0.19 | -0.05 | -0.09 | -0.05 | -0.08 | -0.15 | -0.06 | 0.26 | -0.06 | -0.20 |
| RunoffWs | 0.40 | -0.45 | -0.42 | -0.50 | -0.47 | 0.03 | -0.02 | 0.03 | 0.14 | -0.04 | 0.19 | 0.01 | 0.01 | -0.06 | -0.20 | -0.07 | -0.09 |
| CaOWs | -0.28 | -0.09 | -0.11 | -0.07 | -0.11 | -0.11 | -0.06 | -0.18 | 0.01 | -0.03 | -0.04 | -0.19 | -0.09 | -0.04 | 0.28 | -0.06 | -0.15 |
| HydrlCondWs | 0.09 | 0.29 | 0.29 | 0.28 | 0.30 | 0.06 | 0.20 | 0.17 | -0.03 | 0.00 | -0.14 | 0.13 | 0.04 | 0.07 | -0.14 | 0.05 | 0.13 |
| CompStrgthWs | -0.06 | -0.08 | -0.12 | -0.07 | -0.14 | -0.11 | -0.10 | -0.30 | -0.02 | -0.08 | 0.20 | -0.22 | -0.05 | -0.43 | 0.27 | -0.17 | -0.40 |
| WetIndexWs | 0.32 | 0.42 | 0.49 | 0.37 | 0.47 | 0.12 | 0.19 | 0.24 | 0.00 | 0.06 | -0.59 | 0.17 | -0.13 | 0.65 | 0.09 | 0.26 | 0.59 |
| BFIWs | 0.39 | 0.39 | 0.42 | 0.36 | 0.41 | 0.02 | 0.00 | 0.02 | -0.03 | 0.03 | -0.36 | 0.02 | -0.16 | 0.48 | 0.21 | 0.11 | 0.27 |
| PrecipWs_amj | 0.45 | -0.08 | -0.07 | -0.08 | -0.08 | 0.06 | -0.01 | -0.14 | 0.02 | -0.01 | 0.12 | -0.07 | -0.05 | -0.10 | -0.03 | -0.02 | -0.01 |
| PrecipWs_jas | 0.72 | 0.13 | 0.17 | 0.09 | 0.16 | 0.17 | 0.13 | 0.29 | -0.01 | 0.01 | -0.13 | 0.23 | 0.10 | 0.20 | -0.25 | 0.13 | 0.18 |
| PrecipWs_jfm | 0.84 | -0.11 | -0.07 | -0.17 | -0.10 | 0.14 | 0.03 | 0.05 | 0.08 | 0.01 | 0.00 | 0.10 | -0.07 | 0.19 | -0.17 | 0.10 | 0.16 |
| PrecipWs_ond | 1.00 | 0.09 | 0.15 | 0.01 | 0.09 | 0.18 | 0.08 | 0.09 | 0.03 | 0.02 | -0.13 | 0.13 | -0.07 | 0.24 | -0.10 | 0.12 | 0.20 |
| TempWs_amj | 0.09 | 1.00 | 0.99 | 0.99 | 0.99 | 0.02 | 0.29 | 0.28 | -0.10 | 0.06 | -0.49 | 0.11 | -0.10 | 0.32 | 0.13 | 0.20 | 0.30 |
| TempWs_jas | 0.15 | 0.99 | 1.00 | 0.96 | 0.98 | 0.04 | 0.30 | 0.28 | -0.11 | 0.06 | -0.55 | 0.11 | -0.11 | 0.38 | 0.16 | 0.21 | 0.33 |
| TempWs_jfm | 0.01 | 0.99 | 0.96 | 1.00 | 0.99 | 0.00 | 0.26 | 0.29 | -0.11 | 0.06 | -0.44 | 0.13 | -0.07 | 0.28 | 0.09 | 0.18 | 0.29 |
| TempWs_ond | 0.09 | 0.99 | 0.98 | 0.99 | 1.00 | 0.02 | 0.28 | 0.30 | -0.11 | 0.06 | -0.51 | 0.15 | -0.07 | 0.37 | 0.10 | 0.21 | 0.34 |
| Water | 0.18 | 0.02 | 0.04 | 0.00 | 0.02 | 1.00 | 0.05 | 0.06 | 0.11 | 0.05 | -0.07 | 0.03 | -0.03 | 0.02 | -0.04 | 0.21 | 0.01 |
| Developed | 0.08 | 0.29 | 0.30 | 0.26 | 0.28 | 0.05 | 1.00 | 0.07 | 0.00 | -0.01 | -0.39 | -0.17 | -0.19 | -0.09 | -0.16 | 0.03 | -0.02 |
| MechDist | 0.09 | 0.28 | 0.28 | 0.29 | 0.30 | 0.06 | 0.07 | 1.00 | 0.03 | 0.02 | -0.09 | 0.24 | 0.11 | 0.04 | -0.25 | 0.11 | 0.21 |
| Mining | 0.03 | -0.10 | -0.11 | -0.11 | -0.11 | 0.11 | 0.00 | 0.03 | 1.00 | -0.01 | 0.07 | -0.03 | -0.05 | -0.08 | -0.12 | 0.04 | -0.04 |
| Barren | 0.02 | 0.06 | 0.06 | 0.06 | 0.06 | 0.05 | -0.01 | 0.02 | -0.01 | 1.00 | -0.01 | 0.02 | 0.02 | 0.04 | -0.04 | 0.05 | 0.07 |
| Forest_Deciduous | -0.13 | -0.49 | -0.55 | -0.44 | -0.51 | -0.07 | -0.39 | -0.09 | 0.07 | -0.01 | 1.00 | -0.10 | -0.03 | -0.50 | -0.55 | -0.17 | -0.27 |
| Forest_Evergreen | 0.13 | 0.11 | 0.11 | 0.13 | 0.15 | 0.03 | -0.17 | 0.24 | -0.03 | 0.02 | -0.10 | 1.00 | 0.33 | 0.01 | -0.28 | 0.10 | 0.12 |
| Forest_Mixed | -0.07 | -0.10 | -0.11 | -0.07 | -0.07 | -0.03 | -0.19 | 0.11 | -0.05 | 0.02 | -0.03 | 0.33 | 1.00 | -0.22 | -0.28 | -0.07 | -0.07 |
| Cropland | 0.24 | 0.32 | 0.38 | 0.28 | 0.37 | 0.02 | -0.09 | 0.04 | -0.08 | 0.04 | -0.50 | 0.01 | -0.22 | 1.00 | 0.19 | 0.16 | 0.42 |
| Hay_Pasture | -0.10 | 0.13 | 0.16 | 0.09 | 0.10 | -0.04 | -0.16 | -0.25 | -0.12 | -0.04 | -0.55 | -0.28 | -0.28 | 0.19 | 1.00 | -0.02 | -0.10 |
| Wetland_Herbaceous | 0.12 | 0.20 | 0.21 | 0.18 | 0.21 | 0.21 | 0.03 | 0.11 | 0.04 | 0.05 | -0.17 | 0.10 | -0.07 | 0.16 | -0.02 | 1.00 | 0.29 |
| Wetland_Woody | 0.20 | 0.30 | 0.33 | 0.29 | 0.34 | 0.01 | -0.02 | 0.21 | -0.04 | 0.07 | -0.27 | 0.12 | -0.07 | 0.42 | -0.10 | 0.29 | 1.00 |

Table S4. Total stream kilometers from NHDPlusV2 and overall percentages predicted to be in Poor, Fair, or Good condition for each land-use and climate projection. Land-use projection abbreviations listed in Table S1.

|  |  |  | Stream Kilometers | | |  | Percent | | |
| --- | --- | --- | --- | --- | --- | --- | --- | --- | --- |
| Land Use | Climate Model | Year | Good | Fair | Poor |  | Good | Fair | Poor |
| Baseline predictions | |  |  |  |  |  |  |  |  |
| Baseline | Baseline | 2005 | 42921 | 33226 | 38405 |  | 37.5 | 29.0 | 33.5 |
| Land use only projections | | |  |  |  |  |  |  |  |
| A1B | Baseline | 2030 | 41440 | 33213 | 39900 |  | 36.2 | 29.0 | 34.8 |
| A1B | Baseline | 2060 | 39927 | 33008 | 41617 |  | 34.9 | 28.8 | 36.3 |
| A1B | Baseline | 2090 | 38635 | 33300 | 42617 |  | 33.7 | 29.1 | 37.2 |
| A2 | Baseline | 2030 | 40779 | 33572 | 40201 |  | 35.6 | 29.3 | 35.1 |
| A2 | Baseline | 2060 | 39040 | 33505 | 42007 |  | 34.1 | 29.3 | 36.7 |
| A2 | Baseline | 2090 | 34791 | 35636 | 44125 |  | 30.4 | 31.1 | 38.5 |
| B1 | Baseline | 2030 | 42333 | 33519 | 38700 |  | 37.0 | 29.3 | 33.8 |
| B1 | Baseline | 2060 | 41992 | 33203 | 39357 |  | 36.7 | 29.0 | 34.4 |
| B1 | Baseline | 2090 | 41615 | 32799 | 40138 |  | 36.3 | 28.6 | 35.0 |
| B2 | Baseline | 2030 | 42175 | 33445 | 38933 |  | 36.8 | 29.2 | 34.0 |
| B2 | Baseline | 2060 | 42577 | 33618 | 38358 |  | 37.2 | 29.4 | 33.5 |
| B2 | Baseline | 2090 | 42579 | 33665 | 38308 |  | 37.2 | 29.4 | 33.4 |
| Climate only projections | |  |  |  |  |  |  |  |  |
| Baseline | CMIP5 p25 | 2030 | 35395 | 37541 | 41615 |  | 30.9 | 32.8 | 36.3 |
| Baseline | CMIP5 p25 | 2060 | 34701 | 39063 | 40788 |  | 30.3 | 34.1 | 35.6 |
| Baseline | CMIP5 p25 | 2090 | 34515 | 39520 | 40517 |  | 30.1 | 34.5 | 35.4 |
| Baseline | CMIP5 p50 | 2030 | 36367 | 37340 | 40845 |  | 31.8 | 32.6 | 35.7 |
| Baseline | CMIP5 p50 | 2060 | 35952 | 38552 | 40049 |  | 31.4 | 33.7 | 35.0 |
| Baseline | CMIP5 p50 | 2090 | 35930 | 38689 | 39933 |  | 31.4 | 33.8 | 34.9 |
| Baseline | CMIP5 p75 | 2030 | 37349 | 37258 | 39945 |  | 32.6 | 32.5 | 34.9 |
| Baseline | CMIP5 p75 | 2060 | 37078 | 38019 | 39456 |  | 32.4 | 33.2 | 34.4 |
| Baseline | CMIP5 p75 | 2090 | 37218 | 37987 | 39347 |  | 32.5 | 33.2 | 34.4 |
| Baseline | Lynch2016 | 2030 | 42952 | 33145 | 38455 |  | 37.5 | 28.9 | 33.6 |
| Baseline | Lynch2016 | 2060 | 42175 | 34012 | 38364 |  | 36.8 | 29.7 | 33.5 |
| Baseline | Lynch2016 | 2090 | 41850 | 34096 | 38606 |  | 36.5 | 29.8 | 33.7 |
| Coupled land use and climate projections | | |  |  |  |  |  |  |  |
| A1B | CMIP5 p25 | 2030 | 34024 | 38211 | 42317 |  | 29.7 | 33.4 | 36.9 |
| A1B | CMIP5 p25 | 2060 | 32110 | 40487 | 41956 |  | 28.0 | 35.3 | 36.6 |
| A1B | CMIP5 p25 | 2090 | 30762 | 41476 | 42313 |  | 26.9 | 36.2 | 36.9 |
| A1B | CMIP5 p50 | 2030 | 35028 | 38012 | 41512 |  | 30.6 | 33.2 | 36.2 |
| A1B | CMIP5 p50 | 2060 | 33361 | 39891 | 41300 |  | 29.1 | 34.8 | 36.1 |
| A1B | CMIP5 p50 | 2090 | 32224 | 40902 | 41426 |  | 28.1 | 35.7 | 36.2 |
| A1B | CMIP5 p75 | 2030 | 36081 | 38011 | 40460 |  | 31.5 | 33.2 | 35.3 |
| A1B | CMIP5 p75 | 2060 | 34512 | 39437 | 40603 |  | 30.1 | 34.4 | 35.5 |
| A1B | CMIP5 p75 | 2090 | 33576 | 40303 | 40674 |  | 29.3 | 35.2 | 35.5 |
| A1B | Lynch2016 | 2030 | 41663 | 33608 | 39281 |  | 36.4 | 29.3 | 34.3 |
| A1B | Lynch2016 | 2060 | 39623 | 35186 | 39744 |  | 34.6 | 30.7 | 34.7 |
| A1B | Lynch2016 | 2090 | 38450 | 35952 | 40151 |  | 33.6 | 31.4 | 35.1 |
| A2 | CMIP5 p25 | 2030 | 33293 | 38707 | 42552 |  | 29.1 | 33.8 | 37.2 |
| A2 | CMIP5 p25 | 2060 | 31044 | 41139 | 42369 |  | 27.1 | 35.9 | 37.0 |
| A2 | CMIP5 p25 | 2090 | 27577 | 43610 | 43365 |  | 24.1 | 38.1 | 37.9 |
| A2 | CMIP5 p50 | 2030 | 34356 | 38462 | 41733 |  | 30.0 | 33.6 | 36.4 |
| A2 | CMIP5 p50 | 2060 | 32520 | 40475 | 41558 |  | 28.4 | 35.3 | 36.3 |
| A2 | CMIP5 p50 | 2090 | 29349 | 42799 | 42404 |  | 25.6 | 37.4 | 37.0 |
| A2 | CMIP5 p75 | 2030 | 35425 | 38387 | 40740 |  | 30.9 | 33.5 | 35.6 |
| A2 | CMIP5 p75 | 2060 | 33731 | 39966 | 40854 |  | 29.5 | 34.9 | 35.7 |
| A2 | CMIP5 p75 | 2090 | 30692 | 42191 | 41669 |  | 26.8 | 36.8 | 36.4 |
| A2 | Lynch2016 | 2030 | 41241 | 33921 | 39390 |  | 36.0 | 29.6 | 34.4 |
| A2 | Lynch2016 | 2060 | 39015 | 35602 | 39935 |  | 34.1 | 31.1 | 34.9 |
| A2 | Lynch2016 | 2090 | 35092 | 38369 | 41091 |  | 30.6 | 33.5 | 35.9 |
| B1 | CMIP5 p25 | 2030 | 35186 | 37773 | 41593 |  | 30.7 | 33.0 | 36.3 |
| B1 | CMIP5 p25 | 2060 | 34276 | 39475 | 40802 |  | 29.9 | 34.5 | 35.6 |
| B1 | CMIP5 p25 | 2090 | 33836 | 39958 | 40758 |  | 29.5 | 34.9 | 35.6 |
| B1 | CMIP5 p50 | 2030 | 36095 | 37707 | 40751 |  | 31.5 | 32.9 | 35.6 |
| B1 | CMIP5 p50 | 2060 | 35398 | 39116 | 40039 |  | 30.9 | 34.2 | 35.0 |
| B1 | CMIP5 p50 | 2090 | 35200 | 39341 | 40011 |  | 30.7 | 34.3 | 34.9 |
| B1 | CMIP5 p75 | 2030 | 37109 | 37645 | 39799 |  | 32.4 | 32.9 | 34.7 |
| B1 | CMIP5 p75 | 2060 | 36544 | 38563 | 39445 |  | 31.9 | 33.7 | 34.4 |
| B1 | CMIP5 p75 | 2090 | 36457 | 38776 | 39319 |  | 31.8 | 33.9 | 34.3 |
| B1 | Lynch2016 | 2030 | 42448 | 33484 | 38620 |  | 37.1 | 29.2 | 33.7 |
| B1 | Lynch2016 | 2060 | 41488 | 34627 | 38438 |  | 36.2 | 30.2 | 33.6 |
| B1 | Lynch2016 | 2090 | 40922 | 35002 | 38628 |  | 35.7 | 30.6 | 33.7 |
| B2 | CMIP5 p25 | 2030 | 34967 | 37776 | 41809 |  | 30.5 | 33.0 | 36.5 |
| B2 | CMIP5 p25 | 2060 | 34679 | 39408 | 40464 |  | 30.3 | 34.4 | 35.3 |
| B2 | CMIP5 p25 | 2090 | 34538 | 39731 | 40283 |  | 30.2 | 34.7 | 35.2 |
| B2 | CMIP5 p50 | 2030 | 35820 | 37774 | 40958 |  | 31.3 | 33.0 | 35.8 |
| B2 | CMIP5 p50 | 2060 | 35977 | 38851 | 39723 |  | 31.4 | 33.9 | 34.7 |
| B2 | CMIP5 p50 | 2090 | 35679 | 39339 | 39534 |  | 31.2 | 34.3 | 34.5 |
| B2 | CMIP5 p75 | 2030 | 36808 | 37756 | 39988 |  | 32.1 | 33.0 | 34.9 |
| B2 | CMIP5 p75 | 2060 | 36863 | 38508 | 39181 |  | 32.2 | 33.6 | 34.2 |
| B2 | CMIP5 p75 | 2090 | 36865 | 38778 | 38909 |  | 32.2 | 33.9 | 34.0 |
| B2 | Lynch2016 | 2030 | 42349 | 33569 | 38634 |  | 37.0 | 29.3 | 33.7 |
| B2 | Lynch2016 | 2060 | 41662 | 34745 | 38145 |  | 36.4 | 30.3 | 33.3 |
| B2 | Lynch2016 | 2090 | 41315 | 34917 | 38321 |  | 36.1 | 30.5 | 33.5 |

Table S5. Total stream kilometers from NHDPlusV2 separated by predicted change in stream conditions for each land-use and climate projection. Coding convention under Stream Kilometers: first category indicates predictions from the baseline prediction and the second category indicates predictions from the time-period of future prediction. For example, Good/Good indicates stream kilometers predicted in good condition under both baseline and 2090, Good/Fair indicates stream kilometers with predicted Good conditions under the baseline projection but Fair conditions in 2090. Total Incr. = total stream kilometers predicted with improved categorical condition, Total Decr. = total stream kilometers predicted with degraded categorical condition, Net Change = Total Incr. – Total Decr., Add. Effect = Additive effects calculated as the sum of associated land-use only and climate only Net Changes, and Interaction Outcome relates how overall watershed condition from the combined scenarios related to the additive effect of individual scenarios. Land-use projection abbreviations listed in Table 1.

| Land-use | Climate Model |  | Stream Kilometers | | | | | | | | | | | |  |  |
| --- | --- | --- | --- | --- | --- | --- | --- | --- | --- | --- | --- | --- | --- | --- | --- | --- |
|  |  | Year | Fair/ Fair | Fair/ Good | Fair/ Poor | Good/ Fair | Good/ Good | Good/ Poor | Poor/ Fair | Poor/ Good | Poor/ Poor | Total Incr. | Total Decr. | Net Change | Add. Effect | Interaction Outcome |
| Land-use only projections | | |  |  |  |  |  |  |  |  |  |  |  |  |  |  |
| A1B | Baseline | 2030 | 30764 | 484 | 1978 | 1941 | 40955 | 24 | 508 | 0 | 37898 | 992 | 3943 | -2952 | na | na |
| A1B | Baseline | 2060 | 29146 | 478 | 3602 | 3347 | 39449 | 125 | 515 | 0 | 37890 | 993 | 7074 | -6081 | na | na |
| A1B | Baseline | 2090 | 28293 | 378 | 4555 | 4421 | 38257 | 243 | 586 | 0 | 37820 | 963 | 9218 | -8255 | na | na |
| A2 | Baseline | 2030 | 30481 | 457 | 2288 | 2589 | 40322 | 10 | 503 | 0 | 37903 | 959 | 4887 | -3928 | na | na |
| A2 | Baseline | 2060 | 28560 | 589 | 4077 | 4359 | 38451 | 111 | 586 | 0 | 37819 | 1176 | 8547 | -7372 | na | na |
| A2 | Baseline | 2090 | 26640 | 485 | 6100 | 8268 | 34306 | 347 | 728 | 0 | 37677 | 1213 | 14716 | -13502 | na | na |
| B1 | Baseline | 2030 | 31292 | 793 | 1141 | 1366 | 41540 | 16 | 861 | 0 | 37544 | 1654 | 2522 | -868 | na | na |
| B1 | Baseline | 2060 | 30575 | 867 | 1783 | 1747 | 41124 | 50 | 881 | 0 | 37524 | 1748 | 3580 | -1832 | na | na |
| B1 | Baseline | 2090 | 29906 | 838 | 2482 | 2054 | 40777 | 90 | 839 | 0 | 37566 | 1677 | 4626 | -2949 | na | na |
| B2 | Baseline | 2030 | 31352 | 651 | 1222 | 1383 | 41523 | 15 | 709 | 0 | 37696 | 1361 | 2620 | -1259 | na | na |
| B2 | Baseline | 2060 | 30142 | 1528 | 1555 | 1849 | 41048 | 24 | 1626 | 0 | 36779 | 3154 | 3428 | -274 | na | na |
| B2 | Baseline | 2090 | 29827 | 1721 | 1677 | 2039 | 40853 | 29 | 1799 | 5 | 36601 | 3525 | 3746 | -221 | na | na |
| Climate only projections | | |  |  |  |  |  |  |  |  |  |  |  |  |  |  |
| Baseline | CMIP5 p25 | 2030 | 27500 | 653 | 5073 | 7464 | 34743 | 714 | 2577 | 0 | 35828 | 3230 | 13252 | -10022 | na | na |
| Baseline | CMIP5 p25 | 2060 | 27581 | 713 | 4932 | 8058 | 33988 | 874 | 3424 | 0 | 34981 | 4137 | 13865 | -9728 | na | na |
| Baseline | CMIP5 p25 | 2090 | 27549 | 764 | 4913 | 8256 | 33750 | 915 | 3715 | 1 | 34689 | 4480 | 14085 | -9604 | na | na |
| Baseline | CMIP5 p50 | 2030 | 27718 | 837 | 4670 | 6762 | 35530 | 629 | 2860 | 0 | 35546 | 3697 | 12062 | -8365 | na | na |
| Baseline | CMIP5 p50 | 2060 | 27695 | 981 | 4550 | 7185 | 34964 | 772 | 3671 | 7 | 34727 | 4659 | 12507 | -7848 | na | na |
| Baseline | CMIP5 p50 | 2090 | 27534 | 1030 | 4661 | 7274 | 34883 | 764 | 3880 | 17 | 34508 | 4928 | 12699 | -7771 | na | na |
| Baseline | CMIP5 p75 | 2030 | 27791 | 1124 | 4310 | 6144 | 36221 | 556 | 3322 | 3 | 35079 | 4450 | 11010 | -6560 | na | na |
| Baseline | CMIP5 p75 | 2060 | 27701 | 1199 | 4326 | 6388 | 35861 | 672 | 3930 | 17 | 34458 | 5146 | 11386 | -6239 | na | na |
| Baseline | CMIP5 p75 | 2090 | 27544 | 1225 | 4457 | 6357 | 35976 | 588 | 4086 | 17 | 34302 | 5328 | 11402 | -6074 | na | na |
| Baseline | Lynch2016 | 2030 | 27616 | 2602 | 3008 | 2510 | 40350 | 61 | 3019 | 0 | 35386 | 5621 | 5579 | 42 | na | na |
| Baseline | Lynch2016 | 2060 | 26845 | 3081 | 3301 | 3570 | 39078 | 273 | 3598 | 17 | 34790 | 6696 | 7144 | -449 | na | na |
| Baseline | Lynch2016 | 2090 | 26372 | 3232 | 3622 | 4030 | 38532 | 358 | 3694 | 86 | 34626 | 7011 | 8011 | -1000 | na | na |
| Combined land-use and climate projections | | |  |  |  |  |  |  |  |  |  |  |  |  |  |  |
| A1B | CMIP5 p25 | 2030 | 26933 | 678 | 5615 | 8790 | 33346 | 785 | 2488 | 0 | 35917 | 3166 | 15190 | -12024 | -12973 | less degraded |
| A1B | CMIP5 p25 | 2060 | 26702 | 730 | 5794 | 10501 | 31379 | 1042 | 3284 | 1 | 35120 | 4015 | 17336 | -13321 | -15809 | less degraded |
| A1B | CMIP5 p25 | 2090 | 26353 | 751 | 6122 | 11740 | 30004 | 1178 | 3383 | 8 | 35014 | 4142 | 19039 | -14897 | -17859 | less degraded |
| A1B | CMIP5 p50 | 2030 | 27126 | 883 | 5217 | 8064 | 34145 | 713 | 2823 | 0 | 35582 | 3706 | 13993 | -10287 | -11317 | less degraded |
| A1B | CMIP5 p50 | 2060 | 26936 | 953 | 5336 | 9576 | 32396 | 949 | 3378 | 12 | 35015 | 4343 | 15862 | -11518 | -13929 | less degraded |
| A1B | CMIP5 p50 | 2090 | 26688 | 988 | 5550 | 10658 | 31219 | 1044 | 3556 | 17 | 34833 | 4560 | 17252 | -12691 | -16026 | less degraded |
| A1B | CMIP5 p75 | 2030 | 27332 | 1168 | 4726 | 7354 | 34909 | 658 | 3324 | 5 | 35076 | 4497 | 12738 | -8242 | -9512 | less degraded |
| A1B | CMIP5 p75 | 2060 | 27022 | 1192 | 5011 | 8766 | 33302 | 853 | 3648 | 17 | 34739 | 4858 | 14630 | -9772 | -12320 | less degraded |
| A1B | CMIP5 p75 | 2090 | 26865 | 1162 | 5199 | 9635 | 32395 | 891 | 3803 | 19 | 34584 | 4984 | 15725 | -10742 | -14329 | less degraded |
| A1B | Lynch2016 | 2030 | 27214 | 2411 | 3601 | 3520 | 39244 | 157 | 2874 | 8 | 35523 | 5294 | 7278 | -1985 | -2910 | less degraded |
| A1B | Lynch2016 | 2060 | 26298 | 2671 | 4257 | 5470 | 36925 | 526 | 3418 | 26 | 34961 | 6115 | 10253 | -4137 | -6530 | less degraded |
| A1B | Lynch2016 | 2090 | 25755 | 2781 | 4689 | 6622 | 35583 | 717 | 3575 | 85 | 34745 | 6442 | 12027 | -5586 | -9255 | less degraded |
| A2 | CMIP5 p25 | 2030 | 26818 | 609 | 5798 | 9392 | 32684 | 845 | 2497 | 0 | 35908 | 3107 | 16036 | -12929 | -13949 | less degraded |
| A2 | CMIP5 p25 | 2060 | 26329 | 669 | 6228 | 11469 | 30372 | 1079 | 3341 | 3 | 35062 | 4012 | 18777 | -14765 | -17099 | less degraded |
| A2 | CMIP5 p25 | 2090 | 25576 | 619 | 7031 | 14658 | 26955 | 1308 | 3376 | 3 | 35026 | 3998 | 22997 | -18999 | -23107 | less degraded |
| A2 | CMIP5 p50 | 2030 | 27071 | 777 | 5377 | 8578 | 33579 | 764 | 2813 | 0 | 35592 | 3591 | 14719 | -11129 | -12293 | less degraded |
| A2 | CMIP5 p50 | 2060 | 26612 | 899 | 5716 | 10343 | 31604 | 974 | 3520 | 17 | 34868 | 4435 | 17032 | -12597 | -15220 | less degraded |
| A2 | CMIP5 p50 | 2090 | 25986 | 847 | 6393 | 13289 | 28480 | 1153 | 3524 | 23 | 34858 | 4394 | 20834 | -16441 | -21274 | less degraded |
| A2 | CMIP5 p75 | 2030 | 27289 | 1062 | 4875 | 7836 | 34360 | 725 | 3262 | 3 | 35140 | 4327 | 13436 | -9109 | -10488 | less degraded |
| A2 | CMIP5 p75 | 2060 | 26748 | 1176 | 5302 | 9512 | 32534 | 875 | 3706 | 21 | 34678 | 4903 | 15689 | -10786 | -13611 | less degraded |
| A2 | CMIP5 p75 | 2090 | 26349 | 958 | 5919 | 12181 | 29711 | 1029 | 3661 | 23 | 34721 | 4642 | 19129 | -14487 | -19577 | less degraded |
| A2 | Lynch2016 | 2030 | 27363 | 2215 | 3648 | 3722 | 39017 | 183 | 2837 | 9 | 35559 | 5061 | 7552 | -2491 | -3886 | less degraded |
| A2 | Lynch2016 | 2060 | 26294 | 2544 | 4388 | 5952 | 36427 | 542 | 3356 | 44 | 35005 | 5944 | 10882 | -4938 | -7820 | less degraded |
| A2 | Lynch2016 | 2090 | 25743 | 2065 | 5418 | 9133 | 32960 | 828 | 3493 | 67 | 34845 | 5625 | 15379 | -9753 | -14502 | less degraded |
| B1 | CMIP5 p25 | 2030 | 27079 | 855 | 5291 | 7846 | 34329 | 746 | 2848 | 1 | 35556 | 3705 | 13883 | -10178 | -10890 | less degraded |
| B1 | CMIP5 p25 | 2060 | 27029 | 950 | 5247 | 8623 | 33324 | 973 | 3823 | 1 | 34581 | 4774 | 14844 | -10070 | -11560 | less degraded |
| B1 | CMIP5 p25 | 2090 | 26966 | 930 | 5329 | 9018 | 32891 | 1012 | 3973 | 16 | 34417 | 4919 | 15360 | -10441 | -12553 | less degraded |
| B1 | CMIP5 p50 | 2030 | 27309 | 1052 | 4864 | 7219 | 35041 | 661 | 3179 | 1 | 35225 | 4232 | 12744 | -8512 | -9233 | less degraded |
| B1 | CMIP5 p50 | 2060 | 27263 | 1143 | 4820 | 7820 | 34236 | 866 | 4033 | 19 | 34353 | 5195 | 13506 | -8311 | -9680 | less degraded |
| B1 | CMIP5 p50 | 2090 | 27104 | 1220 | 4902 | 8066 | 33960 | 895 | 4172 | 20 | 34214 | 5412 | 13863 | -8451 | -10721 | less degraded |
| B1 | CMIP5 p75 | 2030 | 27388 | 1333 | 4505 | 6570 | 35755 | 596 | 3687 | 20 | 34698 | 5040 | 11671 | -6630 | -7428 | less degraded |
| B1 | CMIP5 p75 | 2060 | 27313 | 1360 | 4553 | 7014 | 35159 | 748 | 4236 | 25 | 34144 | 5621 | 12315 | -6694 | -8071 | less degraded |
| B1 | CMIP5 p75 | 2090 | 27214 | 1365 | 4647 | 7123 | 35071 | 727 | 4439 | 21 | 33945 | 5825 | 12498 | -6673 | -9023 | less degraded |
| B1 | Lynch2016 | 2030 | 27216 | 2773 | 3236 | 3168 | 39658 | 95 | 3099 | 17 | 35289 | 5890 | 6500 | -610 | -826 | less degraded |
| B1 | Lynch2016 | 2060 | 26388 | 3219 | 3619 | 4377 | 38200 | 345 | 3862 | 69 | 34474 | 7150 | 8340 | -1190 | -2280 | less degraded |
| B1 | Lynch2016 | 2090 | 25934 | 3340 | 3952 | 5001 | 37474 | 446 | 4067 | 108 | 34230 | 7515 | 9399 | -1884 | -3949 | less degraded |
| B2 | CMIP5 p25 | 2030 | 27193 | 768 | 5265 | 7951 | 34196 | 773 | 2631 | 3 | 35771 | 3402 | 13989 | -10587 | -11281 | less degraded |
| B2 | CMIP5 p25 | 2060 | 26980 | 1165 | 5081 | 8398 | 33495 | 1027 | 4030 | 19 | 34356 | 5214 | 14507 | -9292 | -10002 | less degraded |
| B2 | CMIP5 p25 | 2090 | 26885 | 1256 | 5085 | 8601 | 33258 | 1063 | 4246 | 24 | 34135 | 5526 | 14749 | -9223 | -9825 | less degraded |
| B2 | CMIP5 p50 | 2030 | 27443 | 930 | 4852 | 7345 | 34887 | 689 | 2986 | 3 | 35417 | 3919 | 12887 | -8968 | -9624 | less degraded |
| B2 | CMIP5 p50 | 2060 | 27004 | 1534 | 4688 | 7596 | 34417 | 909 | 4251 | 27 | 34127 | 5812 | 13192 | -7381 | -8122 | less degraded |
| B2 | CMIP5 p50 | 2090 | 26889 | 1545 | 4791 | 7913 | 34104 | 905 | 4536 | 30 | 33838 | 6112 | 13609 | -7497 | -7992 | less degraded |
| B2 | CMIP5 p75 | 2030 | 27576 | 1206 | 4443 | 6722 | 35589 | 609 | 3457 | 12 | 34936 | 4676 | 11775 | -7099 | -7820 | less degraded |
| B2 | CMIP5 p75 | 2060 | 27001 | 1721 | 4504 | 7030 | 35110 | 781 | 4477 | 32 | 33896 | 6230 | 12315 | -6085 | -6513 | less degraded |
| B2 | CMIP5 p75 | 2090 | 26849 | 1789 | 4588 | 7134 | 35043 | 744 | 4795 | 33 | 33577 | 6617 | 12467 | -5850 | -6295 | less degraded |
| B2 | Lynch2016 | 2030 | 27444 | 2624 | 3157 | 3129 | 39707 | 85 | 2995 | 18 | 35392 | 5637 | 6371 | -735 | -1217 | less degraded |
| B2 | Lynch2016 | 2060 | 26243 | 3470 | 3513 | 4477 | 38099 | 346 | 4025 | 93 | 34287 | 7588 | 8336 | -747 | -722 | more degraded |
| B2 | Lynch2016 | 2090 | 25689 | 3725 | 3811 | 5005 | 37449 | 467 | 4222 | 140 | 34043 | 8088 | 9283 | -1195 | -1220 | less degraded |

Table S6. Results from the temporal model analyses to test for model consistency at a single region over time. Separate random forests models were built for 1) Chessie BIBI and PRISM precipitation and temperature as defined in the main text data from 2000-2005 using 2001 NLCD data (n = 1,649; 2001 Model) and 2) Chessie BIBI and PRISM precipitation and temperature as defined in the main text data from 2006-2011 using 2006 NLCD data (n = 2,698; 2006 Model). The 2001 model explained 31.2% of variation in raw Chessie BIBI scores in the training data set (Mean of Squared Residuals = 493) correctly classifying 93.5% of poor, 50.5% of fair and 93.9% of good sites for an overall PCC of 83.3 and a kappa statistic = 0.74. The 2006 model explained 38.4% of variation in raw Chessie BIBI scores in the training data set (Mean of Squared Residuals = 459) correctly classifying 94.1% of poor, 53.0% of fair and 94.7% of good sites for an overall PCC of 85.6 and a kappa statistic = 0.77. For test data the 2001 model correctly classifying 72.7% of poor, 20.3% of fair and 66.4% of good sites for an overall PCC of 55.0 and a kappa statistic = 0.31; the 2006 model correctly classifying 77.4% of poor, 20.9% of fair and 81.4% of good sites for an overall PCC of 65.3 and a kappa statistic = 0.46.

|  | 2001 Model | | | | | | | | |  | 2006 Model | | | | | | | | |
| --- | --- | --- | --- | --- | --- | --- | --- | --- | --- | --- | --- | --- | --- | --- | --- | --- | --- | --- | --- |
|  | Training Data | | | |  | Test Data | | | |  | Training Data | | | |  | Test Data | | | |
| Predicted | Poor | Fair | Good | PCC |  | Poor | Fair | Good | PCC |  | Poor | Fair | Good | PCC |  | Poor | Fair | Good | PCC |
| Poor | 431 | 23 | 7 | 93.5 |  | 109 | 12 | 29 | 72.7 |  | 722 | 35 | 10 | 94.1 |  | 188 | 23 | 32 | 77.4 |
| Fair | 83 | 151 | 65 | 50.5 |  | 53 | 25 | 45 | 20.3 |  | 106 | 228 | 96 | 53.0 |  | 67 | 34 | 62 | 20.9 |
| Good | 2 | 27 | 447 | 93.9 |  | 25 | 22 | 93 | 66.4 |  | 1 | 43 | 782 | 94.7 |  | 21 | 29 | 219 | 81.4 |
|  |  |  | Total | 83.3 |  |  |  | Total | 55.0 |  |  |  | Total | 85.6 |  |  |  | Total | 65.3 |

Table S7. Watershed-wide percentages of each land cover under the 2005 baseline and four land-use projections.

| Land Cover | Baseline |  | Economic Growth | | | | | |  | Environmental Sustainability | | | | | |
| --- | --- | --- | --- | --- | --- | --- | --- | --- | --- | --- | --- | --- | --- | --- | --- |
|  |  |  | A1B 2030 | A1B 2060 | A1B 2090 | A2 2030 | A2 2060 | A2 2090 |  | B1 2030 | B1 2060 | B1 2090 | B2 2030 | B2 2060 | B2 2090 |
| Water | 4.9 |  | 4.9 | 4.9 | 4.9 | 4.9 | 4.9 | 4.9 |  | 4.9 | 5.0 | 5.0 | 4.9 | 5.0 | 5.1 |
| Developed | 5.2 |  | 7.6 | 9.2 | 10.7 | 7.0 | 8.7 | 11.3 |  | 6.8 | 7.9 | 8.9 | 6.7 | 7.1 | 7.3 |
| MechDist | 0.8 |  | 1.8 | 2.0 | 2.4 | 1.5 | 1.6 | 1.3 |  | 0.8 | 0.6 | 0.7 | 1.0 | 1.2 | 1.1 |
| Mining | 0.4 |  | 0.5 | 0.6 | 0.6 | 0.5 | 0.6 | 0.7 |  | 0.5 | 0.4 | 0.4 | 0.5 | 0.4 | 0.4 |
| Barren | <0.1 |  | <0.1 | <0.1 | <0.1 | <0.1 | <0.1 | <0.1 |  | <0.1 | <0.1 | <0.1 | <0.1 | <0.1 | <0.1 |
| Forest_Deciduous | 40.5 |  | 38.3 | 35.7 | 33.8 | 36.7 | 33.4 | 25.9 |  | 41.3 | 40.8 | 40.1 | 40.4 | 43.3 | 44.1 |
| Forest_Evergreen | 5.5 |  | 5.2 | 4.5 | 4.1 | 5.0 | 4.4 | 3.2 |  | 5.6 | 5.4 | 5.3 | 5.4 | 5.8 | 5.8 |
| Forest_Mixed | 11.3 |  | 10.5 | 9.5 | 8.7 | 10.2 | 9.1 | 6.9 |  | 11.3 | 11.0 | 10.7 | 11.0 | 11.5 | 11.6 |
| Cropland | 6.1 |  | 6.3 | 7.9 | 8.7 | 7.1 | 8.2 | 11.4 |  | 5.0 | 5.2 | 5.4 | 5.7 | 3.9 | 3.4 |
| Hay_Pasture | 20.2 |  | 19.8 | 20.7 | 21.0 | 22.1 | 24.2 | 29.7 |  | 18.6 | 18.5 | 18.4 | 19.2 | 16.6 | 15.8 |
| Wetland_Herbaceous | 0.9 |  | 0.9 | 0.9 | 0.8 | 0.8 | 0.8 | 0.7 |  | 0.9 | 0.9 | 0.9 | 0.9 | 0.9 | 0.9 |
| Wetland_Woody | 1.5 |  | 1.6 | 1.5 | 1.5 | 1.5 | 1.5 | 1.4 |  | 1.6 | 1.6 | 1.6 | 1.6 | 1.6 | 1.7 |


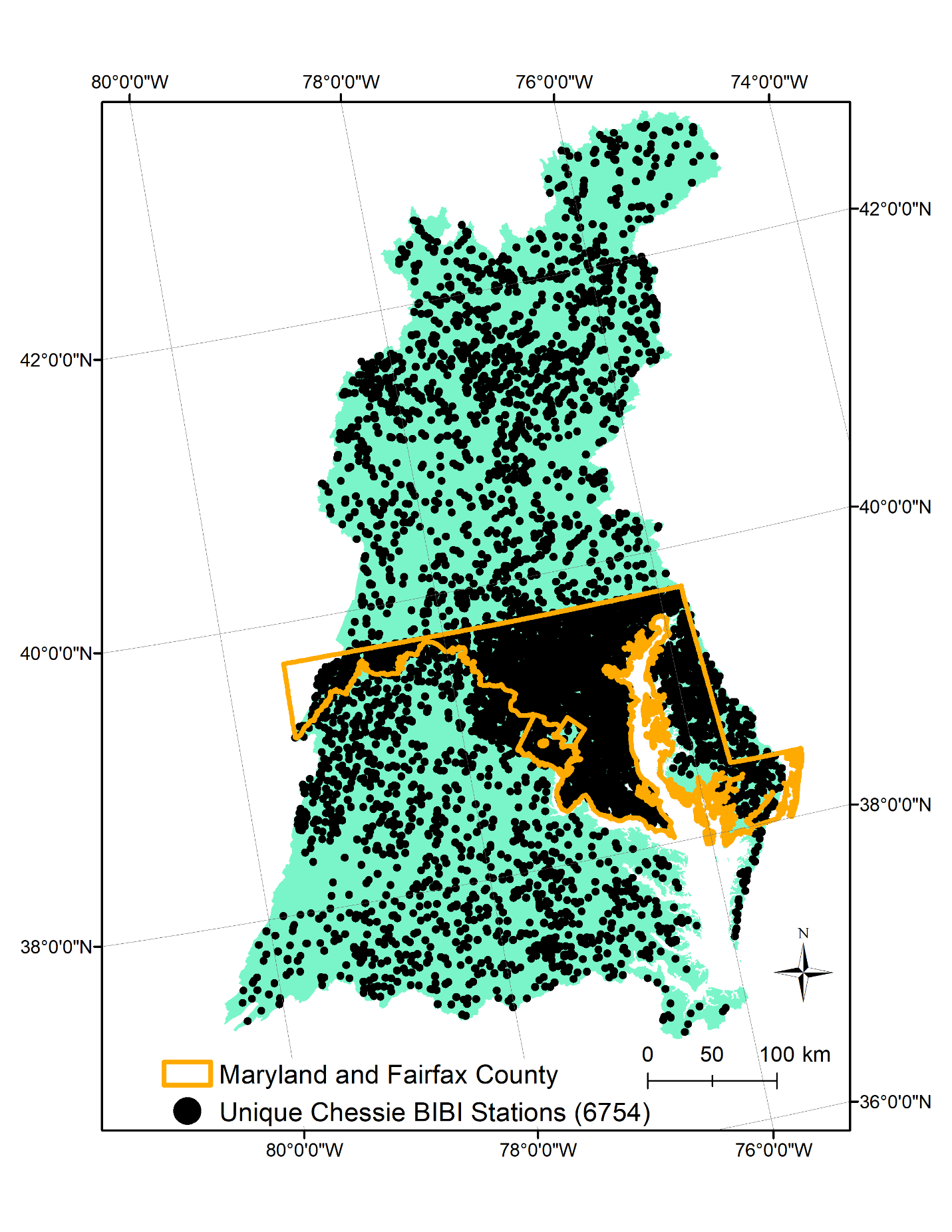
Figure S1. Maps showing all independent Chessie BIBI samples from 01 January 2000– 31 December 2011 (n = 6,754) in the Chesapeake Bay watershed.


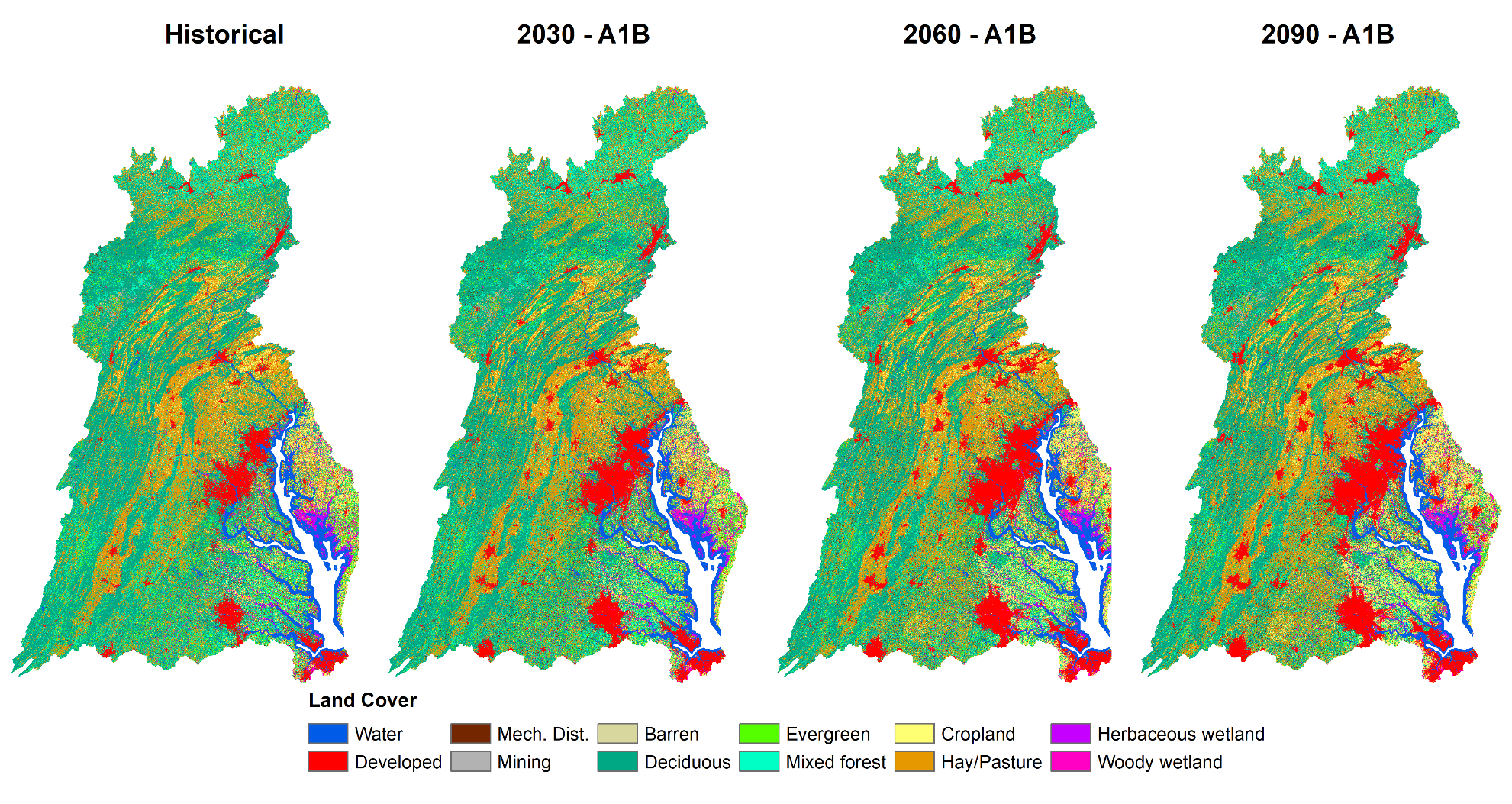


Figure S2. Maps showing A1B land-use projections for baseline, 2030, 2060, and 2090 in the Chesapeake Bay watershed.


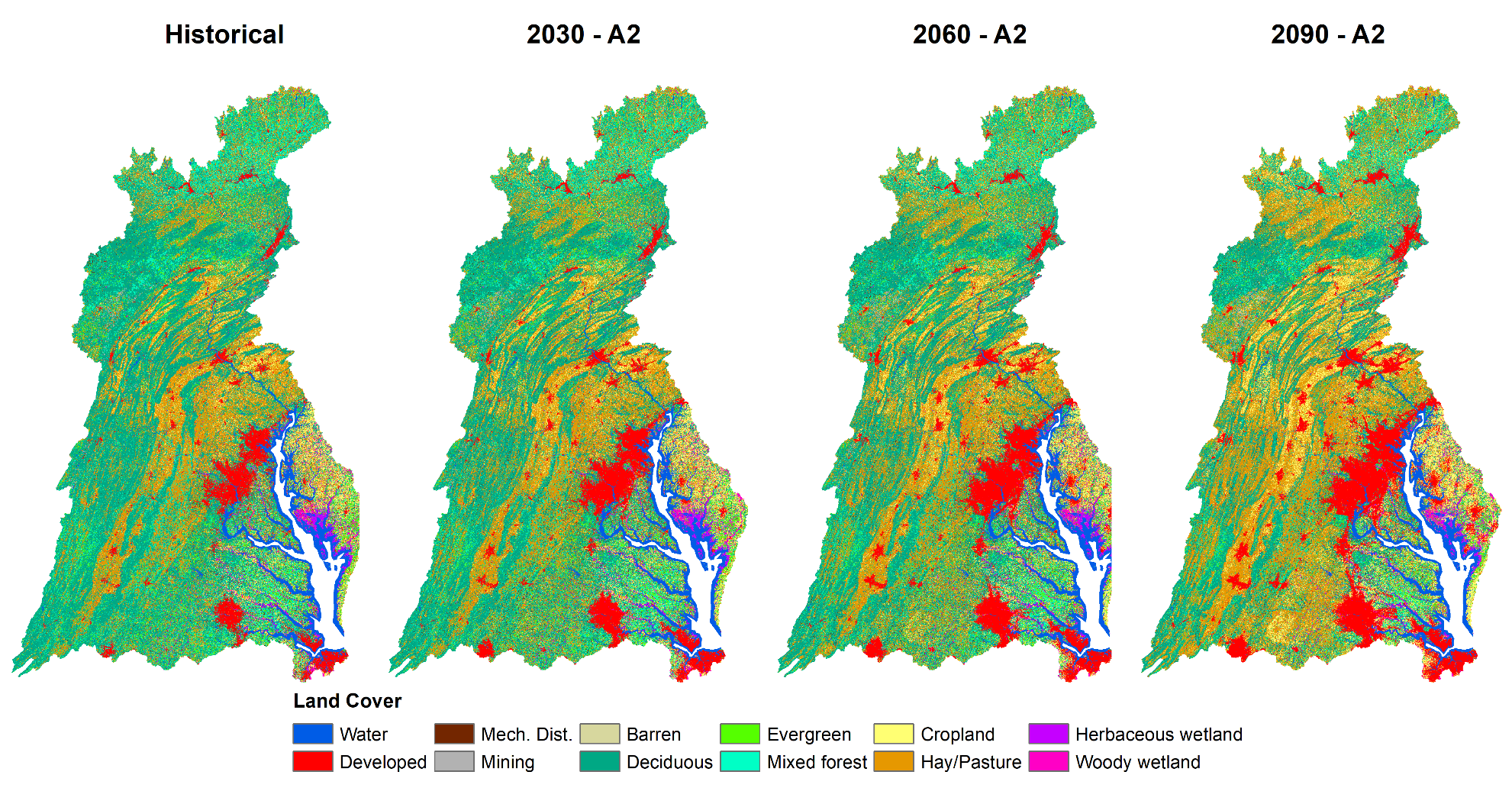


Figure S3. Maps showing A2 land-use projections for baseline, 2030, 2060, and 2090 in the Chesapeake Bay watershed.


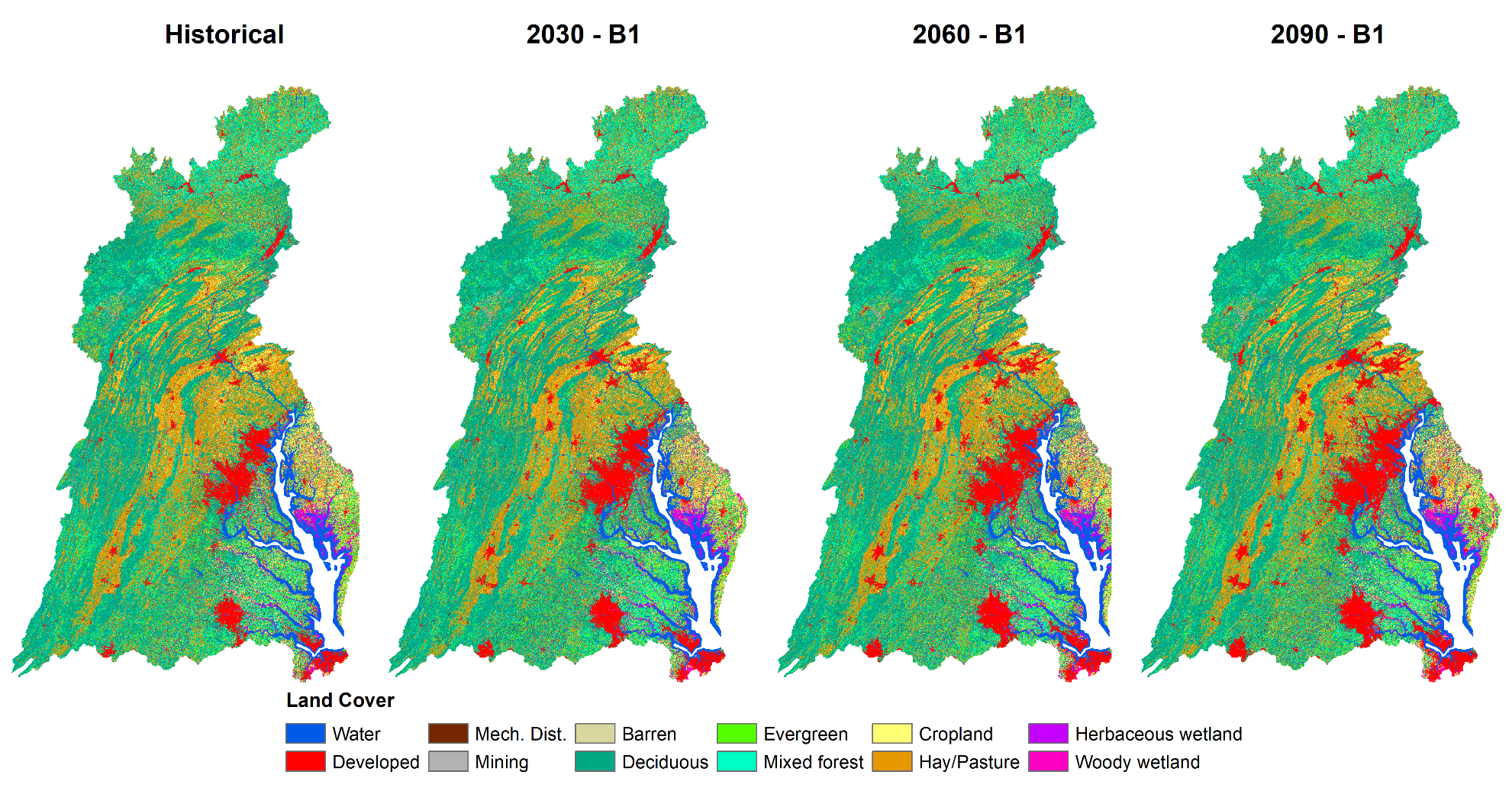


Figure S4. Maps showing B1 land-use projections for baseline, 2030, 2060, and 2090 in the Chesapeake Bay watershed.


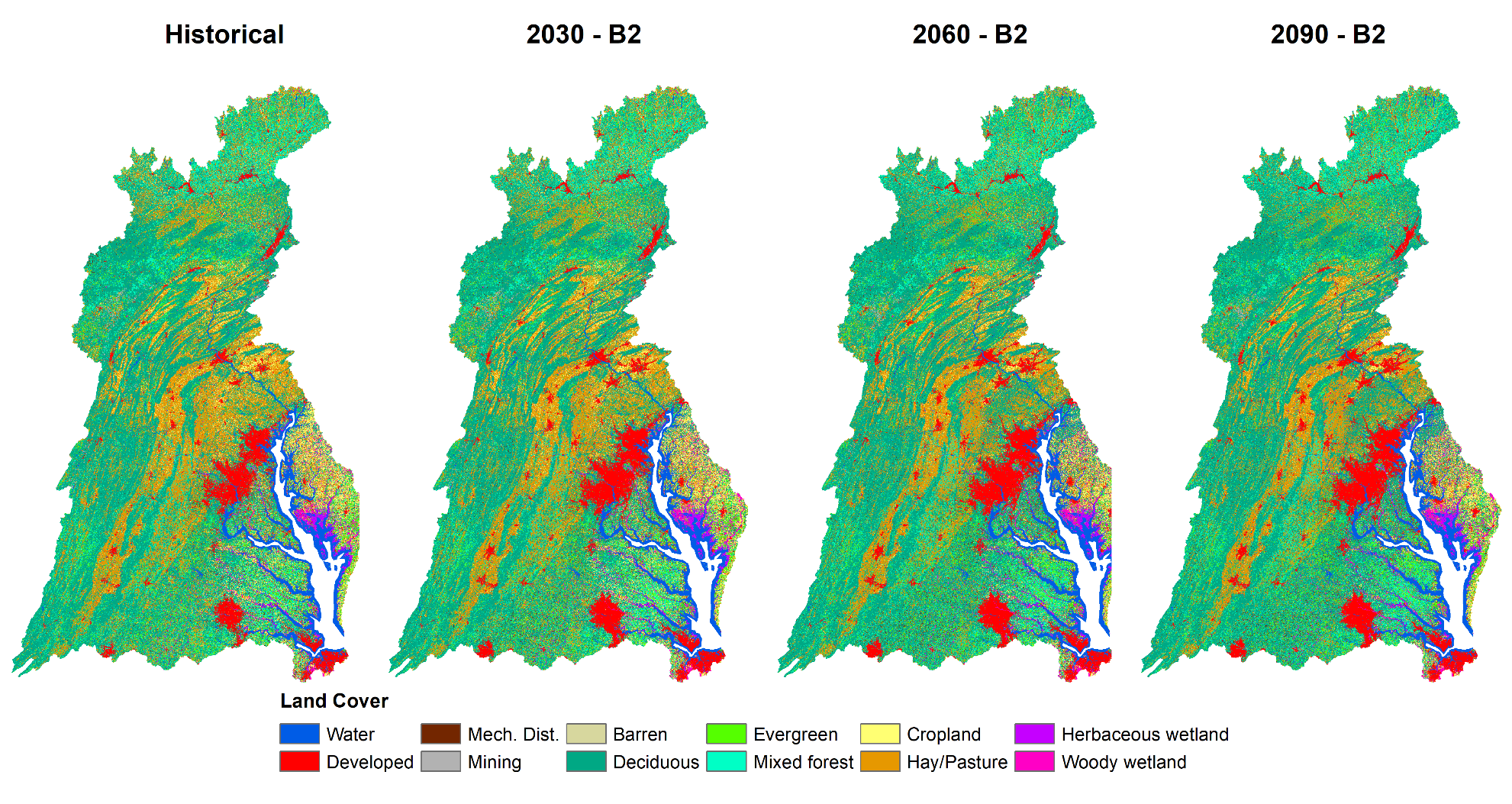


Figure S5. Maps showing B2 land-use projections for baseline, 2030, 2060, and 2090 in the Chesapeake Bay watershed.


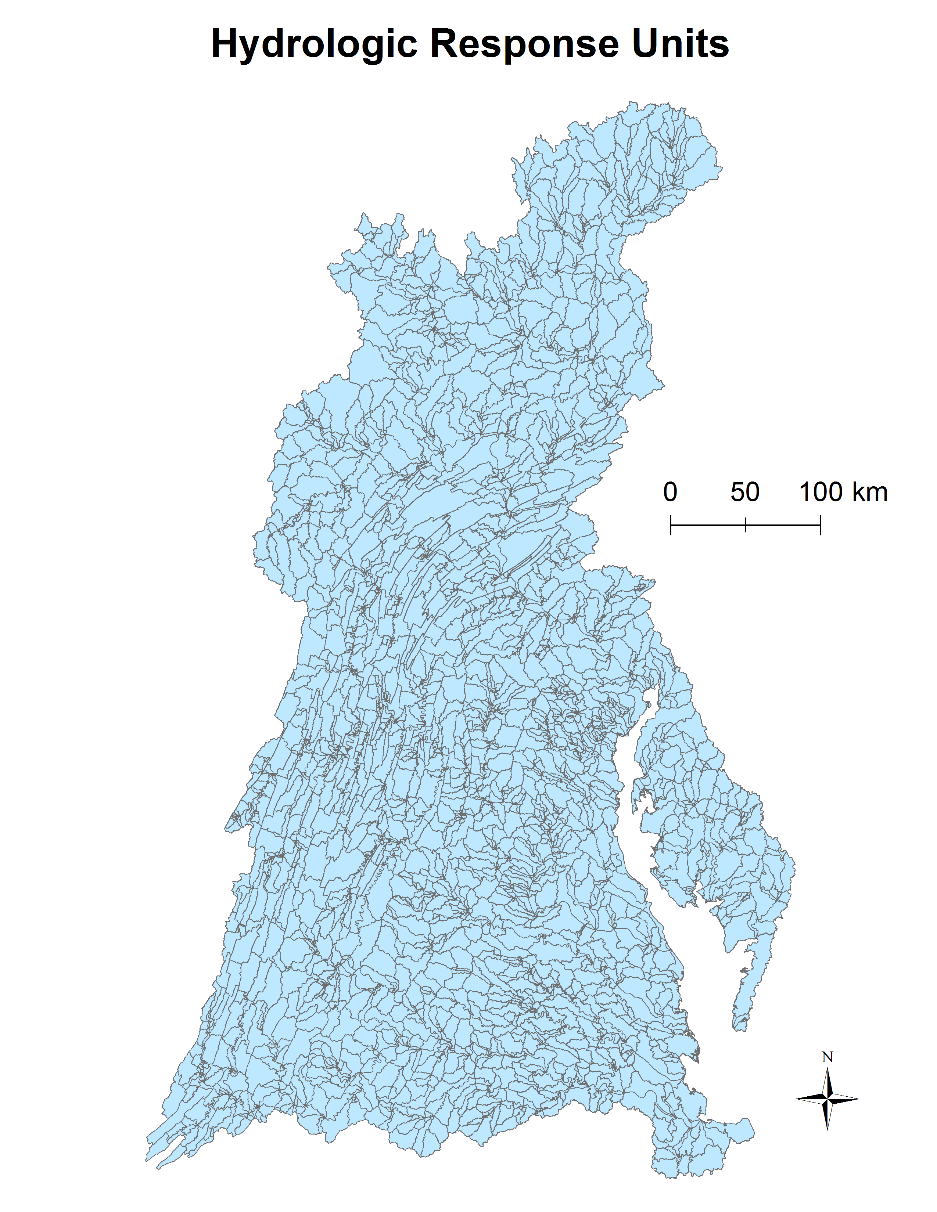


Figure S6. Map showing the hydrological response units in the Chesapeake Bay watershed. In the Chesapeake Bay watershed, HRUs varied in size from approximately 0.05 to 792.99 km^2^.


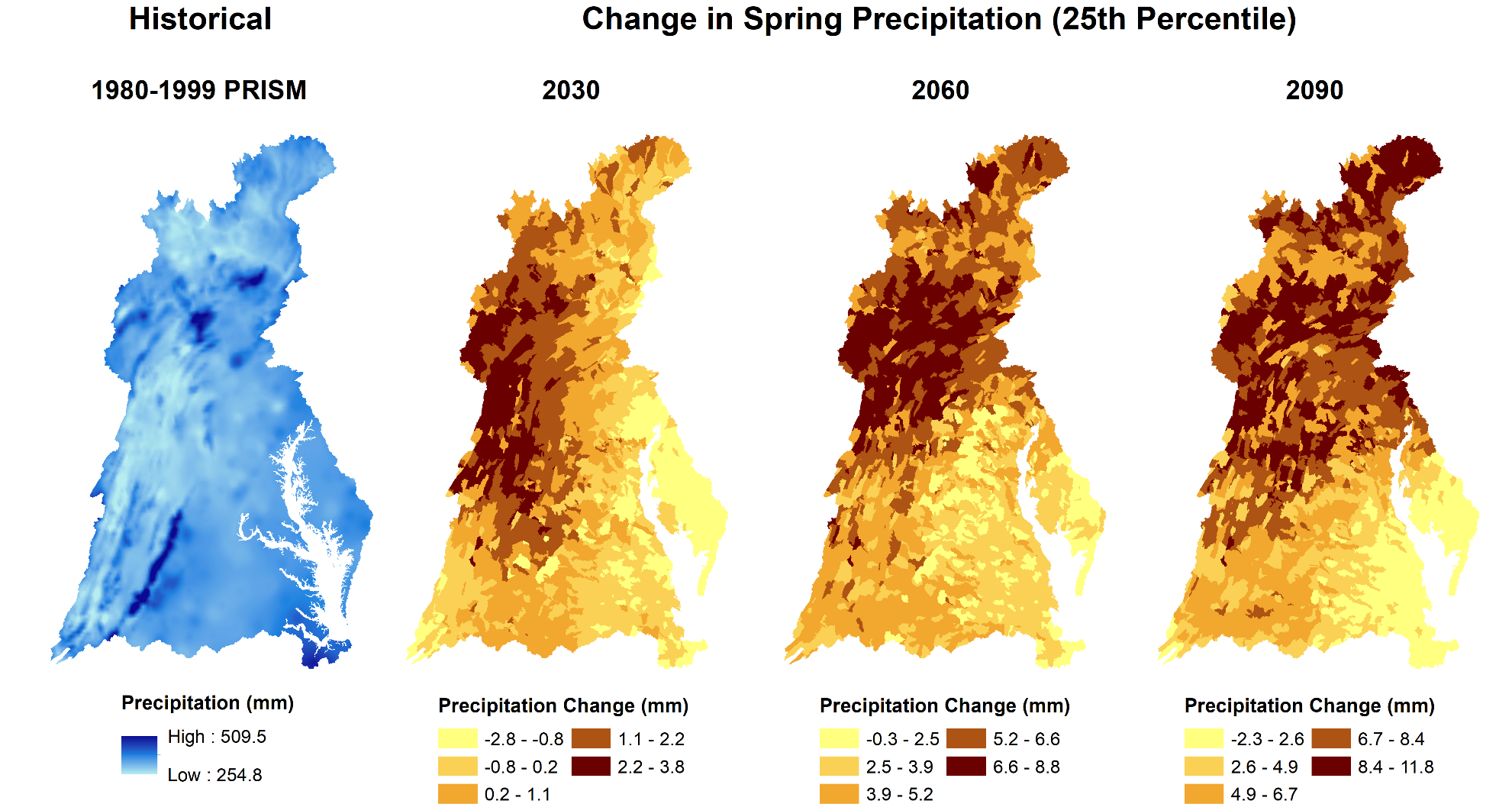


Figure S7. Maps showing CMIP5 p25 spring precipitation projections for historical, 2030, 2060, and 2090 in the Chesapeake Bay watershed.


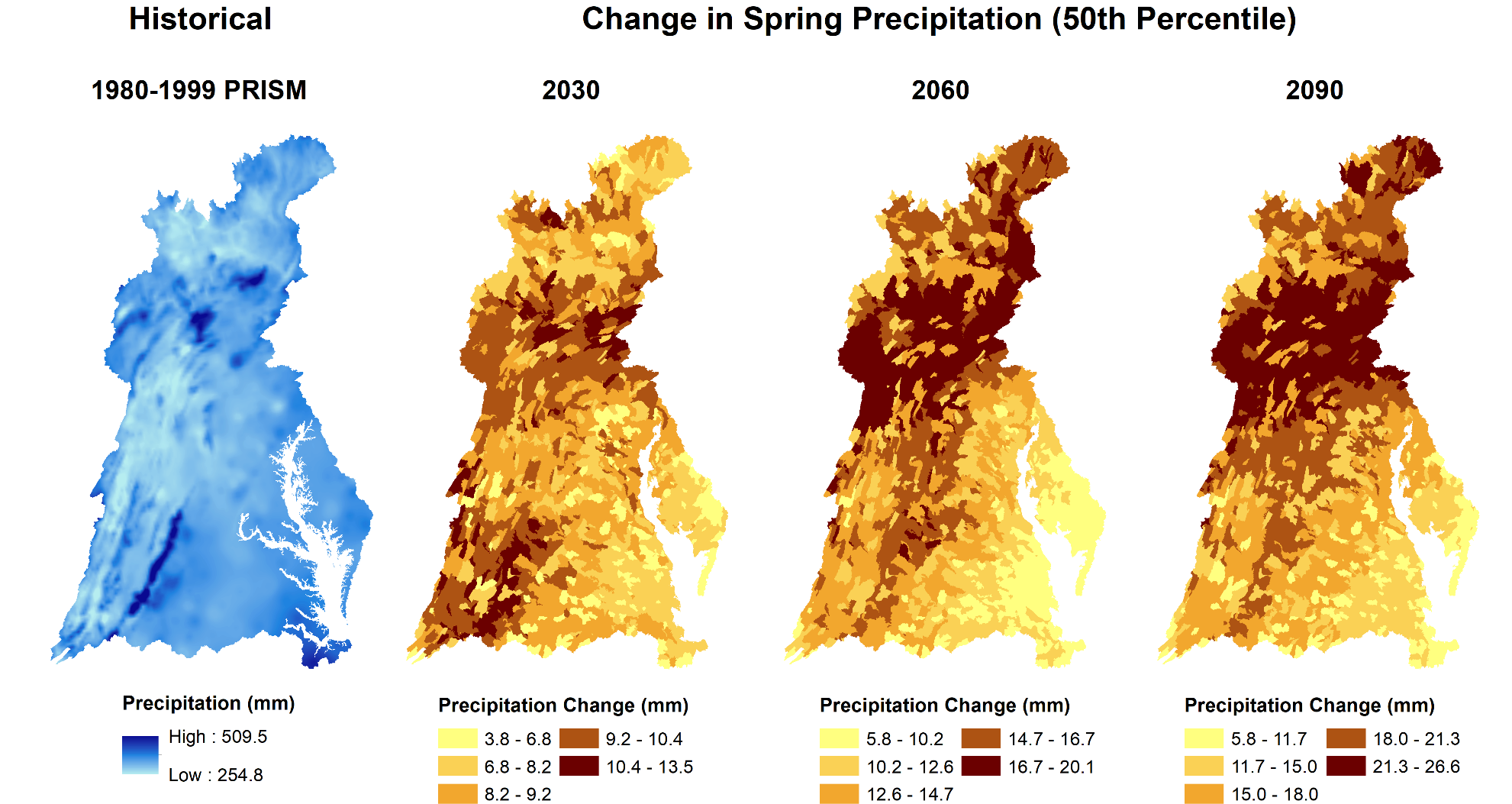


Figure S8. Maps showing CMIP5 p50 spring precipitation projections for historical, 2030, 2060, and 2090 in the Chesapeake Bay watershed.


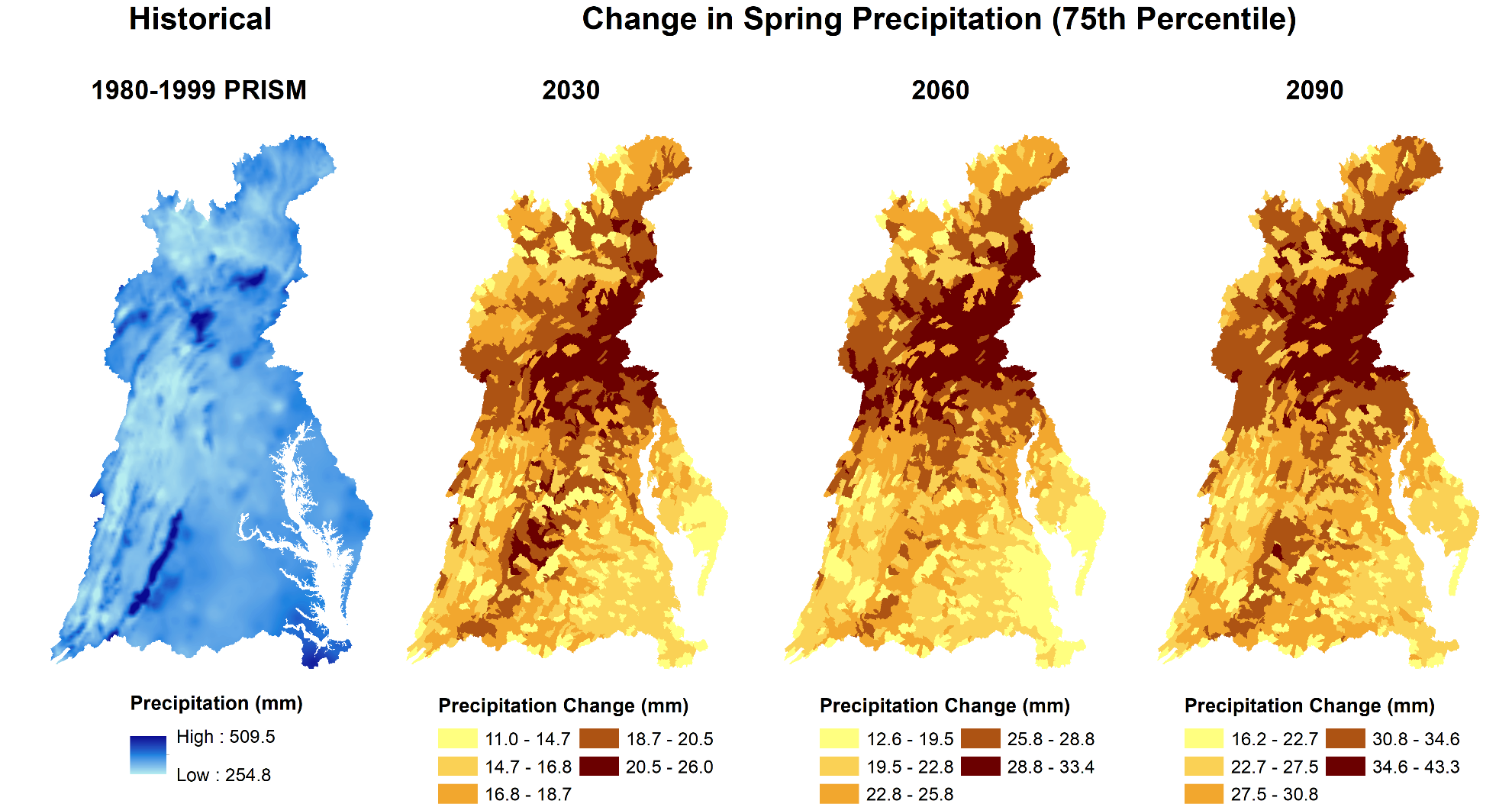


Figure S9. Maps showing CMIP5 p75 spring precipitation projections for historical, 2030, 2060, and 2090 in the Chesapeake Bay watershed.


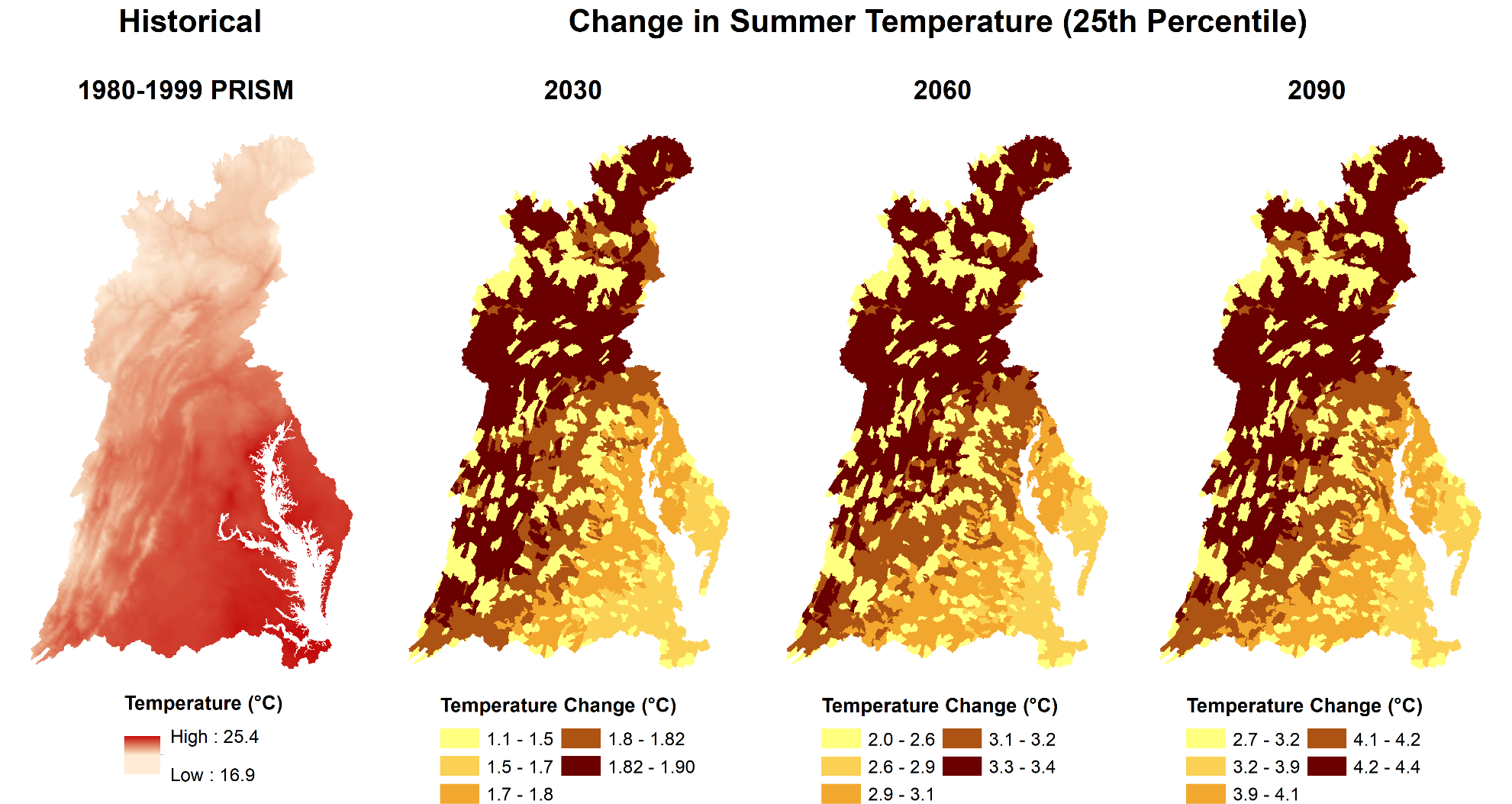


Figure S10. Maps showing CMIP5 p25 summer temperature projections for historical, 2030, 2060, and 2090 in the Chesapeake Bay watershed.


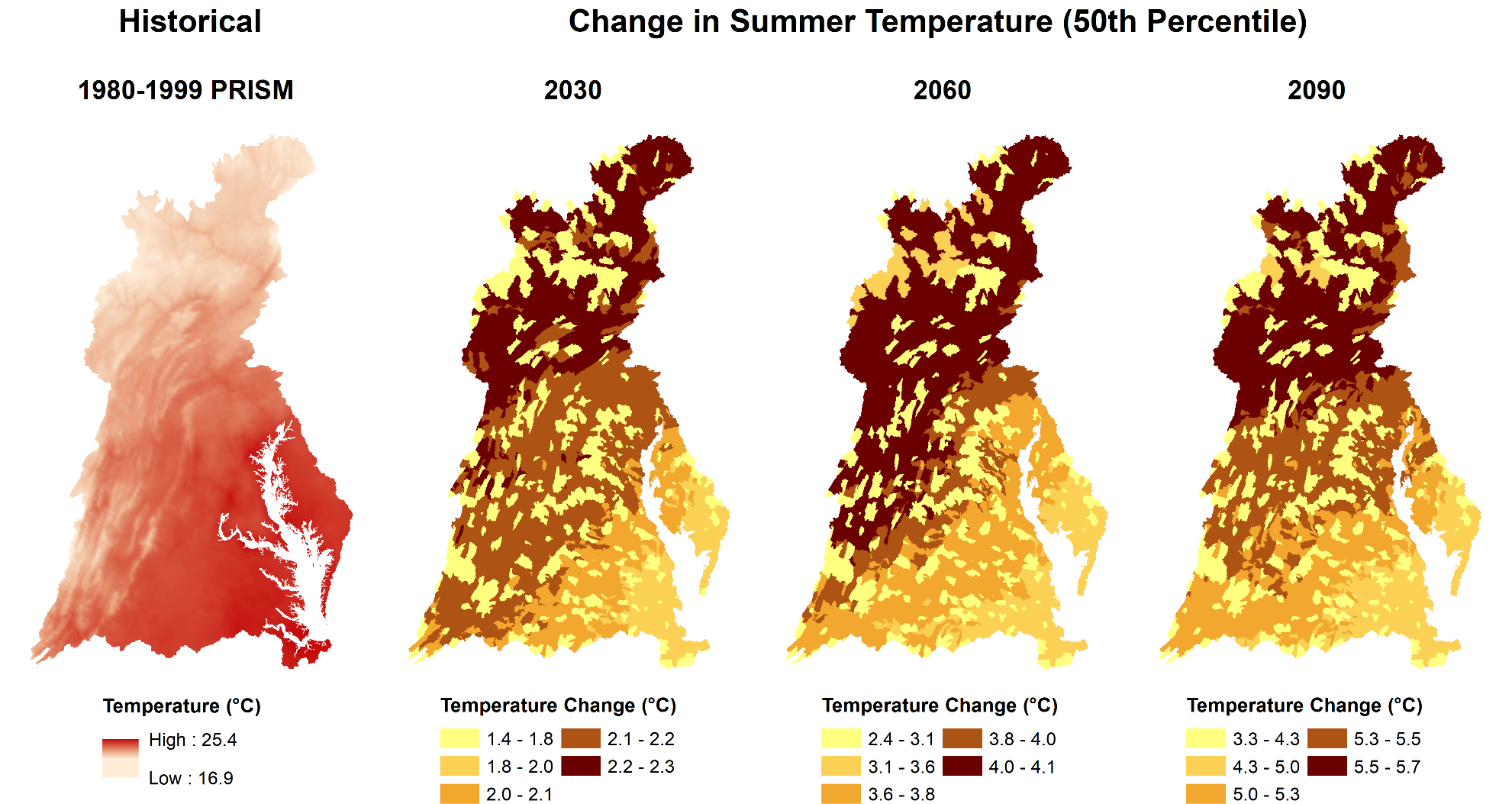


Figure S11. Maps showing CMIP5 p50 summer temperature projections for historical, 2030, 2060, and 2090 in the Chesapeake Bay watershed.


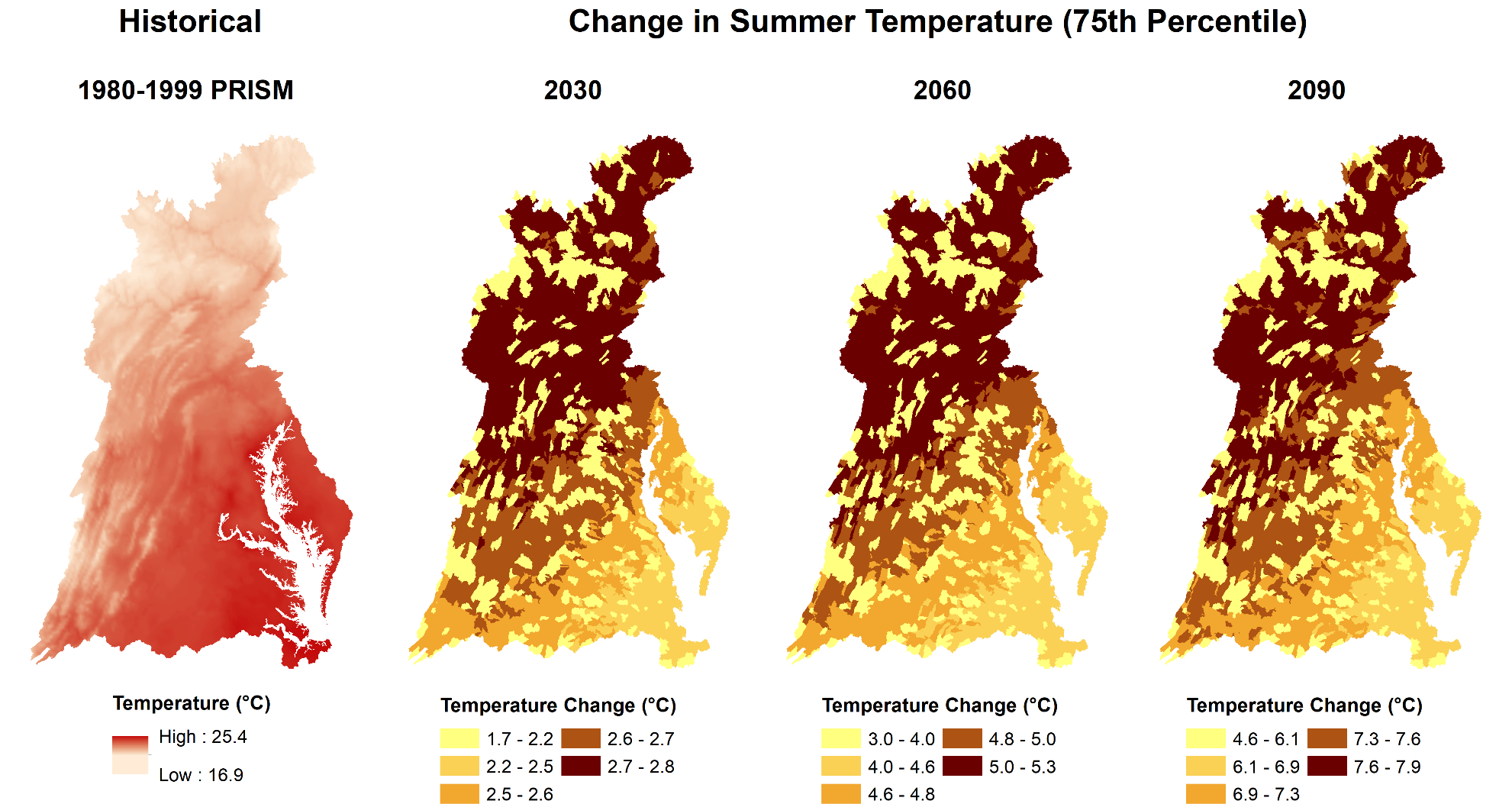


Figure S12. Maps showing CMIP5 p75 summer temperature projections for historical, 2030, 2060, and 2090 in the Chesapeake Bay watershed.


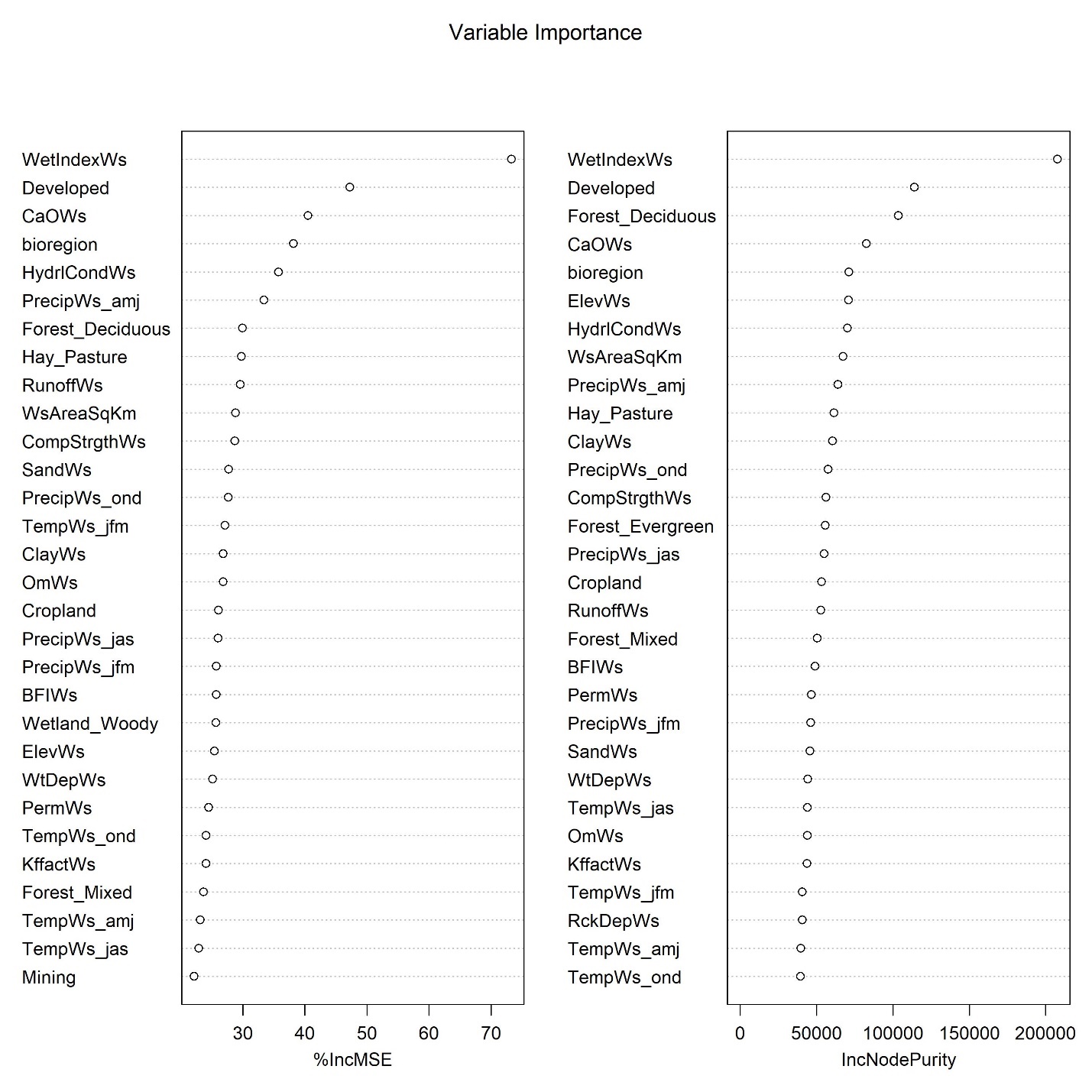


Figure S13. Variable importance plots from baseline model.


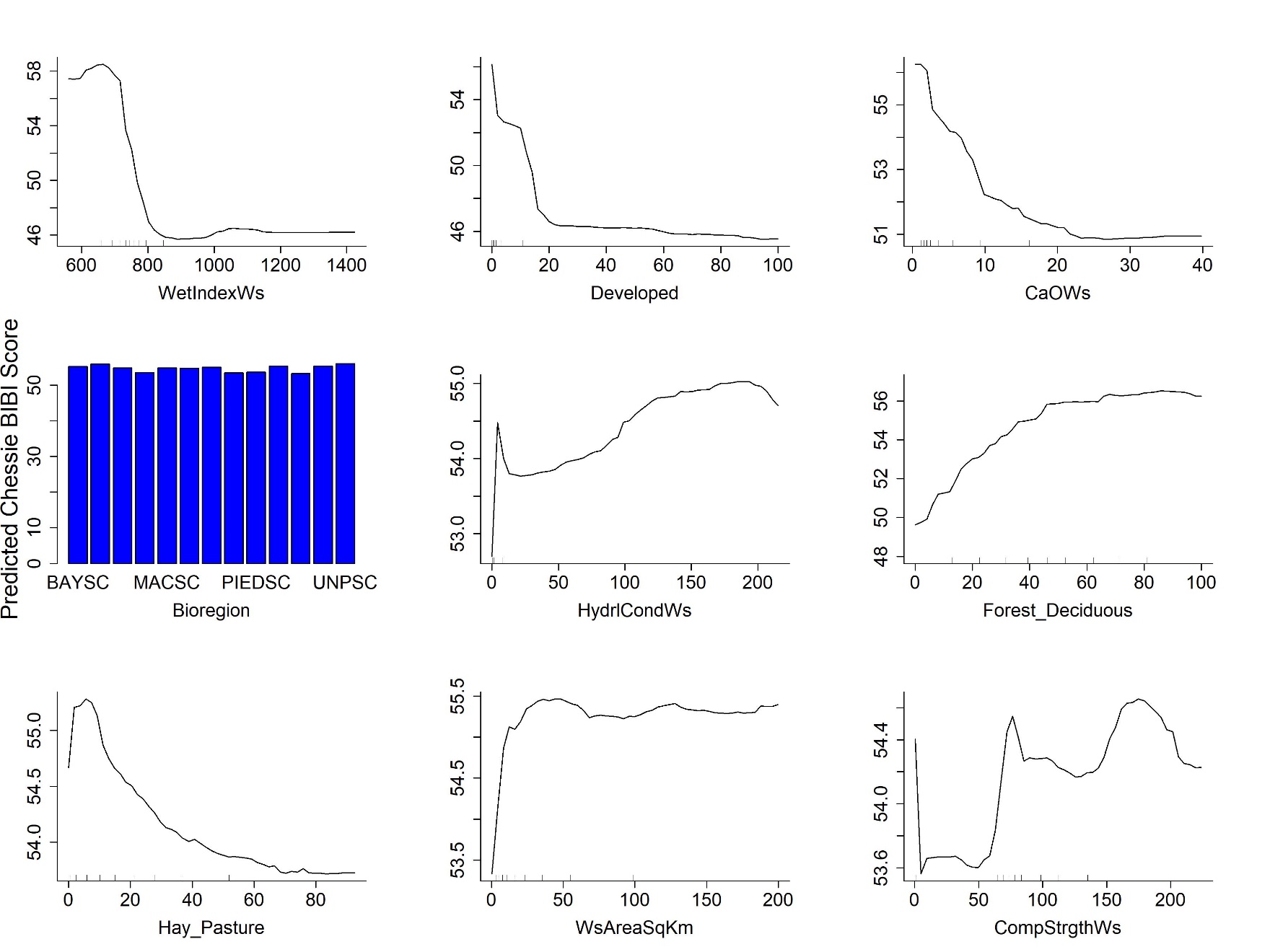


Figure S14. Partial dependence plots for the top nine important non-climate variables in the random forests model.


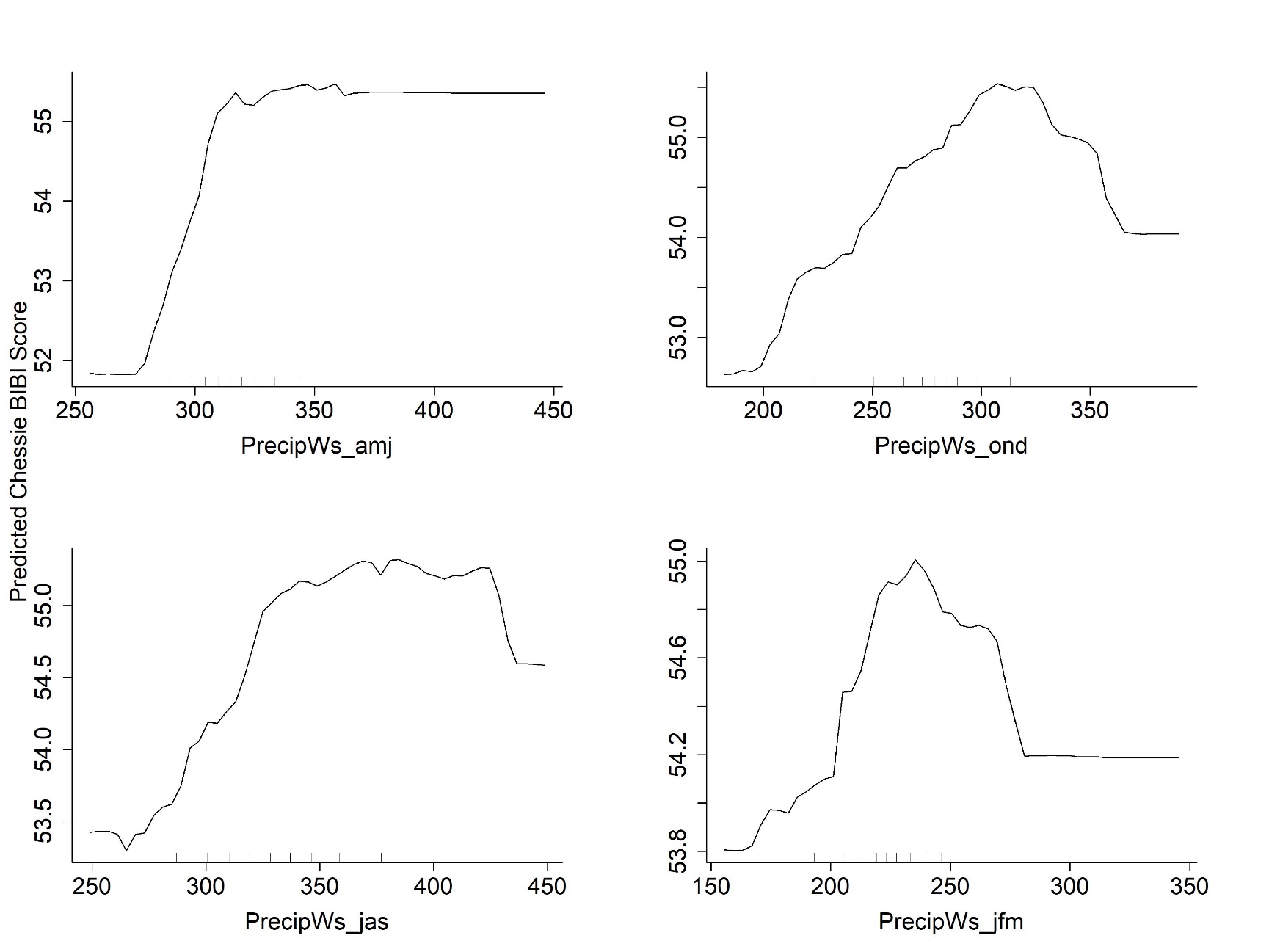


Figure S15. Partial dependence plots for the four seasonal total precipitation covariates in the random forests model in order of importance.


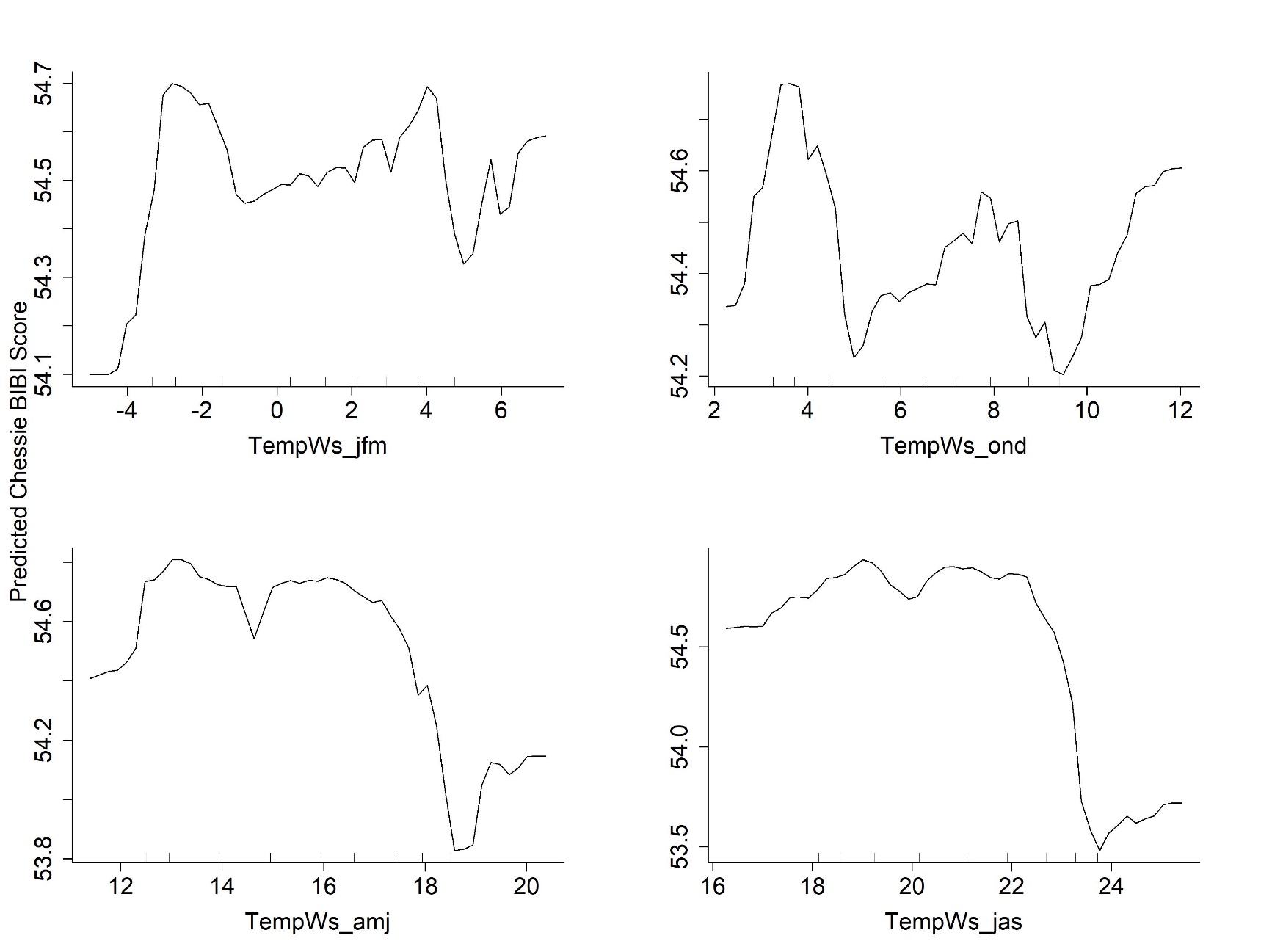


Figure S16. Partial dependence plots for the four seasonal average temperature covariates in the random forests model in order of importance.


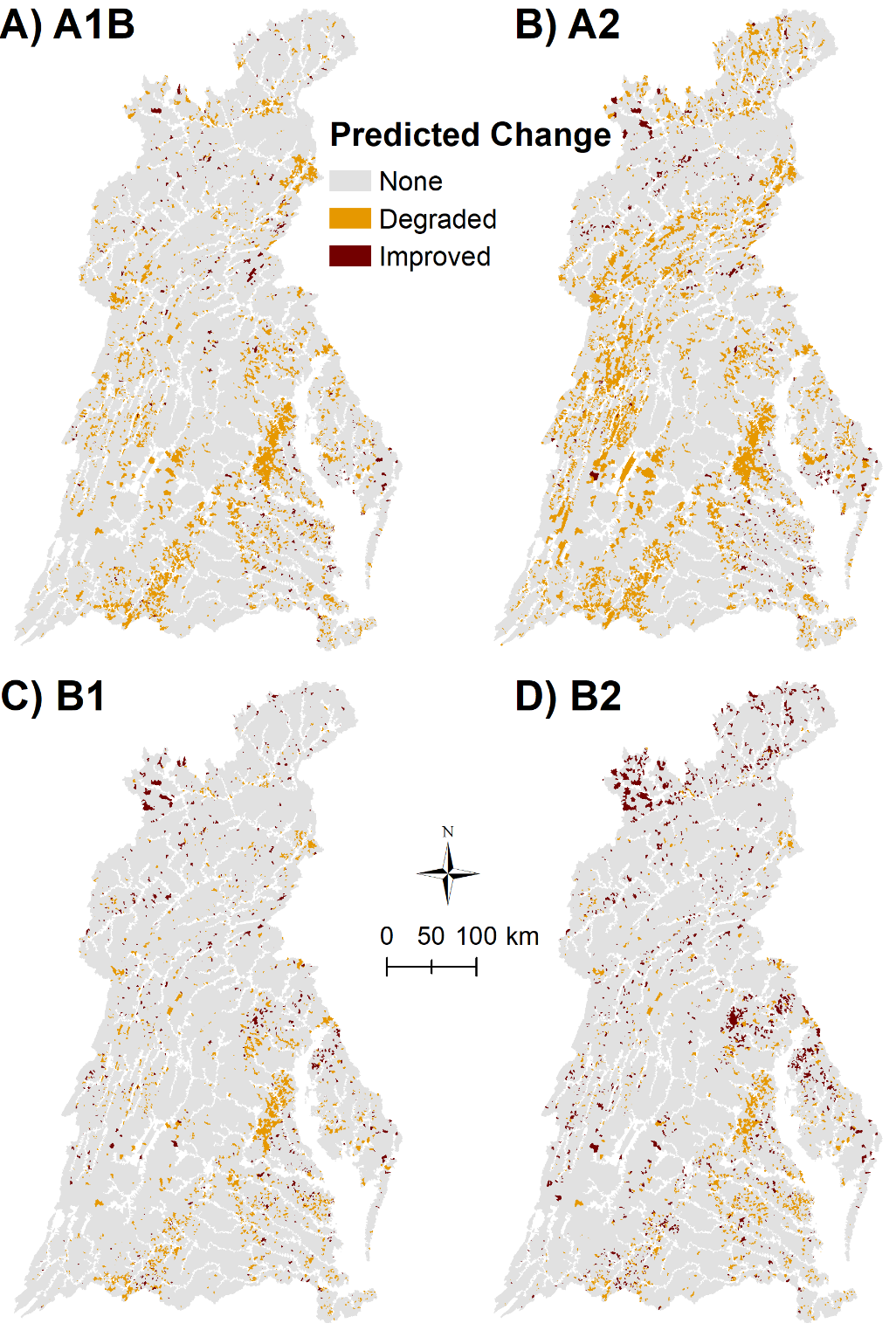


Figure S17. Maps showing NHDPlusv2 catchments with predicted change in stream conditions in 2090 under A1B (A), A2 (B), B1 (C), and B2 (D) land-use and baseline climate projection in the Chesapeake Bay watershed. Coding convention: “None” = a stream reach was predicted in the same condition in baseline and 2090 scenarios (e.g., Poor to Poor), “Degraded” = a stream reach was predicted at a lower category in 2090 (e.g., Good to Fair), and “Improved” = a stream reach was predicted at a higher category in 2090 (Fair to Good).


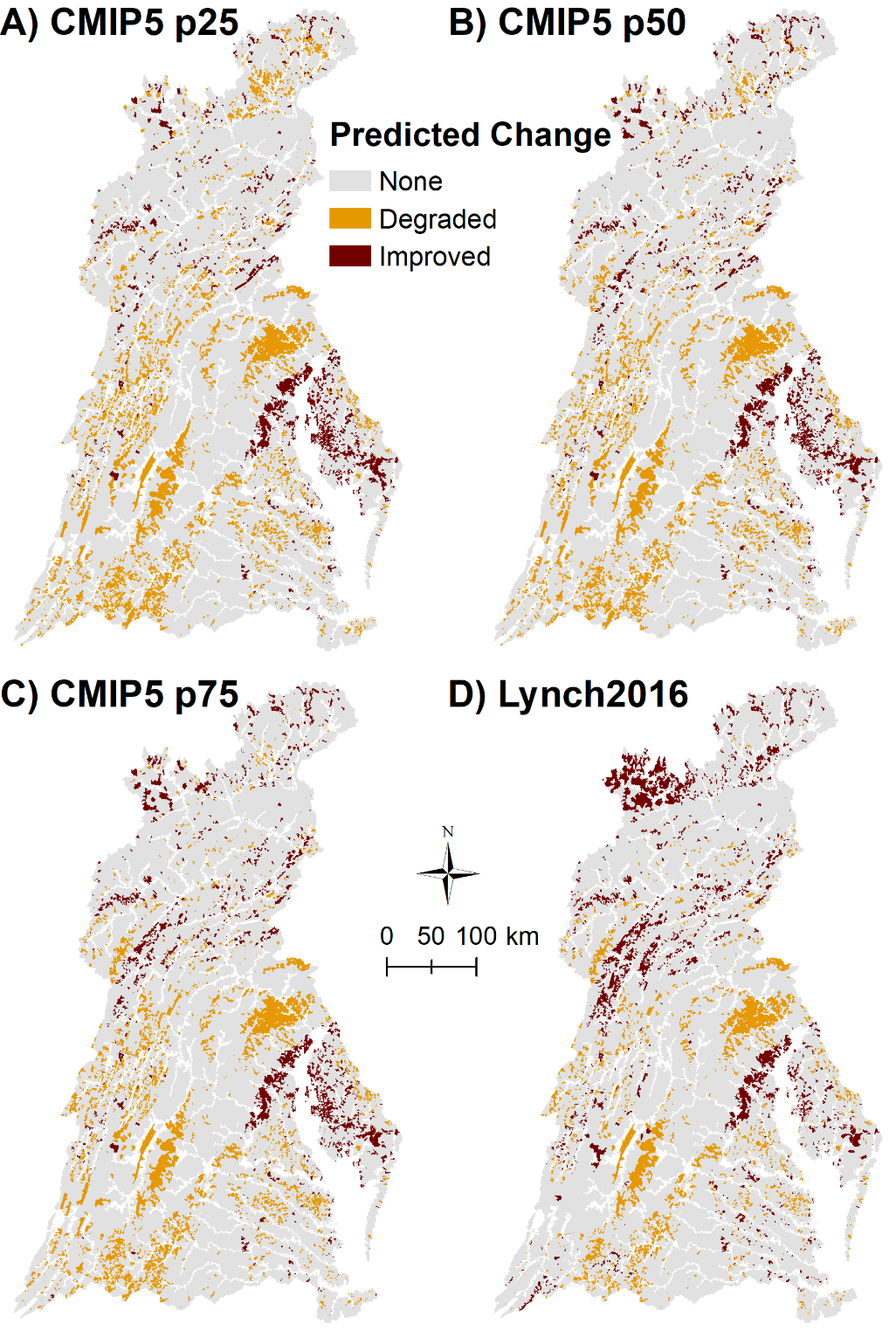


Figure S18. Maps showing NHDPlusv2 catchments with predicted change in stream conditions in 2090 under CMIP5 p25 (A), CMIP5 p50 (B), CMIP5 p75 (C), and Lynch2016 (D) climate and baseline land-use projection in the Chesapeake Bay watershed. Coding convention: “None” = a stream reach was predicted in the same condition in baseline and 2090 scenarios (e.g., Poor to Poor), “Degraded” = a stream reach was predicted at a lower category in 2090 (e.g., Good to Fair), and “Improved” = a stream reach was predicted at a higher category in 2090 (Fair to Good).


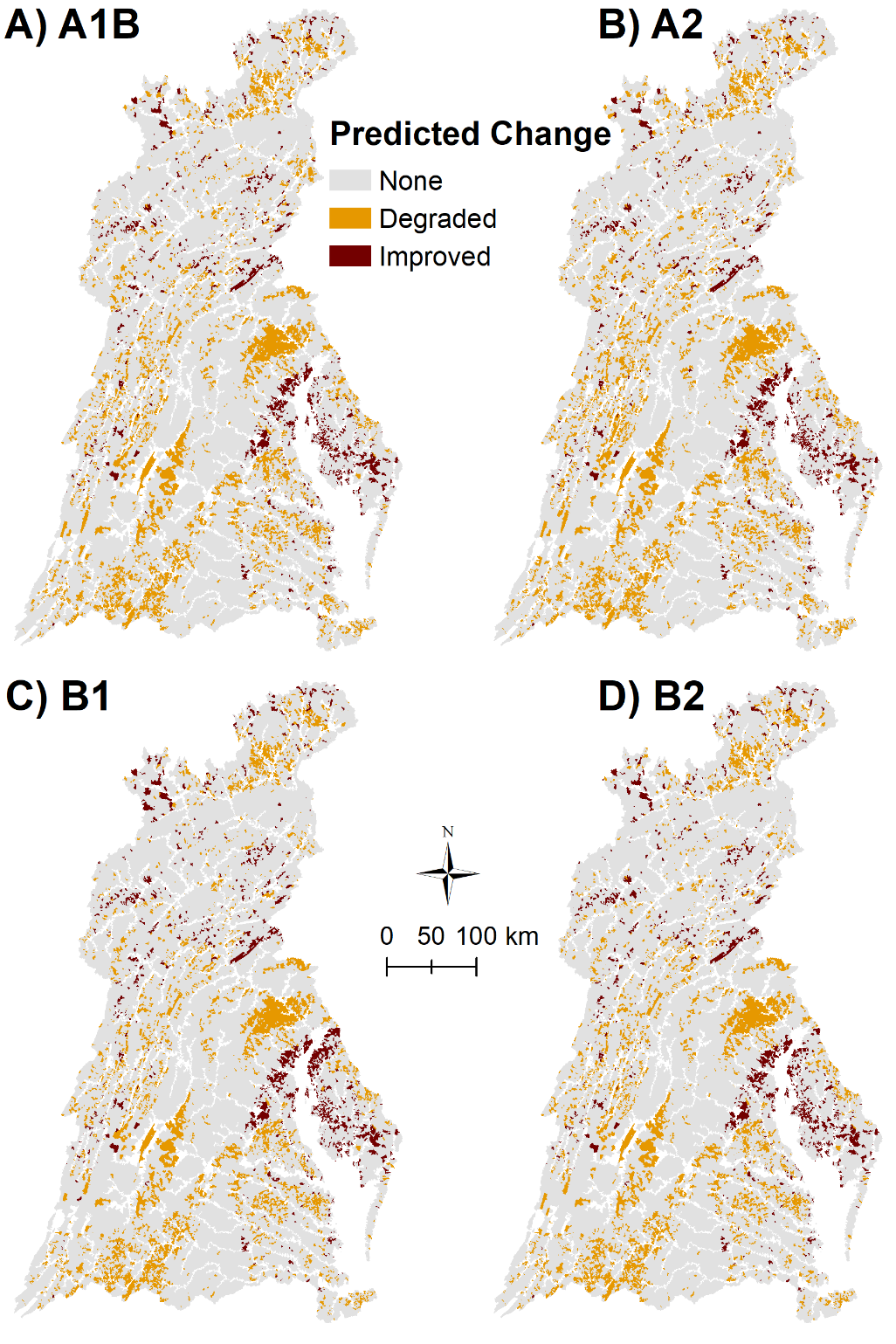


Figure S19. Maps showing NHDPlusv2 catchments with predicted change in stream conditions in 2030 under CMIP5 p50 climate projections and A1B (A), A2 (B), B1 (C), and B2 (D) land-use projections in the Chesapeake Bay watershed. Coding convention: “None” = a stream reach was predicted in the same condition in baseline and 2030 scenarios (e.g., Poor to Poor), “Degraded” = a stream reach was predicted at a lower category in 2030 (e.g., Good to Fair), and “Improved” = a stream reach was predicted at a higher category in 2030 (Fair to Good).


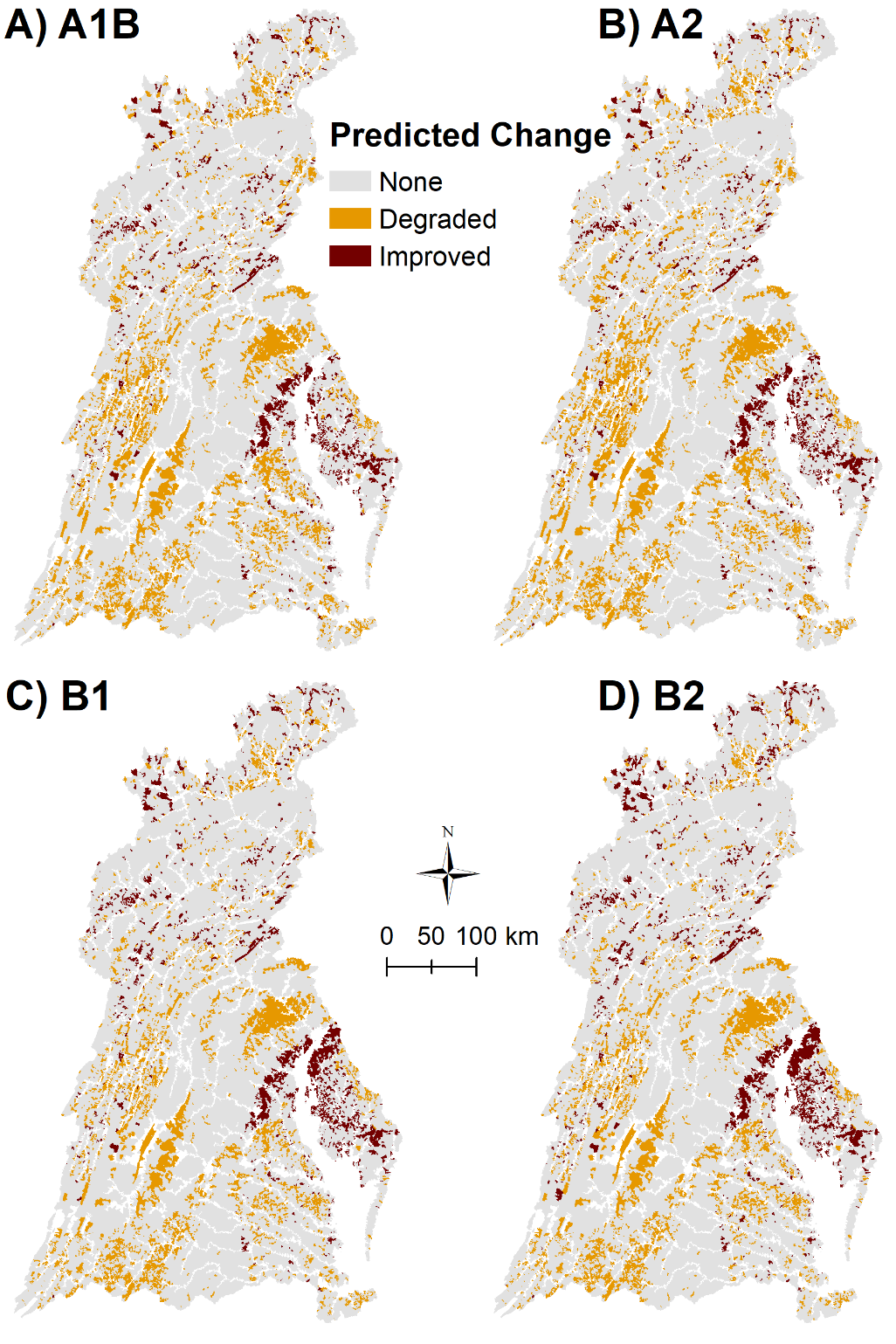


Figure S20. Maps showing NHDPlusv2 catchments with predicted change in stream conditions in 2060 under CMIP5 p50 climate projections and A1B (A), A2 (B), B1 (C), and B2 (D) land-use scenarios in the Chesapeake Bay watershed. Coding convention: “None” = a stream reach was predicted in the same condition in baseline and 2060 scenarios (e.g., Poor to Poor), “Degraded” = a stream reach was predicted at a lower category in 2060 (e.g., Good to Fair), and “Improved” = a stream reach was predicted at a higher category in 2060 (Fair to Good).


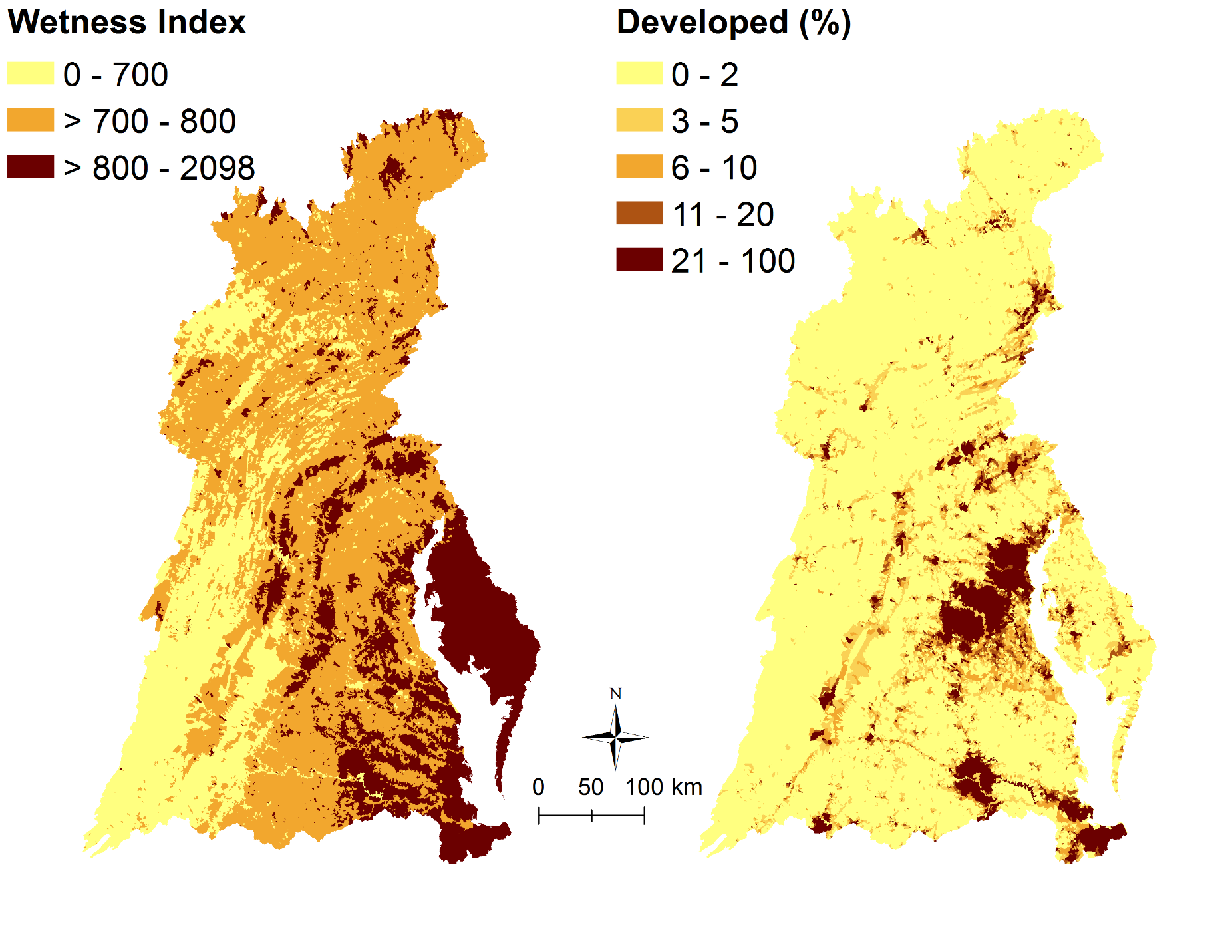


Figure S21. Maps showing topographic wetness index and percentage of developed land cover for the Chesapeake watershed.


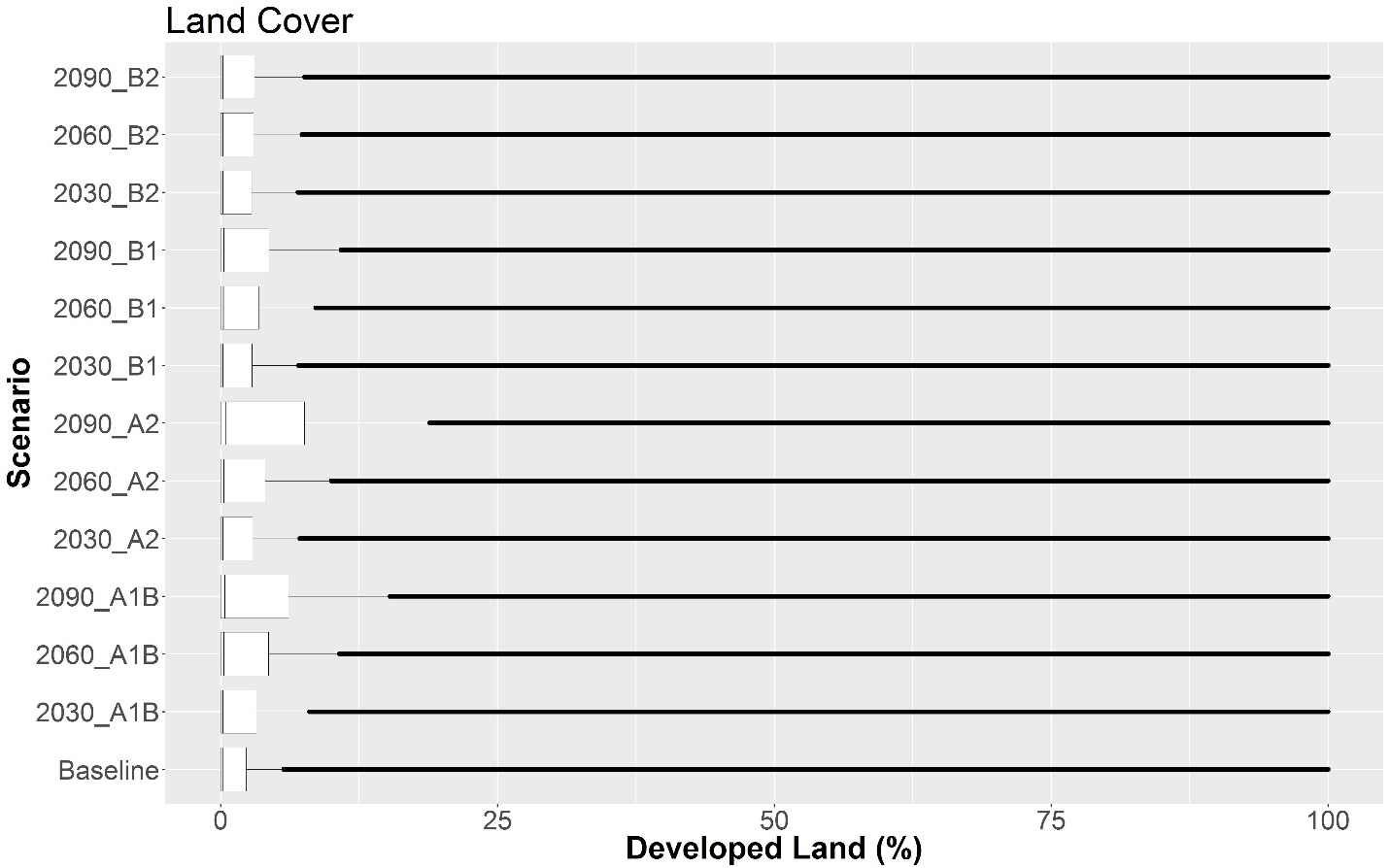


Figure S22. Distributions of upstream watershed percentage of developed land in NHDplusV2 catchments for baseline (2005) and each land-use scenario for 2030, 2060, and 2090.


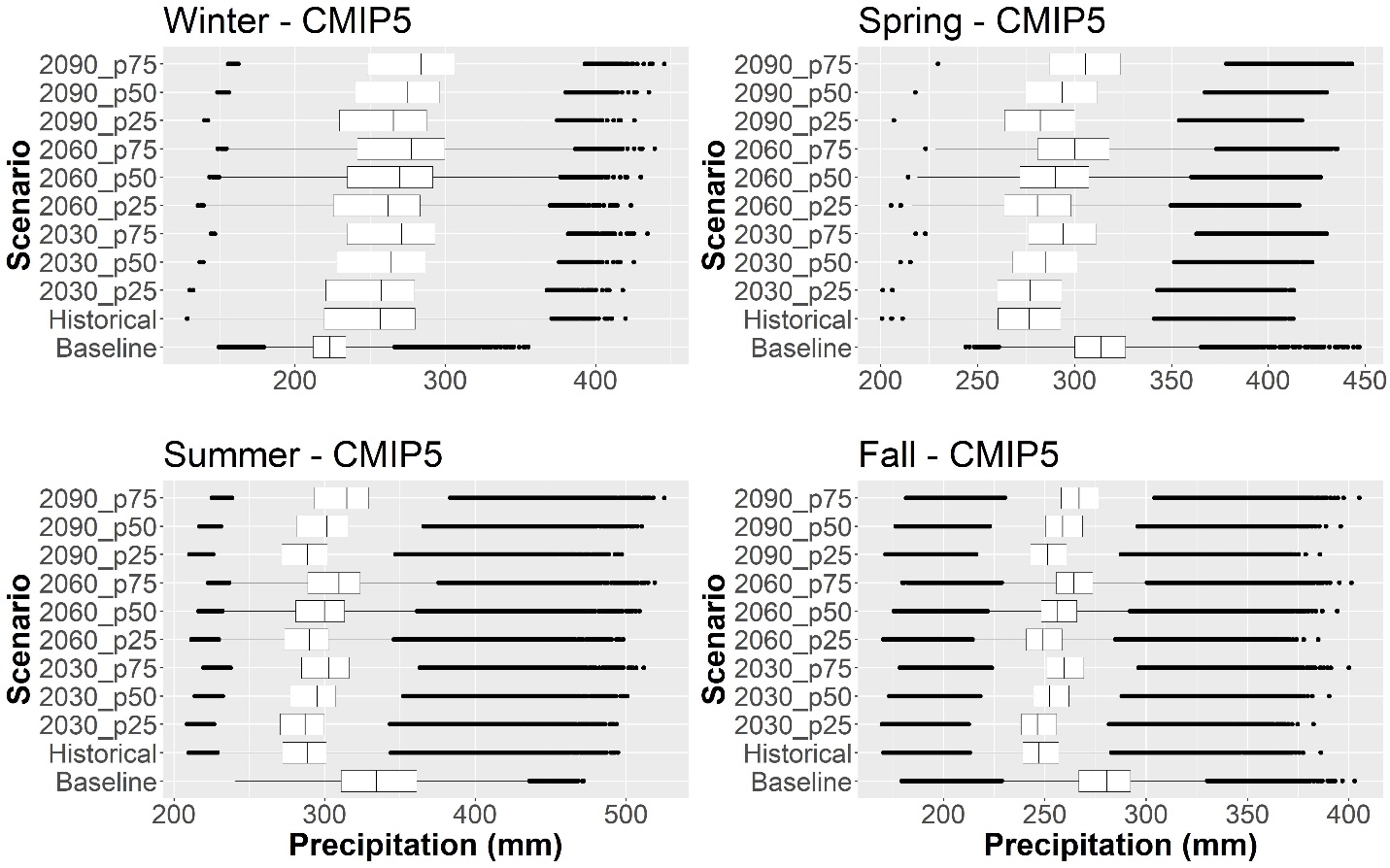


Figure S23. Distributions of upstream watershed total precipitation (CMIP5 projections) in NHDplusV2 catchments for baseline (2000-2011), historical (1980-1999), and each land-use scenario for 2030, 2060, and 2090.


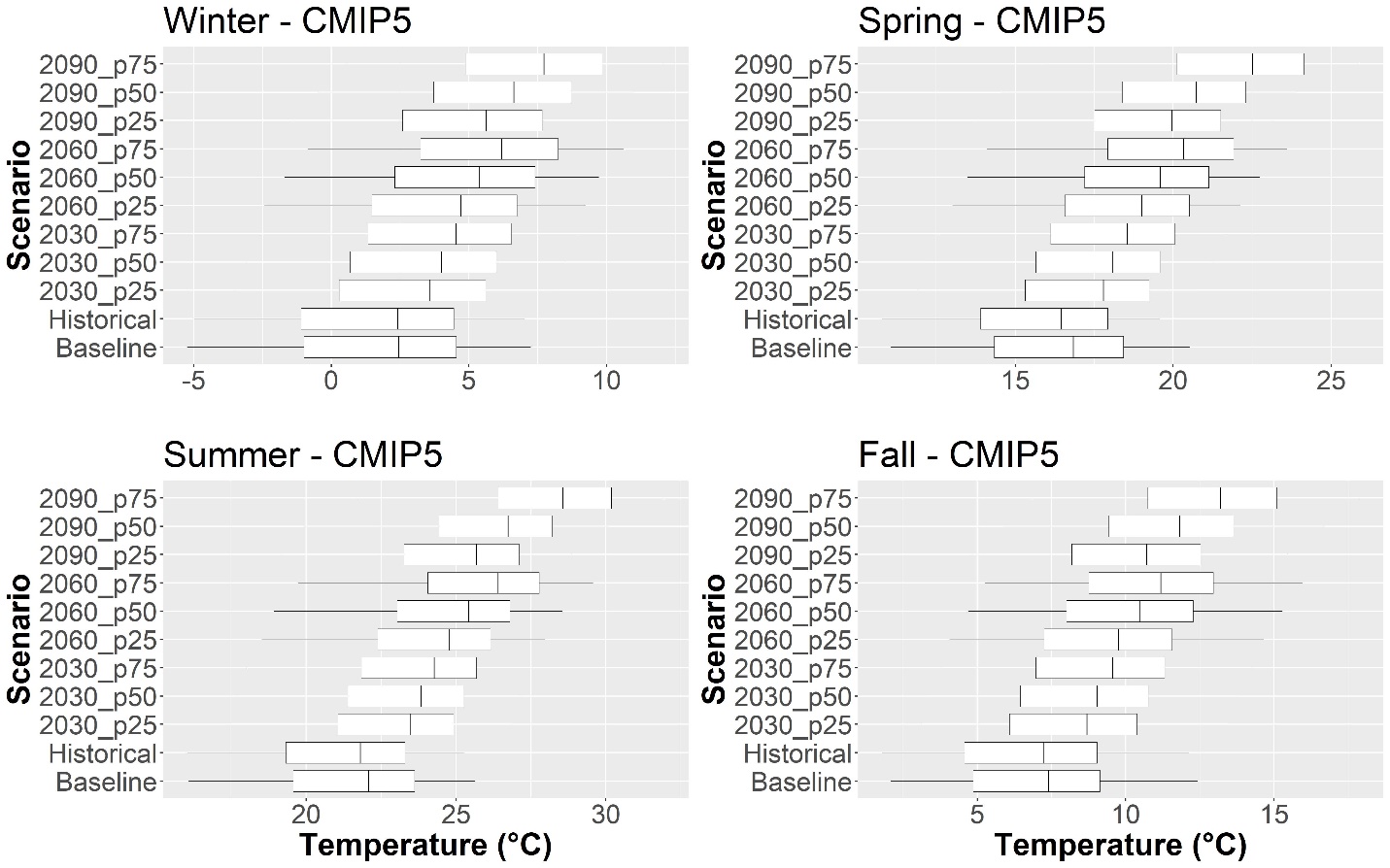


Figure S24. Distributions of upstream watershed average temperature (CMIP5 projections) in NHDplusV2 catchments for baseline (2000-2011), historical (1980-1999), and each land-use scenario for 2030, 2060, and 2090.
